# Economic Burden of Health Conditions Associated With Adverse Childhood Experiences Among US Adults

**DOI:** 10.1001/jamanetworkopen.2023.46323

**Published:** 2023-12-06

**Authors:** Cora Peterson, Maria V. Aslam, Phyllis H. Niolon, Sarah Bacon, Mark A. Bellis, James A. Mercy, Curtis Florence

**Affiliations:** 1 National Center for Injury Prevention and Control, Centers for Disease Control and Prevention, Atlanta, Georgia; 2 Centre for Public Health, Liverpool John Moores University, Liverpool, United Kingdom

## Abstract

**Importance:**

Adverse childhood experiences (ACEs) are preventable, potentially traumatic events in childhood, such as experiencing abuse or neglect, witnessing violence, or living in a household with substance use disorder, mental health problems, or instability from parental separation or incarceration. Adults who had ACEs have more harmful risk behaviors and worse health outcomes; the economic burden associated with these issues is uncertain.

**Objective:**

To estimate the economic burden of ACE-associated health conditions among US adults.

**Design, Setting, and Participants:**

In this economic evaluation, regression models of cross-sectional survey data from the 2019-2020 Behavioral Risk Factor Surveillance System (BRFSS) and previous studies were used to estimate ACE population-attributable fractions (PAFs) (ie, the fraction of total cases associated with a specific exposure) for selected health outcomes (anxiety, arthritis, asthma, cancer, chronic obstructive pulmonary disease, depression, diabetes, heart disease, kidney disease, stroke, and violence) and risk factors (heavy drinking, illicit drug use, overweight and obesity, and smoking) among the 2019 US adult population. Adverse childhood experience PAFs were used to calculate the proportion of total condition-specific medical spending and lost healthy life-years related to ACEs using Global Burden of Disease Study data. Data analysis was performed from September 10, 2021, to November 29, 2022.

**Exposure:**

Adverse childhood experiences (age <18 years).

**Main Outcomes and Measures:**

Monetary valuation of ACE-associated morbidity and mortality using standard US value of statistical life methods and presented in terms of annual and lifetime per affected person and total population estimates at the national and state levels.

**Results:**

A total of 820 673 adults, representing 255 million individuals, participated in the BRFSS in 2019 and 2020. An estimated 160 million of the total 255 million US adult population (63%) had 1 or more ACE, associated with an annual economic burden of $14.1 trillion ($183 billion in direct medical spending and $13.9 trillion in lost healthy life-years). This was $88 000 per affected adult annually and $2.4 million over their lifetimes. The lifetime economic burden per affected adult was lowest in North Dakota ($1.3 million) and highest in Arkansas ($4.3 million). Twenty-two percent of adults had 4 or more ACEs and comprised 58% of the total economic burden—the estimated per person lifetime economic burden for those adults was $4.0 million.

**Conclusions and Relevance:**

In this cross-sectional analysis of the US adult population, the economic burden of ACE-related health conditions was substantial. The findings suggest that measuring the economic burden of ACEs can support decision-making about investing in strategies to improve population health.

## Key Points

**Question:**

What is the economic burden of health conditions associated with adverse childhood experiences (ACEs) among US adults?

**Findings:**

In this economic evaluation of the US adult population, 63% of adults had ACEs. The national economic burden of ACE-related adult health conditions was $14.1 trillion annually ($183 billion in direct medical spending and $13.9 trillion in lost healthy life-years), or $88 000 per affected adult annually and $2.4 million over their lifetimes.

**Meaning:**

The findings of this study suggest that quantifying the economic burden associated with childhood adversity may support decision-making about investing in strategies to improve population health.

## Teaser

This economic evaluation evaluates the economic burden associated with adverse childhood experiences in adults.

## Introduction

Adverse childhood experiences (ACEs) are preventable, potentially traumatic events that occur in childhood, such as experiencing abuse or neglect, witnessing violence, or growing up in a household with substance use disorder, mental health problems, or instability due to parental separation or incarceration.^1^ These experiences have a documented dose-response association with negative adult health outcomes and risk behaviors—meaning the more ACEs a person has, the higher their risk for outcomes, such as depression, substance use disorder, and smoking, that remain after adjusting for household socioeconomic status during childhood.^2^ Research has estimated that one of the pathways by which ACEs affect these outcomes is prolonged activation of the stress-response system, which can harm the nervous, endocrine, and immune systems, leading to acute and lifelong consequences.^3^ Adverse childhood experience prevention strategies are associated with higher academic achievement and reductions in depression, suicidal behavior, arrest and incarceration rates, and substance use in adolescence and adulthood.^1,3^

Previous research has identified a higher prevalence of injuries, self-reported disability, and premature mortality as well as worse mental health, worse maternal health outcomes, and more infectious disease, chronic disease, and risk behaviors, such as substance use disorder, among adults with ACEs.^3,4,5^ Previous cross-sectional studies have estimated a substantial ACE-associated health economic burden in adulthood in Europe and North America,^6,7^ the UK,^8^ and California.^9^ To our knowledge, similar economic estimates do not yet exist for other US states or for the US in total, although the ACE-related health burden among adults is extensively documented.^10,11,12,13,14,15,16^ This study aimed to estimate the economic burden of health conditions associated with ACEs among US adults.

## Methods

This cross-sectional economic evaluation followed the Strengthening the Reporting of Observational Studies in Epidemiology (STROBE) reporting guideline and applicable elements of the Consolidated Health Economic Evaluation Reporting Standards (CHEERS) reporting guideline.^17^ This analysis used the societal perspective: tangible and intangible costs to multiple payers. All costs are reported as 2019 US dollars. Annual estimates used a 1-year time horizon and per affected person lifetime estimates were based on annual costs in combination with average US life expectancy. This study used publicly available data; it did not constitute human research and does not require institutional review board review or exemption according to the Common Rule (45 CFR §46). This study comprised an original analysis of the most comprehensive cross-sectional survey data source in the US for ACEs and adult health outcomes: the Behavioral Risk Factor Surveillance System (BRFSS).^15,18^ The BRFSS is an annual, random-digit-dialed landline and mobile telephone survey of noninstitutionalized US adults. Overall, 418 461 persons participated in the 2019 and 402 212 in the 2020 BRFSS surveys (median state-level response rates: 49.4% vs 47.9%).^19^

### Statistical Analysis

The analysis was conducted from September 10, 2021, to November 29, 2022, in a series of steps that incorporated multiple data sources. First, combined 2019-2020 BRFSS data including modeled small area estimates as described in a previous study provided each state’s estimated prevalence of 8 ACEs (physical, emotional, or sexual abuse; household member substance use disorder, incarceration, or mental illness; parental divorce; or witnessing intimate partner violence) in terms of ACE count (0, 1, 2-3, ≥4) per US adult (≥18 years) (unpublished study by M.V.A. et al). Because just 37 jurisdictions included BRFSS ACE module questions during 2019-2020 and in each state 14% to 33% of the respondents skipped 1 or more ACE survey question, the small area estimates approach (a statistical process that used actual survey responses to generate modeled responses if data were missing^20^) from the unpublished study provided a way to use the most recent BRFSS ACEs data to analyze all states and include more respondents. There was further support for this approach in a recent analysis of state-level BRFSS ACEs data spanning 2011-2020 (the minimum timeline to include data from all states), which reported that respondents who skipped some ACEs questions (27% of respondents) had a higher prevalence of individual ACEs compared with survey respondents who answered all ACE questions.^16^ Estimated proportions of US adults with ACEs by count including small area estimates (unpublished study by M.V.A. et al) were applied to 2019 US Census Bureau population counts.^21^

Second, we used survey-weighted logistic regression models (Stata, version 17 logit; StataCorp) of the same BRFSS ACEs small area estimates data by US state to estimate adjusted odds ratios (AORs) for adults’ ACE count and selected self-reported current health outcomes (arthritis, asthma, cancer [excluding skin cancer], chronic obstructive pulmonary disease, depression, diabetes, heart disease, kidney disease, and stroke) and risk factors for ill health (heavy drinking, overweight or obesity, and smoking) (eTable 1 in Supplement 1 reports health condition definitions in source data).^6,8,9,15^ Models controlled for respondent characteristics as self-reported in BFRSS (sex, age, race and ethnicity, educational level, marital status, current employment status, and metropolitan status) and models of health outcomes (eg, cancer) controlled for the analyzed risk factors (eg, smoking). Models controlled for race and ethnicity because previous research has reported different prevalence of both ACEs and chronic health conditions among adults by race and ethnicity.

Third, ACE population-attributable fractions (PAF) (Stata version 17 punaf; StataCorp)^22^ by US state were estimated when AORs indicated statistically significant higher odds (*P* < .05) of the analyzed conditions among adults with ACEs. Population-attributable fraction is the fraction of all cases of an adverse condition in a population attributable to a specific exposure; despite the causality implied by the term attributable, PAFs based on observational data—as in the present study—are not definitively causal.^23^ Reference sources for the disease burden estimates to which the ACE PAFs were applied were already adjusted for other health issues (eg, the source for medical spending estimates—as described in the next paragraph—for depression excluded costs for coexisting chronic diseases)^24^ and, therefore, consistent with previous similar studies, no other adjustment was applied.^8,9^ However, ACE PAFs adjusted for all analyzed health outcomes are also reported for reference. Adverse childhood experience PAFs for anxiety, interpersonal violence, and illicit drug use (which have documented associations with ACEs but are not measured in the BRFSS) from population-based analysis similar to the US BFRSS in England were added uniformly to all states.^8^

Fourth, ACE PAFs were multiplied by estimates of total annual medical spending and number of disability-adjusted life-years (DALY), a measure of lost life-years owing to ill health, disability, or early death, associated with the analyzed conditions among adults (aged ≥20 years) to estimate each state’s ACE medical spending and ACE DALY burden. Total US medical spending estimates (public insurance including Medicare, Medicaid, and other government programs; private insurance; or out-of-pocket payments) by health condition were inflated to 2019 US dollars (from original reporting as 2016 US dollars), and apportioned by US state using total medical spending by state of residence from the US Centers for Medicare & Medicaid Services.^24,25,26^ Disability-adjusted life-years by health condition for each US state were obtained from the 2019 Global Burden of Disease (GBD) study online query system.^27^ Each DALY (which represents the loss of 1 year of equivalent full health) was valued at $540 000.^28,29^ This value from the US Department of Health and Human Services is derived from quality-adjusted life expectancy and value of statistical life, a monetary estimate of the collective value placed on mortality risk reduction as derived in research studies through revealed preferences (eg, wage differences for dangerous occupations) or stated preferences from surveys soliciting individual persons’ willingness to pay for mortality risk reduction.

Study outcome measures by ACE count were number of adults with ACEs, ACE PAFs for the analyzed health conditions, ACE annual medical spending burden, ACE DALY annual economic burden, ACE total annual economic burden, and ACE per person (affected adult) total annual economic burden and lifetime economic burden. The ACE total annual economic burden was the sum of annual ACE DALY and ACE medical spending estimates. The ACE per person total annual economic burden was the ACE total annual economic burden divided by ACE prevalence (number of adults with ACEs). The ACE per person lifetime economic burden was the ACE per person total annual economic burden estimate applied to the number of years from age 18 to 79 years (current US life expectancy) and discounted 3% annually^30^ (a standard rate for valuing future health states) to present value. State-level calculation examples are reported in eTable 2 in Supplement 1 and all underlying state-level data are presented in eTables 3 to 6 in Supplement 1.

## Results

A total of 820 673 adults, representing 255 million individuals, participated in the BRFSS in 2019 and 2020. An estimated 160 million of the total 255 million US adult population (63%) had 1 or more ACE (Table 1). This includes an estimated 46 million adults (18%) with 1 ACE, 56 million adults (22%) with 2 to 3 ACEs, and 57 million adults (22%) with 4 or more ACEs. Higher ACE count corresponded with a higher prevalence of most of the analyzed conditions; for example, less than 5% of adults with 0 ACEs had depression compared with more than 7% of adults with 1 ACE, more than 18% of adults with 2 to 3 ACEs, and 48% of adults with 4 or more ACEs (Table 2). Adjusted odds ratios for all analyzed conditions indicated a significantly higher prevalence among adults with ACEs (Table 2). For example, the AOR for smoking among adults with 4 or more ACEs was 6.48 (95% CI, 6.20-6.78), indicating that adults with that ACE count were more than 6 times as likely to smoke as other adults. Depression had the highest (78%) and overweight or obesity had the lowest (3%) ACE PAF among US adults (Table 2; state-level data in eTable 3 in Supplement 1). As expected, ACE PAFs for health outcomes when adjusted for all other analyzed health outcomes were consistently lower than unadjusted PAFs. For example, controlling for heart disease, diabetes, and all other analyzed health conditions, the adjusted ACE PAF for stroke indicated that ACEs were associated with an estimated 10% of stroke prevalence compared with an unadjusted estimate of 21% (Table 2). The estimated annual ACE DALY economic burden by health condition was highest for smoking; ACEs were associated with 55% of smoking among US adults, with an annual DALY economic burden of $4.1 trillion (Table 2; eTable 4 in Supplement 1). The estimated annual ACE medical spending burden by health condition was highest for depression, with annual medical spending of $50 billion (Table 2; eTable 5 in Supplement 1).

**Table 1.** ACE Prevalence Among US Adults^a^

State	Population aged ≥18 y, No.	ACE count per person, No. (%)			
		1	2-3	≥4	Any
US	255 200 373	46 854 788 (18.4)	56 373 764 (22.1)	57 062 804 (22.4)	160 291 360 (62.8)
Alabama	3 814 879	772 895 (20.3)	772 513 (20.3)	803 032 (21.1)	2 348 440 (61.6)
Alaska	551 562	79 811 (14.5)	113 346 (20.6)	130 996 (23.8)	324 153 (58.8)
Arizona	5 638 481	927 530 (16.5)	1 286 138 (22.8)	1 441 760 (25.6)	3 655 427 (64.8)
Arkansas	2 317 649	456 113 (19.7)	418 104 (18.0)	506 638 (21.9)	1 380 855 (59.6)
California	30 617 582	3 781 272 (12.4)	7 960 572 (26.0)	7 865 657 (25.7)	19 607 500 (64.0)
Colorado	4 499 217	714 026 (15.9)	1 192 742 (26.5)	1 215 688 (27.0)	3 122 457 (69.4)
Connecticut	2 837 847	626 313 (22.1)	595 097 (21.0)	337 704 (11.9)	1 559 113 (54.9)
Delaware	770 192	136 632 (17.7)	169 981 (22.1)	156 503 (20.3)	463 116 (60.1)
District of Columbia	577 581	125 393 (21.7)	140 063 (24.3)	107 372 (18.6)	372 829 (64.6)
Florida	17 247 808	3 584 095 (20.8)	3 232 239 (18.7)	3 603 067 (20.9)	10 419 401 (60.4)
Georgia	8 113 542	1 626 765 (20.1)	1 657 597 (20.4)	1 713 580 (21.1)	4 997 942 (61.6)
Hawaii	1 116 004	231 682 (20.8)	232 575 (20.8)	212 487 (19.0)	676 745 (60.6)
Idaho	1 338 864	262 953 (19.6)	280 090 (20.9)	332 038 (24.8)	875 082 (65.4)
Illinois	9 853 946	2 787 681 (28.3)	2 304 838 (23.4)	1 925 461 (19.5)	7 017 980 (71.2)
Indiana	5 164 245	902 710 (17.5)	1 089 139 (21.1)	1 181 063 (22.9)	3 172 912 (61.4)
Iowa	2 428 229	507 014 (20.9)	506 286 (20.9)	433 196 (17.8)	1 446 496 (59.6)
Kansas	2 213 064	360 951 (16.3)	490 415 (22.2)	572 962 (25.9)	1 424 328 (64.4)
Kentucky	3 464 802	430 328 (12.4)	709 245 (20.5)	965 987 (27.9)	2 105 560 (60.8)
Louisiana	3 561 164	699 413 (19.6)	675 909 (19.0)	766 363 (21.5)	2 141 684 (60.1)
Maine	1 095 370	199 576 (18.2)	273 623 (25.0)	320 396 (29.3)	793 596 (72.4)
Maryland	4 710 993	710 889 (15.1)	927 123 (19.7)	1 092 479 (23.2)	2 730 492 (58.0)
Massachusetts	5 539 703	862 532 (15.6)	1 131 761 (20.4)	1 271 362 (23.0)	3 265 655 (59.0)
Michigan	7 842 924	1 683 092 (21.5)	1 719 169 (21.9)	1 941 124 (24.8)	5 343 384 (68.1)
Minnesota	4 336 475	1 063 304 (24.5)	743 705 (17.2)	755 414 (17.4)	2 562 423 (59.1)
Mississippi	2 277 566	510 175 (22.4)	449 364 (19.7)	388 553 (17.1)	1 348 091 (59.2)
Missouri	4 766 843	1 022 011 (21.4)	1 024 871 (21.5)	1 062 529 (22.3)	3 109 412 (65.2)
Montana	840 190	138 631 (16.5)	205 595 (24.5)	227 187 (27.0)	571 413 (68.0)
Nebraska	1 458 334	303 479 (20.8)	345 479 (23.7)	266 146 (18.3)	915 105 (62.8)
Nevada	2 387 517	321 599 (13.5)	520 717 (21.8)	783 344 (32.8)	1 625 660 (68.1)
New Hampshire	1 104 458	196 704 (17.8)	239 778 (21.7)	240 882 (21.8)	677 364 (61.3)
New Jersey	6 943 612	1 341 506 (19.3)	1 534 538 (22.1)	1 059 595 (15.3)	3 935 640 (56.7)
New Mexico	1 620 991	243 311 (15.0)	401 682 (24.8)	427 293 (26.4)	1 072 286 (66.2)
New York	15 425 262	2 795 058 (18.1)	2 920 002 (18.9)	2 973 991 (19.3)	8 689 050 (56.3)
North Carolina	8 187 369	1 497 470 (18.3)	1 924 032 (23.5)	1 755 372 (21.4)	5 176 874 (63.2)
North Dakota	581 891	123 652 (21.3)	114 749 (19.7)	102 878 (17.7)	341 279 (58.7)
Ohio	9 111 081	1 605 373 (17.6)	2 047 260 (22.5)	2 045 438 (22.5)	5 698 070 (62.5)
Oklahoma	3 004 733	505 096 (16.8)	539 350 (18.0)	816 686 (27.2)	1 861 132 (61.9)
Oregon	3 351 175	652 809 (19.5)	880 689 (26.3)	869 630 (26.0)	2 403 128 (71.7)
Pennsylvania	10 167 376	1 920 617 (18.9)	2 069 061 (20.4)	2 312 061 (22.7)	6 301 740 (62.0)
Rhode Island	854 866	203 287 (23.8)	169 349 (19.8)	144 814 (16.9)	517 450 (60.5)
South Carolina	4 037 531	905 214 (22.4)	832 539 (20.6)	869 280 (21.5)	2 607 034 (64.6)
South Dakota	667 558	115 888 (17.4)	188 719 (28.3)	109 079 (16.3)	413 686 (62.0)
Tennessee	5 319 123	946 272 (17.8)	1 060 101 (19.9)	1 464 355 (27.5)	3 470 728 (65.3)
Texas	21 596 071	3 593 586 (16.6)	5 129 067 (23.8)	4 818 084 (22.3)	13 540 737 (62.7)
Utah	2 274 774	358 277 (15.8)	671 968 (29.5)	560 049 (24.6)	1 590 295 (69.9)
Vermont	509 984	101 844 (20.0)	116 735 (22.9)	117 296 (23.0)	335 875 (65.9)
Virginia	6 674 671	1 515 150 (22.7)	1 400 346 (21.0)	1 121 345 (16.8)	4 036 841 (60.5)
Washington	5 951 832	1 158 227 (19.5)	1 636 754 (27.5)	1 510 575 (25.4)	4 305 555 (72.3)
West Virginia	1 432 580	210 303 (14.7)	262 162 (18.3)	357 572 (25.0)	830 037 (57.9)
Wisconsin	4 555 837	968 115 (21.3)	962 648 (21.1)	889 299 (19.5)	2 820 063 (61.9)
Wyoming	445 025	83 843 (18.8)	93 233 (21.0)	114 994 (25.8)	292 070 (65.6)

Abbreviation: ACE, adverse childhood experience.

^a^ Proportion of adults with ACEs by count from 2019-2020 Behavioral Risk Factor Surveillance System population-weighted survey data (n = 804 641 records; n = 257 057 289 weighted) as reported in the unpublished study by M.V.A. et al and by the US Census Bureau^21^ for population size.

**Table 2.** ACE Annual Economic Burden Among US Adults by Health Condition^a^

Measure	Adults with health condition by ACE No. (%)^b^				AOR by ACE, No. (95% CI)			PAF by ACE, No.^c^					Economic burden, millions, 2019 US $^d^	
	0	1	2-3	≥4	1	2-3	≥4	1	2-3	≥4	Any	Any adjusted^e^	DALY	Medical spending
Anxiety^f^	NA	NA	NA	NA	NA	NA	NA	6.1	12.6	10.4	29.1	NA	261 403	11 693
Arthritis	21 202 674 (22.3)	10 725 061 (22.9)	14 093 441 (25.0)	16 011 823 (28.1)	1.57 (1.52-1.63)	2.12 (2.05-2.19)	3.25 (3.13-3.38)	4.2	8.8	13.3	26.3	19.1	282 037	22 076
Asthma	7 962 867 (8.4)	5 786 566 (12.4)	8 348 954 (14.8)	14 819 210 (26.0)	1.58 (1.50-1.66)	1.95 (1.87-2.05)	3.65 (3.48-3.83)	5.2	10.8	27.4	43.4	34.8	229 150	12 594
Cancer	7 649 667 (8.1)	3 078 360 (6.6)	3 354 239 (6.0)	3 463 712 (6.1)	1.11 (1.05-1.16)	1.08 (1.03-1.13)	1.47 (1.39-1.56)	1.5	1.3	6.1	9.0	3.4	773 668	8713
COPD	3 635 015 (3.8)	2 670 723 (5.7)	3 692 482 (6.6)	5 997 301 (10.5)	1.98 (1.86-2.11)	2.74 (2.58-2.91)	4.78 (4.49-5.09)	7.1	14.2	28.2	49.5	36.6	1 337 062	17 696
Depression	4 498 687 (4.7)	3 331 375 (7.1)	10 288 212 (18.3)	27 538 509 (48.3)	2.12 (2.00-2.24)	6.63 (6.30-6.97)	24.92 (23.59-26.33)	3.8	20.1	54.0	77.9	76.0	960 144	49 663
Diabetes	11 778 209 (12.4)	4 563 656 (9.7)	5 941 795 (10.5)	5 535 092 (9.7)	1.00 (0.95-1.05)	1.27 (1.21-1.32)	1.30 (1.24-1.37)		3.6	3.5	7.1	0.6	158 606	8106
Heart disease	4 014 651 (4.2)	1 855 450 (4.0)	2 114 016 (3.8)	2 054 261 (3.6)	1.28 (1.20-1.37)	1.60 (1.50-1.71)	2.06 (1.92-2.22)	3.6	7.9	10.3	21.9	8.6	1 182 499	20 739
Kidney disease	2 553 053 (2.7)	1 302 563 (2.8)	1 871 609 (3.3)	1 871 660 (3.3)	1.43 (1.32-1.55)	1.97 (1.82-2.13)	2.10 (1.91-2.30)	5.0	12.3	12.4	29.8	18.6	241 959	5967
Stroke	3 037 089 (3.2)	1 255 708 (2.7)	1 634 839 (2.9)	2 168 387 (3.8)	1.16 (1.07-1.25)	1.45 (1.35-1.55)	1.97 (1.82-2.13)	1.9	6.1	12.7	20.7	10.4	423 388	9004
Violence^f^	NA	NA	NA	NA	NA	NA	NA	4.4	17.0	22.0	43.4	NA	227 219	4478
Heavy drinking	2 591 016 (2.7)	2 890 940 (6.2)	4 222 395 (7.5)	6 042 951 (10.6)	2.07 (1.95-2.20)	2.59 (2.44-2.75)	3.93 (3.69-4.17)	9.1	16.9	27.7	53.7	NA	1 470 625	4501
Illicit drug use^f^	NA	NA	NA	NA	NA	NA	NA	6.2	15.9	30.6	52.7	NA	1 997 078	6685
OVOB	61 083 443 (64.4)	31 636 353 (67.5)	37 657 674 (66.8)	38 386 148 (67.3)	1.16 (1.12-1.20)	1.20 (1.16-1.23)	1.29 (1.25-1.33)	0.7	1.1	1.5	3.3	NA	225 282	333
Smoking	5 305 414 (5.6)	4 854 156 (10.4)	8 320 768 (14.8)	16 776 464 (29.4)	1.92 (1.82-2.02)	2.96 (2.83-3.09)	6.48 (6.20-6.78)	5.9	14.3	35.0	55.2	NA	4 156 191	1076

Abbreviations: ACE, adverse childhood experience; AOR, adjusted odds ratio; COPD, chronic obstructive pulmonary disorder; DALY, disability-adjusted life-year; NA, not applicable; OVOB, overweight or obesity; PAF, population-attributable fraction.

^a^ Source: 2019-20 Behavioral Risk Factor Surveillance System (BRFSS) population-weighted survey data (n = 804 641 records; n = 257 057 289 weighted) as reported in the unpublished study by M.V.A. et al; Global Burden of Disease Results Tool^27^ for DALY data; Dieleman et al^24^ for medical spending data, adjusted to 2019 US dollars.^25^

^b^ Survey nonresponses excluded.

^c^ ACE PAFs were estimated when AORs indicated statistically significant higher odds (*P* < .05) of the analyzed conditions. “Any” is the sum of PAF by ACE count (1,2-3,4).

^d^ Total economic spending for disability-adjusted life-years, $13 926 313; medical spending, $183 324.

^e^ Unadjusted ACE PAFs for health outcomes were used for the economic burden estimates because disease burden source data were already adjusted for coexisting chronic diseases.^24^ Adjusted PAFs for conditions analyzed with Behavioral Risk Factor Surveillance System data also presented here for reference.

^f^ Population-attributable fraction from Hughes et al^8^ (not generated using original BRFSS source data in the manner of other analyzed conditions).

Adverse childhood experiences were associated with a total annual economic burden of $14.1 trillion (comprising $183 billion in medical spending and $13.9 trillion in lost healthy life-years) for adult health conditions related to ACEs (Table 2). Largely corresponding with population size, ACE total annual economic burden was lowest in North Dakota ($15 billion) and highest in California ($1.5 trillion) (eTable 6 in Supplement 1). The ACE per person annual economic burden was $88 000 per affected adult and $2.4 million over their lifetime; lifetime burden per affected adult was lowest in North Dakota ($1.3 million) and highest in Arkansas ($4.3 million) (Figure; eTable 6 in Supplement 1). Twenty-two percent of adults had 4 or more ACEs and comprised 58% of the US total economic burden; per person economic burden estimates for those adults were correspondingly far higher ($4.0 million, range by state: $2.6 million in North Dakota to $6.8 million in Arkansas) (eTable 6 in Supplement 1).

**Figure.**
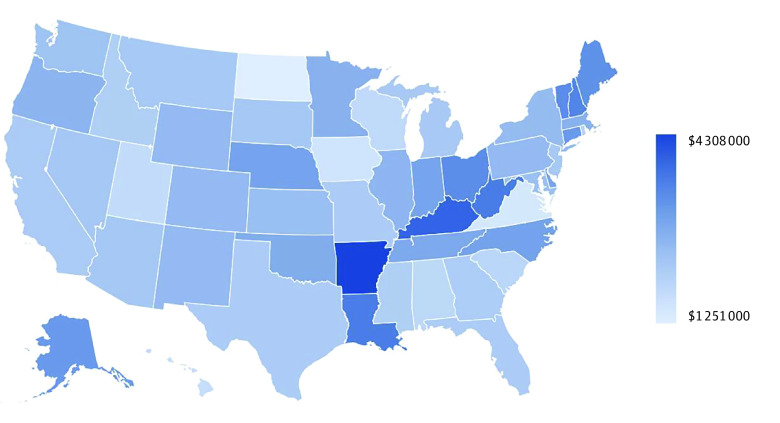
State-Level per Affected Person Lifetime Adverse Childhood Experience Economic Burden Among US Adults in 2019 US Dollars eTable 6 in Supplement 1 reports underlying state-level data.

## Discussion

All states face a substantial economic burden of both direct medical spending and economic loss from reduced healthy life-years for ACE-associated adult health conditions. The variation in states’ estimated ACE economic burden is associated with each state’s total disease burden (eg, smoking-related disability and mortality) and its association with ACEs among adults in the state. State-level variation is expected in that subsequent life experiences, public health or medical interventions, or other contributing factors can mitigate the link between ACEs and adult health. Childhood adversity may be reduced through targeted strategies that create and sustain safe, stable, nurturing relationships and environments for all children and families, and focus the greatest attention on populations and communities that are most likely to experience multiple forms of adversity.

This study supports previous research observing correspondence between a higher ACE count and a greater burden of disease^6,8,9,14^ as well as previous findings of a high ACE-associated adult health economic burden in studies using similar cross-sectional PAF methods in Europe and North America,^6,7^ the UK,^8^ and California.^9^ Medical spending represents financial costs to specific, identifiable payers (including individual persons, health insurance payers, and employers), but most of the economic burden presented herein comes from healthy life-year losses based on the value of statistical life estimates. The value of what is lost in quality and number of life-years is not completely identifiable through financial transactions and thus not as visible as direct costs, such as medical spending or employer costs from lost work productivity. This study valued reduced quality and number of life-years in monetary terms based on recommended US estimates, which are higher than common alternatives, such as 1 to 3 times per capita gross domestic product.^6,7^ An alternative DALY valuation using 2019 US gross domestic product per capita ($65 000) with this study’s data would yield an economic burden estimate of $1.7 trillion (8% of 2019 US gross domestic product).^31^

### Limitations

This study has limitations. The results describe an economic burden of adult health conditions associated with but not definitively and exclusively caused by ACEs. The BRFSS offers population-based US survey data on ACEs among adults but limited analytic opportunities to control for non-ACE factors, for example, environmental or economic, during childhood that also affect adult health. This study controlled for adults’ employment status and educational attainment but not similar household characteristics during childhood. Low household socioeconomic status is associated with both ACEs (eg, parental separation, substance use disorder, and family member incarceration) and adult health conditions analyzed in this study (eg, chronic obstructive pulmonary disease and asthma) by way of factors such as greater exposure to poor air quality among households with lower socioeconomic status. Further interrogation of the association between ACEs and adult health outcomes independent of childhood socioeconomic and other factors is important.

The aforementioned limitations mean that this study’s results could overstate the economic value of preventing ACEs. However, there are multiple reasons that the results presented herein underestimate the economic burden of ACE-associated health conditions. This study addressed only health risk factors and outcomes previously assessed for association with ACEs using BRFSS or similar data that had DALY measures from the GBD study; this study did not investigate, for example, self-harm, suicide attempts, gastrointestinal conditions, musculoskeletal conditions beyond arthritis, or violence perpetration among adults who experienced ACEs. Results reflect the ACE-associated economic burden in adulthood but not childhood and available data on medical spending and DALYs excluded adults aged 18 to 19 years. This study examined the ACE-associated health burden among adults who experienced ACEs themselves but did not attempt to quantify later intergenerational effects.^32^ A focus limited to the health burden underestimates the total economic burden of ACEs; this study’s estimates do not include known ACE-associated socioeconomic outcomes in adulthood, including unemployment, crime, and social deprivation. This study mathematically combined numerous data inputs to produce point estimates of the economic burden but did not attempt to combine measures of dispersion from each data input to directly quantify uncertainty in overall measures.

There are additional study limitations. Global Burden of Disease data and PAF methods are each extensively used but have limitations.^33,34,35^ Criticism of GBD data often refers to countries where health data systems are underdeveloped or incomplete—requiring extensive modeling for complete GBD estimates, which some argue risks undermining incentives to invest in resources to gather real-world data—whereas GBD data sources for US estimates are largely long-standing, relatively comprehensive, and publicly available.^36^ However, GBD data are also criticized for opaque analytic procedures,^33^ and this study did not include verification tasks, such as analysis of original GBD sources, to double-check case counts, nor did we delve into contributing details, such as GBD disability weights, used to calculate DALY morbidity. Interpretation of this study’s PAF results as the proportional reduction in average disease risk that could be achieved by eliminating ACE assumes that exposure has been accurately measured in the study population and that unmeasured factors affecting both ACEs and adult health outcomes (eg, childhood socioeconomic status) are less influential than the effect of ACEs on adult health. The ability to control for additional childhood circumstances could reduce the economic estimate values presented herein.

This analysis assessed the association between ACEs and adult health conditions using self-reported survey data; survey measurement bias, confounding, and reporting bias could have affected the results. The BRFSS does not include institutionalized respondents. Parental separation is typically assessed as an ACE and, even in the absence of other ACEs, has been associated with a range of negative life course outcomes.^37^ However, it is discordant to discuss parental separation in terms of a preventable public health issue in the same manner as other ACEs. Burden estimates by health condition were based on available data for each condition as defined in reference sources and may not reflect total costs; for example, medical spending on overweight or obesity was operationalized as the cost of morbid obesity (the best available estimate for data source consistency but likely an overestimate of unit cost). Converting the estimated ACE annual economic burden to an average lifetime per-person economic burden assumed that population-level association between ACEs and adult health outcomes remains constant over adult lifetimes. Adverse childhood experience PAFs were used in combination with total DALY burden data to estimate the economic burden associated with ACEs; alternative burden of disease estimates that do not rely on PAFs would provide a useful comparison with this study’s approach and results. The results do not address the per-person economic burden of ACEs by demographic characteristics, such as race and ethnicity, due to a lack of applicable medical spending and DALY data; future studies are needed to address these gaps.

## Conclusions

This economic evaluation observed that among the 63% of US adults who had 1 or more ACE, the associated annual economic burden was $14.1 trillion in medical spending and lost healthy life-years from related chronic disease and risk factors for ill health in adulthood. This is $88 000 per affected adult annually and $2.4 million over their lifetimes. The burden was highest among adults who had multiple ACEs. Childhood adversity may be reduced through targeted strategies that strengthen economic supports for families, promote social norms that protect against violence and adversity, teach skills to help parents and youth handle stress and manage emotions and tackle everyday challenges, connect youth to caring adults and activities, and intervene to lessen immediate and long-term harms.^1^ The estimated economic burden of ACEs reported herein, in terms of direct costs such as medical spending or more expansively evaluated in terms of societal cost, may be useful in decision-making on the present value of investment in such strategies to prevent ACEs.

## Notes

Supplement 1. **eTable 1.** Study Definitions for Analyzed Health Conditions **eTable 2.** State-Level Calculation Examples **eTable 3.** State-Level ACE PAF for Adult Health Conditions **eTable 4.** State-Level ACE DALY Economic Burden Among Adults by Health Condition, $ Millions 2019 USD **eTable 5.** State-Level ACE DALY Medical Spending Among Adults by Health Condition, $ Millions 2019 USD **eTable 6.** State-Level Annual and per Person (Affected Adult) Lifetime ACE Economic Burden by ACEs n, 2019 USD

Supplement 2.
Data Sharing Statement
