# Supplementary material for: Economic Burden of Health Conditions Associated With Adverse Childhood Experiences Among US Adults
Source: JAMA Netw Open. 2023 Dec 6;6(12):e2346323. doi: 10.1001/jamanetworkopen.2023.46323 (PMC10701608; doi:10.1001/jamanetworkopen.2023.46323)
Supplement: Supplement 1. — eTable 1. Study Definitions for Analyzed Health Conditions eTable 2. State-Level Calculation Examples eTable 3. State-Level ACE PAF for Adult Health Conditions eTable 4. State-Level ACE DALY Economic Burden Among Adults by Health Condition, $ Millions 2019 USD eTable 5. State-Level ACE DALY Medical Spending Among Adults by Health Condition, $ Millions 2019 USD eTable 6. State-Level Annual and per Person (Affected Adult) Lifetime ACE Economic Burden by ACEs n, 2019 USD [file jamanetwopen-e2346323-s001.pdf]

Supplementary Online Content

Peterson C, Aslam MV, Niolon PH, et al. Estimated economic burden of health conditions associated with adverse childhood experiences among US adults. *JAMA Netw Open*. 2023;6(12):e2346323. doi:10.1001/jamanetworkopen.2023.46323

eTable 1. Study Definitions for Analyzed Health Conditions

eTable 2. State-Level Calculation Examples

eTable 3. State-Level ACE PAF for Adult Health Conditions

eTable 4. State-Level ACE DALY Economic Burden Among Adults by Health Condition, $ Millions 2019 USD

eTable 5. State-Level ACE DALY Medical Spending Among Adults by Health Condition, $ Millions 2019 USD

eTable 6. State-Level Annual and per Person (Affected Adult) Lifetime ACE Economic Burden by ACEs n, 2019 USD

This supplementary material has been provided by the authors to give readers additional information about their work.

© 2023 Peterson C et al. *JAMA Network Open.*

eTable 1. Study definitions for analyzed health conditions

Measure	Data element and source		
	ACE PAF from 2019-20 BRFSS or Hughes (2020)[a]	DALY category (source ID) from Global Burden of Disease[a]	Medical spending category from Dieleman (2020)[a]
Anxiety	Hughes (2020): Are you currently or have you ever been treated for anxiety? Yes, currently; Yes, in the past.	Anxiety disorders (571)	Anxiety disorders
Arthritis	BRFSS: Has a doctor, nurse, or other health professional ever told you that you had some form of arthritis, rheumatoid arthritis, gout, lupus, or fibromyalgia? (HAVARTH4=1)	Osteoarthritis (628)	Osteoarthritis
Asthma	BRFSS: Has a doctor, nurse, or other health professional ever told you that you had asthma? (ASTHMA3=1)	Asthma (515)	Asthma
Cancer	BRFSS: Has a doctor, nurse, or other health professional ever told you that you had any other types of cancer [not skin cancer]? (CHCOCNCR=1)	Neoplasms (410) minus Malignant skin melanoma (459) and non-melanoma skin cancer (462)	Neoplasms minus Malignant skin melanoma and Nonmelanoma skin cancer
COPD	BRFSS: Has a doctor, nurse, or other health professional ever told you that you had chronic obstructive pulmonary disease, C.O.P.D., emphysema or chronic bronchitis? (CHCCOPD2=1)	Chronic obstructive pulmonary disease (509)	COPD
Depression	BRFSS: Has a doctor, nurse, or other health professional ever told you that you had a depressive disorder (including depression, major depression, dysthymia, or minor depression)? (ADDEPEV3=1)	Depressive disorders (567)	Depressive disorders
Diabetes	BRFSS: Has a doctor, nurse, or other health professional ever told you that you had diabetes (DIABETE4=1)	Diabetes mellitus type 2 (976)	Diabetes mellitus
Heart disease	BRFSS: Has a doctor, nurse, or other health professional ever told you that you had angina or coronary heart disease? (CVDCRHD4=1)	Ischemic heart disease (493) plus Hypertensive heart disease (498)	Ischemic heart disease plus Hypertensive heart disease
Kidney disease	BRFSS: Not including kidney stones, bladder infection or incontinence, were you ever told you have kidney disease? (CHCKDNY2=1)	Chronic kidney disease (589) minus chronic kidney disease due to diabetes mellitus (997,998)	Chronic kidney diseases
Stroke	BRFSS: Has a doctor, nurse, or other health professional ever told you that you had a stroke? (CVDSTRK3=1)	Stroke (494)	Cerebrovascular disease
Violence	Hughes (2020): How many times have you been physically hit in the past 12 months? Once; 2 or 3 times; More than 3 times.	Interpersonal violence (724)	Interpersonal violence
Heavy drinking	BRFSS: Heavy drinkers (adult men having more than 14 drinks per week and adult women having more than 7 drinks per week) (_RFDRHV7=2)	Alcohol use (102)	Alcohol use disorders
Illicit drug use	Hughes (2020): How often, if ever, have you used heroin or crack cocaine? Used but not in the last 12 months; Used in the past 12 months.	Drug use disorders (103)	Drug use disorders
OVOB	BRFSS: BMI ≥25 kg/m2 (BMI5CAT=3 or 4)	High body mass index (108)	Morbid obesity
Smoking	BRFSS: Have you smoked at least 100 cigarettes in your entire life? Do you now smoke cigarettes every day, some days, or not at all? (SMOKER3=1 or 2)	Smoking (99)	Tobacco intervention

Abbreviations: ACE = Adverse childhood experience, BMI = Body mass index, BRFSS = Behavioral Risk Factors Surveillance System, COPD = Chronic obstructive pulmonary disorder, DALY = Disability adjusted life year, GBD = Global Burden of Disease, PAF = Population attributable fraction, OVOB = Overweight or obese.

[a] BRFSS data elements were available for adults age 18+ years, Hughes (2020) data elements were available for adults aged 18-69 years, and GBD (2019) and Dieleman (2020) data elements were available for adults age 20+ years. See References in main article.

© 2023 Peterson C et al. *JAMA Network Open.*

eTable 2. State-level calculation examples

a. ACE annual economic burden by type per health condition: Alabama arthritis example

DALY										
Measure name		DALY count[a]		ACE (any n) PAF[b]		DALY monetary value		DALY annual economic burden
Value		31,001	x	13.8%	x	$540,000	=	$2,308m
Reference source		GBD (2019)		2019-2020 BRFSS		US HHS ASPE (206)		Calculated
Study reporting				eTable 3				eTable 4

Medical spending									
U.S. total adjusted annual medical spending[a] (2016 USD)		Inflation adjustment 2016 USD to 2019 USD		Proportion of health expenditures by state[c]		ACE (any n) PAF[b]		Medical spending annual economic burden
$80b	x	(110.647/105.43)	x	1.4%	x	13.8%	=	$159m
Dieleman (2020)		U.S. BEA (2019)		U.S. CMS (2019)		BRFSS 2019-2020		Calculated
						eTable 3		eTable 5

b. ACE total annual economic burden (all health conditions): Alabama example

DALY annual economic burden		Medical spending annual economic burden		Total annual economic burden
$154,661m	+	$1,299m	=	$156,000m
eTable 4		eTable 5		eTable 6

c. ACE per person (affected adult) total annual and lifetime economic burden: Alabama example

Total economic burden		Number of adults with ACEs (any n)[b]		Annual economic burden per person		Lifetime economic burden per person[d]
$156,000m	÷	2,348,440	=	$66,000	=	$1,834,000
Calculated		U.S. CB; Aslam (2022)		Calculated		Calculated
eTable 6		Table 1		eTable 6		eTable 6

Abbreviations: ACE = Adverse childhood experience, BRFSS = Behavioral Risk Factors Surveillance System, DALY = Disability adjusted life year, GBD = Global Burden of Disease, PAF = Population attributable fraction, U.S. BEA = U.S. Bureau of Economic Analysis, U.S. CB = U.S. Census Bureau, U.S. CMS = U.S. Centers for Medicare & Medicaid Services. Results may appear imprecise due to rounding.
[a] Age 20+ years.
[b] Age 18+ years.
[c] All ages.
[d] Calculated as the annual economic burden per person applied to the number of years between age 18 and U.S. average life expectancy (78.7 years) discounted 3% annually to present value using PV(0.03,78.7-18,-66,000) in MS Excel.

© 2023 Peterson C et al. *JAMA Network Open.*

eTable 3. State-level ACE PAF for adult health conditions

State	Measure	BRFSS respondents with ACE data, n (weighted)	Respondents with measure by ACE n, %				Adjusted odds ratio by ACE n						ACE PAF by ACE n				
							1		2-3		4+						
			0	1	2-3	4+	aOR	95% CI	aOR	95% CI	aOR	95% CI	1	2-3	4+	Any	Any Adj.
United States	Anxiety	.	.	.	.	.	.	.	.	.	.	.	6.1	12.6	10.4	29.1	.
	Arthritis	258,397,120	22.3	22.9	25.0	28.1	1.57	(1.52-1.63)	2.12	(2.05-2.19)	3.25	(3.13-3.38)	4.2	8.8	13.3	26.3	19.1
	Asthma	259,014,944	8.4	12.3	14.8	26.0	1.58	(1.50-1.66)	1.95	(1.87-2.05)	3.65	(3.48-3.83)	5.2	10.8	27.4	43.4	34.8
	Cancer	259,196,944	8.1	6.6	6.0	6.1	1.11	(1.05-1.16)	1.08	(1.03-1.13)	1.47	(1.39-1.56)	1.5	1.3	6.1	9.0	3.4
	COPD	258,708,000	3.8	5.7	6.5	10.5	1.98	(1.86-2.11)	2.74	(2.58-2.91)	4.78	(4.49-5.09)	7.1	14.2	28.2	49.5	36.6
	Depression	258,517,328	4.7	7.1	18.3	48.3	2.12	(2.00-2.24)	6.63	(6.30-6.97)	24.92	(23.59-26.33)	3.8	20.1	54.0	77.9	76.0
	Diabetes	259,325,728	12.4	9.7	10.5	9.7	1.00	(0.95-1.05)	1.27	(1.21-1.32)	1.30	(1.24-1.37)	.	3.6	3.5	7.1	0.6
	Heart disease	258,047,280	4.2	4.0	3.8	3.6	1.28	(1.20-1.37)	1.60	(1.50-1.71)	2.06	(1.92-2.22)	3.6	7.9	10.3	21.9	8.6
	Kidney disease	258,992,080	2.7	2.8	3.3	3.3	1.43	(1.32-1.55)	1.97	(1.82-2.13)	2.10	(1.91-2.30)	5.0	12.3	12.4	29.8	18.6
	Stroke	259,142,624	3.2	2.7	2.9	3.8	1.16	(1.07-1.25)	1.45	(1.35-1.55)	1.97	(1.82-2.13)	1.9	6.1	12.7	20.7	10.4
	Violence	.	.	.	.	.	.	.	.	.	.	.	4.4	17.0	22.0	43.4	.
	Heavy drinking	237,163,952	2.7	6.2	7.5	10.6	2.07	(1.95-2.20)	2.59	(2.44-2.75)	3.93	(3.69-4.17)	9.1	16.9	27.7	53.7	.
	Illicit drug use	.	.	.	.	.	.	.	.	.	.	.	6.2	15.9	30.6	52.7	.
	OVOB	231,960,224	64.4	67.5	66.8	67.3	1.16	(1.12-1.20)	1.20	(1.16-1.23)	1.29	(1.25-1.33)	0.7	1.1	1.5	3.3	.
	Smoking	244,222,624	5.6	10.4	14.8	29.4	1.92	(1.82-2.02)	2.96	(2.83-3.09)	6.48	(6.20-6.78)	5.9	14.3	35.0	55.2	.
Alabama	Anxiety	.	.	.	.	.	.	.	.	.	.	.	6.1	12.6	10.4	29.1	.
	Arthritis	3,810,607	33.7	31.1	35.7	34.5	1.20	(1.03-1.39)	1.67	(1.44-1.95)	2.15	(1.79-2.57)	1.7	5.0	7.1	13.8	9.7
	Asthma	3,820,308	11.5	12.0	16.6	22.0	1.00	(0.81-1.22)	1.34	(1.09-1.63)	1.80	(1.46-2.22)	.	4.9	12.0	16.9	8.9
	Cancer	3,818,596	9.0	7.3	6.7	6.0	1.05	(0.85-1.31)	1.03	(0.82-1.29)	1.25	(0.97-1.60)	.	.	.	.	.
	COPD	3,809,360	7.8	9.1	10.2	14.5	1.25	(0.98-1.59)	1.43	(1.13-1.82)	2.24	(1.72-2.91)	.	5.2	14.2	19.3	8.6
	Depression	3,804,467	12.3	16.3	26.8	49.4	1.38	(1.15-1.67)	2.50	(2.09-2.98)	6.25	(5.18-7.54)	3.2	11.3	29.5	44.1	40.9
	Diabetes	3,819,732	16.5	14.4	14.3	11.7	1.04	(0.86-1.25)	1.04	(0.86-1.26)	1.21	(0.96-1.52)	.	.	.	.	.
	Heart disease	3,795,216	5.9	4.9	5.4	5.2	1.03	(0.80-1.32)	1.35	(1.03-1.78)	1.89	(1.41-2.53)	.	5.0	8.9	13.9	9.8
	Kidney disease	3,815,275	4.0	3.5	4.3	3.4	1.09	(0.80-1.50)	1.31	(0.94-1.83)	1.22	(0.83-1.78)	.	.	.	.	.
	Stroke	3,818,759	4.8	4.4	4.6	5.4	1.06	(0.81-1.39)	1.03	(0.78-1.36)	1.54	(1.10-2.15)	.	.	7.1	7.1	3.5
	Violence	.	.	.	.	.	.	.	.	.	.	.	4.4	17.0	22.0	43.4	.
	Heavy drinking	3,551,588	3.2	6.0	6.4	8.1	1.77	(1.30-2.41)	1.75	(1.28-2.41)	2.36	(1.74-3.22)	9.5	9.5	17.5	36.4	.
	Illicit drug use	.	.	.	.	.	.	.	.	.	.	.	6.2	15.9	30.6	52.7	.
	OVOB	3,538,760	70.7	72.4	74.2	69.6	1.17	(1.01-1.35)	1.33	(1.14-1.55)	1.18	(1.00-1.38)	0.8	1.5	.	2.4	.
	Smoking	3,640,304	12.0	17.9	21.5	32.1	1.42	(1.17-1.72)	1.84	(1.53-2.21)	2.60	(2.15-3.13)	4.2	7.9	15.6	27.7	.
Alaska	Anxiety	.	.	.	.	.	.	.	.	.	.	.	6.1	12.6	10.4	29.1	.
	Arthritis	547,650	18.8	21.3	24.1	23.3	2.65	(1.96-3.57)	3.66	(2.74-4.89)	5.11	(3.52-7.43)	7.3	14.5	17.2	39.0	29.7
	Asthma	548,279	8.8	14.3	15.5	23.5	2.97	(2.02-4.37)	2.50	(1.72-3.62)	5.94	(3.95-8.94)	10.0	12.3	32.5	54.8	53.9
	Cancer	549,758	6.0	7.6	3.9	4.8	2.00	(1.30-3.09)	1.14	(0.72-1.81)	1.97	(1.11-3.50)	9.2	.	9.3	18.5	15.1
	COPD	548,398	2.5	4.6	5.6	8.2	2.47	(1.45-4.21)	4.22	(2.52-7.06)	8.02	(3.95-16.28)	7.6	17.7	33.3	58.6	47.0
	Depression	547,164	3.1	9.2	21.1	45.5	13.14	(6.42-26.87)	75.93	(36.90-156.27)	636.00	(274.05-1476.00)	5.5	26.2	63.2	94.9	95.8
	Diabetes	550,366	6.8	8.2	7.5	8.5	1.75	(1.19-2.58)	2.00	(1.37-2.94)	2.77	(1.69-4.54)	6.2	10.5	14.7	31.4	19.0
	Heart disease	547,745	3.1	3.2	3.6	2.5	1.29	(0.74-2.26)	2.73	(1.52-4.92)	2.23	(1.06-4.72)	.	17.4	9.1	26.5	4.8
	Kidney disease	549,982	1.7	1.9	1.5	2.6	1.89	(0.89-4.01)	3.73	(1.92-7.25)	7.11	(3.19-15.83)	.	17.5	31.2	48.7	48.0
	Stroke	550,071	2.1	2.9	1.9	2.8	1.67	(0.88-3.17)	2.07	(1.09-3.92)	2.98	(1.41-6.32)	.	9.8	18.7	28.5	5.4
	Violence	.	.	.	.	.	.	.	.	.	.	.	4.4	17.0	22.0	43.4	.
	Heavy drinking	505,826	4.8	8.7	10.5	13.4	1.95	(1.26-3.03)	3.33	(2.17-5.10)	5.23	(3.29-8.32)	5.6	21.3	27.5	54.4	.
	Illicit drug use	.	.	.	.	.	.	.	.	.	.	.	6.2	15.9	30.6	52.7	.

© 2023 Peterson C et al. *JAMA Network Open.*

	OVOB	499,923	66.6	64.5	69.2	66.1	1.06	(0.82-1.38)	1.33	(1.04-1.69)	1.33	(0.99-1.79)	.	1.1	.	1.1	.
	Smoking	520,649	10.0	14.1	16.9	36.5	1.51	(1.03-2.22)	3.11	(2.25-4.32)	10.08	(7.10-14.32)	2.2	11.5	37.6	51.3	.
Arizona	Anxiety	.	.	.	.	.	.	.	.	.	.	.	6.1	12.6	10.4	29.1	.
	Arthritis	5,674,532	22.4	21.4	23.6	25.2	1.31	(1.11-1.54)	1.80	(1.53-2.13)	2.69	(2.23-3.23)	2.3	7.3	12.0	21.6	11.9
	Asthma	5,686,213	8.7	11.0	14.9	25.6	1.64	(1.31-2.06)	2.11	(1.72-2.58)	4.13	(3.37-5.08)	4.8	11.6	31.5	47.9	41.4
	Cancer	5,692,328	7.9	8.0	5.8	6.4	1.32	(1.04-1.67)	0.98	(0.79-1.23)	1.55	(1.16-2.05)	4.2	.	7.3	11.5	8.4
	COPD	5,675,600	4.3	5.3	6.5	9.8	1.73	(1.30-2.31)	2.36	(1.78-3.12)	4.49	(3.33-6.04)	5.5	12.4	29.0	46.8	30.5
	Depression	5,671,257	4.3	8.2	15.8	41.0	2.20	(1.67-2.90)	5.45	(4.27-6.96)	17.87	(13.91-22.94)	4.0	18.3	54.1	76.4	74.4
	Diabetes	5,695,658	12.5	10.0	11.0	10.1	0.99	(0.79-1.23)	1.36	(1.10-1.67)	1.56	(1.23-1.98)	.	4.8	6.8	11.6	4.1
	Heart disease	5,664,940	4.3	4.4	3.8	3.4	1.27	(0.94-1.72)	1.55	(1.13-2.12)	2.05	(1.46-2.89)	.	7.6	10.8	18.4	9.2
	Kidney disease	5,682,652	4.4	3.9	4.9	3.2	1.12	(0.80-1.57)	1.69	(1.25-2.27)	1.21	(0.86-1.70)	.	10.1	.	10.1	6.0
	Stroke	5,689,340	3.3	3.0	3.2	3.6	1.11	(0.75-1.63)	1.34	(0.95-1.90)	1.59	(1.10-2.29)	.	.	9.8	9.8	5.2
	Violence	.	.	.	.	.	.	.	.	.	.	.	4.4	17.0	22.0	43.4	.
	Heavy drinking	5,158,369	2.6	5.4	8.3	8.0	1.73	(1.26-2.39)	3.08	(2.35-4.03)	3.00	(2.24-4.01)	5.9	22.9	23.0	51.7	.
	Illicit drug use	.	.	.	.	.	.	.	.	.	.	.	6.2	15.9	30.6	52.7	.
	OVOB	5,028,827	65.4	68.0	65.5	65.7	1.10	(0.95-1.28)	1.10	(0.95-1.26)	1.12	(0.97-1.31)	.	.	.	.	.
	Smoking	5,315,009	6.4	8.7	13.6	25.6	1.39	(1.06-1.81)	2.33	(1.85-2.92)	4.71	(3.78-5.88)	2.7	11.9	34.2	48.9	.
Arkansas	Anxiety	.	.	.	.	.	.	.	.	.	.	.	6.1	12.6	10.4	29.1	.
	Arthritis	2,318,510	25.5	26.6	31.9	41.8	2.21	(1.82-2.68)	3.03	(2.46-3.72)	5.63	(4.34-7.30)	6.8	9.0	17.2	33.0	23.1
	Asthma	2,321,942	6.4	9.3	16.7	28.4	2.02	(1.51-2.70)	3.06	(2.29-4.09)	7.17	(5.28-9.74)	7.6	13.4	37.5	58.5	52.9
	Cancer	2,322,611	9.3	7.1	5.8	8.0	1.12	(0.85-1.48)	1.01	(0.75-1.36)	1.74	(1.25-2.42)	.	.	8.7	8.7	.
	COPD	2,317,133	4.7	7.1	10.8	21.9	3.36	(2.54-4.43)	5.36	(4.02-7.14)	11.30	(8.13-15.70)	10.6	16.2	38.9	65.7	59.0
	Depression	2,313,558	3.7	3.0	36.4	72.2	9.97	(6.51-15.27)	216.29	(136.48-342.76)	1501.31	(881.07-2558.18)	3.2	28.4	65.3	96.9	97.0
	Diabetes	2,325,151	15.3	11.8	13.6	11.8	1.09	(0.87-1.37)	1.51	(1.20-1.90)	1.58	(1.20-2.10)	.	4.7	6.0	10.7	3.7
	Heart disease	2,306,326	6.5	6.5	6.9	7.4	1.97	(1.50-2.59)	2.17	(1.62-2.91)	3.27	(2.27-4.72)	8.8	8.5	15.4	32.7	20.6
	Kidney disease	2,322,868	3.3	3.3	3.9	3.9	1.73	(1.19-2.53)	2.03	(1.40-2.94)	2.27	(1.41-3.64)	8.2	9.1	13.5	30.8	15.9
	Stroke	2,322,294	3.8	3.7	4.4	6.9	1.55	(1.09-2.21)	1.86	(1.30-2.67)	3.12	(2.04-4.77)	5.2	6.8	22.0	34.0	13.3
	Violence	.	.	.	.	.	.	.	.	.	.	.	4.4	17.0	22.0	43.4	.
	Heavy drinking	2,144,765	1.1	5.3	9.6	11.5	5.79	(3.67-9.14)	9.01	(5.71-14.21)	12.46	(7.75-20.05)	19.8	25.4	35.7	80.9	.
	Illicit drug use	.	.	.	.	.	.	.	.	.	.	.	6.2	15.9	30.6	52.7	.
	OVOB	2,104,913	67.3	69.3	69.1	71.1	1.29	(1.07-1.56)	1.20	(0.98-1.46)	1.67	(1.33-2.08)	1.4	.	3.2	4.7	.
	Smoking	2,200,864	5.2	13.2	26.1	47.3	4.24	(3.14-5.72)	7.68	(5.75-10.27)	18.58	(13.67-25.26)	12.0	19.0	44.6	75.6	.
California	Anxiety	.	.	.	.	.	.	.	.	.	.	.	6.1	12.6	10.4	29.1	.
	Arthritis	30,705,952	16.8	23.1	20.8	22.5	2.13	(1.74-2.60)	2.71	(2.26-3.25)	4.57	(3.76-5.57)	5.5	13.0	20.5	39.0	31.7
	Asthma	30,746,348	9.1	14.6	14.6	25.5	1.62	(1.27-2.06)	1.67	(1.36-2.06)	3.89	(3.16-4.78)	3.7	8.8	31.7	44.2	34.2
	Cancer	30,776,892	6.3	7.0	5.2	5.6	1.10	(0.83-1.46)	1.04	(0.82-1.31)	1.71	(1.30-2.27)	.	.	10.1	10.1	7.8
	COPD	30,704,906	2.5	6.0	4.9	6.7	3.22	(2.04-5.08)	4.66	(3.11-6.96)	7.98	(5.24-12.15)	8.9	22.6	36.5	68.0	57.5
	Depression	30,713,574	4.4	6.0	11.3	37.5	1.82	(1.23-2.69)	5.76	(4.17-7.94)	32.85	(23.42-46.07)	1.6	16.1	66.7	84.4	82.8
	Diabetes	30,774,166	11.6	9.1	9.3	8.8	0.97	(0.76-1.23)	1.27	(1.03-1.55)	1.23	(0.98-1.54)	.	4.0	.	4.0	1.1
	Heart disease	30,707,042	2.8	3.5	2.8	2.6	1.52	(1.01-2.30)	2.11	(1.43-3.11)	2.63	(1.73-4.00)	4.7	15.0	15.6	35.3	9.7
	Kidney disease	30,746,822	2.4	2.3	3.2	3.2	1.49	(0.94-2.36)	2.61	(1.81-3.77)	3.09	(1.96-4.88)	.	18.3	21.1	39.4	31.9
	Stroke	30,753,454	2.9	2.1	2.0	3.0	1.09	(0.68-1.76)	1.30	(0.89-1.90)	1.90	(1.23-2.95)	.	.	14.4	14.4	11.3
	Violence	.	.	.	.	.	.	.	.	.	.	.	4.4	17.0	22.0	43.4	.
	Heavy drinking	28,158,584	1.9	4.4	6.0	12.4	2.16	(1.48-3.15)	3.24	(2.31-4.56)	8.29	(6.03-11.38)	4.4	18.8	47.2	70.4	.
	Illicit drug use	.	.	.	.	.	.	.	.	.	.	.	6.2	15.9	30.6	52.7	.
	OVOB	27,761,998	61.2	65.7	64.2	67.0	1.32	(1.13-1.54)	1.20	(1.05-1.36)	1.35	(1.18-1.55)	1.1	1.5	2.2	4.9	.
	Smoking	28,547,836	4.4	7.1	7.3	19.4	2.03	(1.50-2.74)	1.70	(1.29-2.24)	6.27	(4.92-8.00)	5.0	6.9	44.9	56.8	.

© 2023 Peterson C et al. *JAMA Network Open.*

Colorado	Anxiety	.	.	.	.	.	.	.	.	.	.	.	6.1	12.6	10.4	29.1	.
	Arthritis	4,536,657	21.8	20.1	20.9	25.3	2.12	(1.81-2.48)	2.80	(2.44-3.22)	6.08	(5.13-7.20)	5.6	13.3	23.0	41.9	35.7
	Asthma	4,540,266	9.9	11.8	12.4	25.0	2.22	(1.80-2.73)	1.89	(1.57-2.27)	5.80	(4.76-7.07)	6.7	10.6	38.5	55.8	48.2
	Cancer	4,544,009	8.9	6.8	4.8	5.7	0.97	(0.78-1.20)	1.01	(0.84-1.22)	1.81	(1.45-2.24)	.	.	11.5	11.5	7.6
	COPD	4,538,906	2.9	4.8	4.2	6.7	4.64	(3.41-6.30)	5.25	(3.94-6.97)	9.35	(6.58-13.30)	12.4	21.5	36.4	70.3	62.0
	Depression	4,537,827	3.3	2.7	16.6	44.4	5.06	(2.84-9.01)	77.06	(46.25-128.39)	480.84	(278.78-829.37)	1.1	27.2	69.3	97.7	97.7
	Diabetes	4,547,666	7.1	9.2	6.8	6.7	1.19	(0.96-1.48)	1.63	(1.35-1.96)	1.78	(1.40-2.26)	.	8.6	9.7	18.3	4.8
	Heart disease	4,533,893	3.0	3.6	2.1	2.6	1.63	(1.20-2.22)	1.86	(1.41-2.46)	4.50	(3.25-6.22)	5.7	10.1	22.5	38.3	19.7
	Kidney disease	4,546,992	1.7	2.0	1.7	2.6	1.54	(1.02-2.33)	2.23	(1.57-3.17)	4.40	(2.81-6.92)	4.3	14.1	28.5	46.9	28.8
	Stroke	4,545,951	2.2	2.1	1.8	2.8	1.82	(1.24-2.65)	1.77	(1.24-2.54)	3.52	(2.27-5.47)	6.7	8.8	27.6	43.1	31.5
	Violence	.	.	.	.	.	.	.	.	.	.	.	4.4	17.0	22.0	43.4	.
	Heavy drinking	4,170,773	2.4	4.1	8.6	11.3	2.78	(2.05-3.76)	4.54	(3.51-5.89)	8.36	(6.32-11.06)	6.5	26.5	41.3	74.3	.
	Illicit drug use	.	.	.	.	.	.	.	.	.	.	.	6.2	15.9	30.6	52.7	.
	OVOB	4,156,280	57.4	66.2	60.1	57.0	1.38	(1.23-1.56)	1.35	(1.21-1.50)	1.43	(1.27-1.61)	1.7	2.6	2.9	7.1	.
	Smoking	4,243,536	3.9	6.9	10.4	28.6	2.53	(1.93-3.32)	4.15	(3.27-5.28)	15.66	(12.29-19.95)	4.8	15.5	53.8	74.1	.
Connecticut	Anxiety	.	.	.	.	.	.	.	.	.	.	.	6.1	12.6	10.4	29.1	.
	Arthritis	2,827,593	20.3	24.1	24.0	27.1	2.41	(2.08-2.80)	2.57	(2.19-3.02)	6.93	(5.45-8.82)	10.0	10.4	11.7	32.1	28.0
	Asthma	2,835,438	8.5	14.5	18.3	33.4	2.20	(1.78-2.73)	2.95	(2.41-3.62)	7.00	(5.48-8.95)	11.2	17.6	23.5	52.2	49.5
	Cancer	2,834,891	8.7	7.2	6.8	5.6	1.18	(0.97-1.43)	1.33	(1.06-1.67)	1.50	(1.05-2.13)	.	4.6	2.8	7.4	.
	COPD	2,829,936	2.6	6.2	5.5	10.0	3.95	(2.88-5.41)	4.19	(3.04-5.79)	13.73	(8.84-21.34)	16.7	16.4	27.6	60.7	53.7
	Depression	2,827,301	2.6	5.5	27.4	53.4	7.80	(4.88-12.48)	95.62	(59.75-153.04)	616.42	(362.47-1048.29)	6.2	39.1	49.2	94.5	95.0
	Diabetes	2,839,139	10.9	8.1	8.7	9.4	1.24	(1.01-1.52)	1.46	(1.18-1.81)	1.72	(1.23-2.40)	2.7	5.6	4.0	12.2	0.6
	Heart disease	2,823,732	3.4	3.8	4.0	2.6	1.86	(1.40-2.48)	2.06	(1.53-2.77)	2.52	(1.73-3.65)	10.9	10.7	6.3	27.9	13.6
	Kidney disease	2,834,206	1.9	2.4	2.6	2.4	2.11	(1.45-3.07)	2.44	(1.64-3.64)	2.24	(1.26-3.99)	13.1	15.8	7.7	36.6	29.6
	Stroke	2,835,657	2.2	2.6	2.0	3.2	2.49	(1.68-3.69)	2.09	(1.40-3.10)	3.07	(1.79-5.28)	14.6	10.7	11.3	36.5	23.0
	Violence	.	.	.	.	.	.	.	.	.	.	.	4.4	17.0	22.0	43.4	.
	Heavy drinking	2,601,648	2.5	8.1	8.0	9.5	3.38	(2.57-4.46)	3.17	(2.39-4.22)	5.47	(3.96-7.56)	20.7	19.4	18.8	58.9	.
	Illicit drug use	.	.	.	.	.	.	.	.	.	.	.	6.2	15.9	30.6	52.7	.
	OVOB	2,512,885	63.6	64.9	65.9	67.8	1.31	(1.15-1.51)	1.49	(1.29-1.71)	1.66	(1.35-2.02)	1.6	2.6	1.7	6.0	.
	Smoking	2,672,830	3.7	7.5	15.5	33.9	2.73	(2.10-3.55)	7.76	(6.03-9.98)	25.22	(19.11-33.27)	7.8	27.1	37.4	72.3	.
Delaware	Anxiety	.	.	.	.	.	.	.	.	.	.	.	6.1	12.6	10.4	29.1	.
	Arthritis	775,043	25.0	26.1	30.5	30.4	1.51	(1.21-1.89)	2.17	(1.74-2.71)	2.56	(1.96-3.34)	3.7	9.7	9.4	22.7	17.2
	Asthma	775,698	9.7	15.1	14.9	27.0	1.77	(1.31-2.39)	1.60	(1.18-2.16)	2.67	(1.97-3.62)	7.1	7.4	19.2	33.7	21.3
	Cancer	775,371	8.2	8.6	6.7	7.8	1.39	(1.02-1.88)	1.21	(0.88-1.67)	1.95	(1.35-2.80)	4.9	.	9.4	14.3	10.4
	COPD	773,816	4.6	7.5	7.8	12.5	2.11	(1.45-3.06)	2.57	(1.74-3.78)	4.03	(2.70-6.02)	8.3	13.8	21.9	44.0	33.3
	Depression	773,543	5.5	7.8	17.9	48.9	2.23	(1.51-3.31)	5.42	(3.78-7.76)	23.82	(16.29-34.84)	4.5	18.2	53.1	75.8	74.7
	Diabetes	776,176	15.5	12.1	13.1	9.5	0.95	(0.70-1.28)	1.21	(0.91-1.60)	1.05	(0.74-1.50)	.	.	.	.	.
	Heart disease	772,230	4.9	4.6	5.5	4.2	1.16	(0.77-1.74)	1.99	(1.32-2.99)	2.24	(1.42-3.56)	.	12.2	9.4	21.6	6.0
	Kidney disease	774,315	3.2	4.8	5.1	4.0	2.18	(1.36-3.49)	2.70	(1.74-4.20)	2.53	(1.55-4.13)	11.1	17.9	11.8	40.7	38.5
	Stroke	775,959	3.8	3.1	4.4	3.6	1.19	(0.74-1.92)	1.92	(1.21-3.06)	1.94	(1.17-3.23)	.	12.7	10.3	23.0	14.2
	Violence	.	.	.	.	.	.	.	.	.	.	.	4.4	17.0	22.0	43.4	.
	Heavy drinking	708,354	3.6	5.8	8.4	10.5	1.77	(1.07-2.93)	2.36	(1.54-3.63)	3.40	(2.13-5.41)	7.0	15.8	23.2	46.0	.
	Illicit drug use	.	.	.	.	.	.	.	.	.	.	.	6.2	15.9	30.6	52.7	.
	OVOB	684,613	67.5	70.2	70.6	67.5	1.27	(1.04-1.56)	1.39	(1.12-1.72)	1.38	(1.10-1.74)	1.3	2.2	2.0	5.5	.
	Smoking	724,188	6.0	9.9	22.1	29.2	2.11	(1.53-2.90)	4.31	(3.21-5.80)	5.93	(4.42-7.96)	6.2	21.4	28.8	56.4	.
DC	Anxiety	.	.	.	.	.	.	.	.	.	.	.	6.1	12.6	10.4	29.1	.
	Arthritis	576,909	19.2	12.5	14.9	21.4	1.29	(0.99-1.70)	1.59	(1.25-2.04)	3.37	(2.50-4.54)	.	6.6	13.4	20.0	16.0
	Asthma	578,842	14.6	13.8	17.9	23.8	1.11	(0.82-1.51)	1.33	(1.00-1.76)	1.86	(1.38-2.51)	.	5.7	10.4	16.0	10.4

© 2023 Peterson C et al. *JAMA Network Open.*

	Cancer	579,161	6.8	5.0	3.9	4.1	1.25	(0.90-1.74)	1.13	(0.81-1.56)	1.51	(0.99-2.32)	.	.	.	.	.
	COPD	577,481	4.2	2.8	4.1	7.0	1.06	(0.60-1.89)	1.41	(0.87-2.28)	2.62	(1.55-4.44)	.	.	18.7	18.7	17.5
	Depression	575,927	8.4	10.0	20.3	54.6	1.87	(1.28-2.73)	3.80	(2.73-5.28)	20.89	(14.54-30.01)	5.2	17.7	44.5	67.4	65.8
	Diabetes	578,983	9.8	6.7	7.7	8.3	1.36	(0.95-1.95)	1.42	(1.03-1.95)	1.65	(1.11-2.46)	.	5.6	5.6	11.2	6.2
	Heart disease	575,966	3.3	2.6	2.4	2.6	1.72	(0.95-3.12)	1.80	(1.03-3.15)	1.88	(1.03-3.44)	.	10.4	7.1	17.6	10.3
	Kidney disease	578,449	3.3	1.9	1.9	1.7	0.90	(0.49-1.66)	0.89	(0.46-1.70)	0.63	(0.32-1.24)	.	.	.	.	.
	Stroke	578,631	3.5	2.5	3.0	3.2	1.31	(0.79-2.17)	1.34	(0.81-2.22)	1.19	(0.66-2.13)	.	.	.	.	.
	Violence	.	.	.	.	.	.	.	.	.	.	.	4.4	17.0	22.0	43.4	.
	Heavy drinking	519,372	5.7	8.2	7.3	14.7	1.52	(1.04-2.24)	1.08	(0.74-1.58)	2.53	(1.75-3.68)	7.4	.	17.7	25.1	.
	Illicit drug use	.	.	.	.	.	.	.	.	.	.	.	6.2	15.9	30.6	52.7	.
	OVOB	520,143	57.9	54.4	54.8	59.5	1.10	(0.89-1.36)	1.04	(0.85-1.27)	1.06	(0.85-1.34)	.	.	.	.	.
	Smoking	535,721	7.2	7.6	14.9	19.8	1.34	(0.89-2.02)	2.70	(1.95-3.74)	3.19	(2.24-4.54)	.	17.8	17.3	35.1	.
Florida	Anxiety	.	.	.	.	.	.	.	.	.	.	.	6.1	12.6	10.4	29.1	.
	Arthritis	17,439,336	24.1	24.5	26.5	28.7	1.30	(1.10-1.54)	1.69	(1.43-2.00)	2.64	(2.17-3.21)	2.9	5.7	10.5	19.2	12.3
	Asthma	17,480,340	8.4	11.3	13.6	20.4	1.49	(1.17-1.91)	1.83	(1.44-2.31)	2.66	(2.12-3.32)	5.8	9.4	19.9	35.0	29.0
	Cancer	17,462,906	8.8	7.4	7.0	6.3	1.03	(0.83-1.27)	1.00	(0.79-1.27)	1.40	(1.07-1.81)	.	.	5.0	5.0	2.7
	COPD	17,465,224	5.4	7.6	9.5	11.2	1.82	(1.43-2.33)	2.25	(1.72-2.95)	3.08	(2.37-4.00)	8.4	11.7	18.2	38.4	24.6
	Depression	17,426,162	6.8	9.7	18.7	39.7	1.85	(1.45-2.36)	3.77	(2.98-4.78)	9.64	(7.74-12.00)	5.3	15.5	41.5	62.3	59.7
	Diabetes	17,472,064	14.1	11.2	11.7	9.3	0.99	(0.81-1.22)	1.20	(0.97-1.48)	1.09	(0.86-1.37)	.	.	.	.	.
	Heart disease	17,402,834	5.0	5.2	5.0	4.9	1.64	(1.23-2.18)	1.76	(1.33-2.32)	2.29	(1.70-3.08)	8.6	8.8	11.7	29.1	21.6
	Kidney disease	17,467,622	3.8	3.7	4.5	3.0	1.14	(0.83-1.56)	1.56	(1.12-2.17)	1.25	(0.85-1.84)	.	8.4	.	8.4	2.5
	Stroke	17,472,296	3.9	3.5	3.2	4.3	1.20	(0.88-1.64)	1.22	(0.87-1.69)	2.01	(1.40-2.89)	.	.	12.4	12.4	8.6
	Violence	.	.	.	.	.	.	.	.	.	.	.	4.4	17.0	22.0	43.4	.
	Heavy drinking	15,611,576	3.9	6.4	9.1	11.1	1.63	(1.20-2.22)	2.26	(1.75-2.92)	2.98	(2.28-3.89)	7.1	13.2	22.3	42.6	.
	Illicit drug use	.	.	.	.	.	.	.	.	.	.	.	6.2	15.9	30.6	52.7	.
	OVOB	15,173,157	64.7	64.5	65.6	62.5	1.02	(0.87-1.19)	1.07	(0.91-1.25)	1.03	(0.88-1.22)	.	.	.	.	.
	Smoking	16,038,803	7.7	14.4	17.0	23.9	1.85	(1.46-2.35)	2.05	(1.63-2.58)	3.00	(2.42-3.72)	7.7	9.7	19.6	37.0	.
Georgia	Anxiety	.	.	.	.	.	.	.	.	.	.	.	6.1	12.6	10.4	29.1	.
	Arthritis	8,162,652	22.3	21.4	24.8	31.2	1.53	(1.27-1.83)	2.02	(1.68-2.43)	3.33	(2.69-4.13)	3.9	8.0	13.2	25.1	18.5
	Asthma	8,198,753	7.9	11.3	14.0	24.0	1.38	(1.05-1.82)	2.03	(1.59-2.60)	3.47	(2.71-4.43)	4.0	11.1	24.9	40.0	31.4
	Cancer	8,191,959	7.4	6.2	5.1	6.8	1.33	(1.04-1.71)	1.06	(0.81-1.37)	1.53	(1.13-2.06)	4.5	.	6.8	11.3	5.6
	COPD	8,162,379	4.7	5.0	6.1	14.1	1.62	(1.18-2.23)	2.14	(1.55-2.96)	4.40	(3.20-6.04)	4.9	9.7	27.8	42.4	31.5
	Depression	8,164,580	4.5	8.3	19.4	48.5	2.13	(1.58-2.87)	5.55	(4.26-7.24)	17.84	(13.51-23.57)	4.6	18.3	50.8	73.7	70.4
	Diabetes	8,196,464	13.5	9.7	12.7	10.3	0.89	(0.72-1.11)	1.23	(0.99-1.52)	1.09	(0.85-1.39)	.	.	.	.	.
	Heart disease	8,143,571	4.9	4.3	4.2	4.1	1.24	(0.89-1.75)	1.20	(0.86-1.66)	1.44	(0.96-2.15)	.	.	.	.	.
	Kidney disease	8,197,023	3.3	3.5	4.4	3.4	1.38	(0.96-1.98)	1.89	(1.30-2.73)	1.55	(1.06-2.27)	.	11.8	6.8	18.6	9.5
	Stroke	8,196,192	4.0	2.9	3.5	4.0	1.12	(0.78-1.62)	1.22	(0.83-1.77)	1.29	(0.89-1.87)	.	.	.	.	.
	Violence	.	.	.	.	.	.	.	.	.	.	.	4.4	17.0	22.0	43.4	.
	Heavy drinking	7,377,227	2.8	5.2	6.7	10.4	1.83	(1.28-2.61)	2.80	(2.00-3.92)	4.70	(3.35-6.58)	6.9	17.4	30.8	55.1	.
	Illicit drug use	.	.	.	.	.	.	.	.	.	.	.	6.2	15.9	30.6	52.7	.
	OVOB	7,210,964	66.5	65.8	69.4	67.3	1.17	(0.99-1.40)	1.27	(1.07-1.51)	1.32	(1.11-1.58)	.	1.5	1.7	3.2	.
	Smoking	7,570,393	7.7	11.1	17.7	34.7	1.75	(1.34-2.28)	2.51	(1.97-3.20)	6.26	(4.93-7.94)	5.0	12.0	32.7	49.7	.
Hawaii	Anxiety	.	.	.	.	.	.	.	.	.	.	.	6.1	12.6	10.4	29.1	.
	Arthritis	1,106,541	19.6	21.5	21.2	22.9	1.41	(1.20-1.65)	1.83	(1.55-2.15)	2.44	(2.02-2.94)	4.5	7.4	9.8	21.6	16.4
	Asthma	1,111,238	10.8	15.1	16.9	23.0	1.61	(1.33-1.95)	1.94	(1.61-2.35)	2.59	(2.12-3.15)	6.9	10.6	16.4	33.9	24.5
	Cancer	1,111,432	7.2	6.0	5.2	5.2	1.02	(0.80-1.30)	1.13	(0.87-1.48)	1.32	(0.94-1.83)	.	.	.	.	.
	COPD	1,109,573	2.4	3.7	4.4	7.3	1.71	(1.20-2.44)	2.08	(1.48-2.92)	3.74	(2.65-5.26)	7.7	10.3	24.6	42.6	31.7
	Depression	1,107,909	4.6	7.2	12.4	35.9	1.89	(1.43-2.51)	3.79	(2.98-4.82)	14.39	(11.30-18.33)	5.1	14.8	48.7	68.7	67.2

© 2023 Peterson C et al. *JAMA Network Open.*

	Diabetes	1,110,228	10.8	9.9	10.7	12.1	1.08	(0.89-1.33)	1.44	(1.16-1.77)	2.00	(1.57-2.55)	.	5.3	9.3	14.5	11.7
	Heart disease	1,107,758	2.7	3.0	2.5	3.4	1.30	(0.91-1.85)	1.46	(0.99-2.15)	2.25	(1.51-3.35)	.	.	11.5	11.5	3.2
	Kidney disease	1,110,500	2.4	2.7	3.3	3.6	1.33	(0.89-1.99)	2.40	(1.63-3.54)	2.04	(1.34-3.10)	.	15.5	11.0	26.5	17.2
	Stroke	1,111,206	3.3	2.5	2.3	3.2	0.95	(0.68-1.33)	1.00	(0.70-1.42)	1.37	(0.95-1.96)	.	.	.	.	.
	Violence	.	.	.	.	.	.	.	.	.	.	.	4.4	17.0	22.0	43.4	.
	Heavy drinking	1,055,238	3.5	7.2	10.0	15.7	1.72	(1.29-2.29)	2.61	(1.97-3.47)	4.08	(3.02-5.51)	7.1	16.2	26.4	49.7	.
	Illicit drug use	.	.	.	.	.	.	.	.	.	.	.	6.2	15.9	30.6	52.7	.
	OVOB	1,054,124	52.6	57.8	61.9	65.7	1.17	(1.03-1.32)	1.38	(1.21-1.57)	1.55	(1.33-1.81)	1.3	2.5	2.9	6.7	.
	Smoking	1,067,091	5.6	9.8	12.7	26.2	1.60	(1.25-2.05)	2.20	(1.75-2.77)	4.84	(3.85-6.08)	5.6	11.5	30.5	47.6	.
Idaho	Anxiety	.	.	.	.	.	.	.	.	.	.	.	6.1	12.6	10.4	29.1	.
	Arthritis	1,349,891	20.8	22.9	25.1	28.2	1.68	(1.37-2.07)	2.22	(1.81-2.73)	3.70	(2.92-4.68)	5.2	8.5	16.9	30.6	23.4
	Asthma	1,349,332	8.9	11.5	12.2	22.3	1.48	(1.12-1.97)	1.65	(1.25-2.17)	2.99	(2.28-3.92)	5.0	7.7	26.1	38.8	30.2
	Cancer	1,353,406	8.0	6.3	5.1	6.0	0.92	(0.68-1.23)	0.96	(0.71-1.30)	1.48	(1.06-2.06)	.	.	6.5	6.5	5.3
	COPD	1,351,149	3.5	5.1	5.4	8.4	1.52	(1.05-2.22)	2.44	(1.69-3.51)	4.20	(2.88-6.12)	4.9	11.7	28.9	45.5	29.8
	Depression	1,349,847	5.4	10.0	19.3	50.6	1.83	(1.33-2.51)	5.28	(3.93-7.09)	20.78	(15.19-28.41)	3.2	16.0	56.1	75.4	73.0
	Diabetes	1,356,421	10.3	8.9	8.1	9.6	1.02	(0.77-1.36)	1.47	(1.12-1.92)	1.86	(1.34-2.58)	.	5.6	10.8	16.4	3.8
	Heart disease	1,350,869	4.0	3.8	3.9	4.2	1.05	(0.69-1.60)	1.61	(1.06-2.45)	2.06	(1.30-3.27)	.	7.4	13.0	20.5	5.2
	Kidney disease	1,352,806	2.5	3.0	3.5	3.6	1.61	(1.02-2.56)	2.45	(1.58-3.79)	2.76	(1.77-4.30)	6.6	14.5	18.4	39.6	26.0
	Stroke	1,355,383	2.2	3.4	3.5	3.6	1.60	(0.99-2.60)	2.38	(1.51-3.75)	2.13	(1.24-3.65)	.	14.0	15.2	29.3	14.1
	Violence	.	.	.	.	.	.	.	.	.	.	.	4.4	17.0	22.0	43.4	.
	Heavy drinking	1,264,921	3.3	5.9	6.6	13.3	1.80	(1.20-2.70)	2.21	(1.51-3.24)	4.79	(3.24-7.08)	6.7	11.5	35.8	54.0	.
	Illicit drug use	.	.	.	.	.	.	.	.	.	.	.	6.2	15.9	30.6	52.7	.
	OVOB	1,218,011	62.6	65.9	67.2	66.8	1.15	(0.97-1.37)	1.26	(1.05-1.51)	1.58	(1.30-1.92)	.	1.5	3.7	5.2	.
	Smoking	1,314,466	5.8	9.1	14.0	30.8	1.40	(1.06-1.86)	2.55	(1.95-3.34)	6.37	(4.94-8.22)	2.5	11.0	39.4	52.8	.
Illinois	Anxiety	.	.	.	.	.	.	.	.	.	.	.	6.1	12.6	10.4	29.1	.
	Arthritis	9,813,954	23.0	22.2	25.9	27.9	1.65	(1.35-2.01)	2.92	(2.34-3.65)	5.81	(4.43-7.64)	7.3	12.5	16.1	35.8	29.0
	Asthma	9,827,890	6.5	11.1	15.9	22.7	1.98	(1.40-2.80)	3.05	(2.15-4.33)	6.29	(4.33-9.14)	10.7	16.8	31.5	59.0	50.7
	Cancer	9,828,795	8.3	6.7	5.2	4.4	1.08	(0.84-1.38)	1.18	(0.88-1.59)	1.33	(0.88-2.01)	.	.	.	.	.
	COPD	9,817,840	2.1	4.0	7.4	12.7	3.46	(2.24-5.36)	10.38	(6.65-16.22)	19.95	(11.86-33.57)	13.0	29.5	36.0	78.5	68.5
	Depression	9,801,863	3.5	2.1	23.3	54.4	1.40	(0.77-2.52)	45.77	(25.68-81.58)	322.86	(164.50-633.68)	.	35.0	57.5	92.5	92.4
	Diabetes	9,839,470	12.8	9.4	11.5	8.7	1.00	(0.78-1.27)	1.45	(1.12-1.87)	1.55	(1.11-2.16)	.	5.2	4.9	10.1	.
	Heart disease	9,786,452	3.3	4.3	3.6	2.2	1.66	(1.18-2.34)	2.22	(1.48-3.34)	2.14	(1.25-3.68)	12.2	12.9	6.2	31.2	20.1
	Kidney disease	9,824,046	2.1	2.6	3.5	2.3	2.67	(1.68-4.24)	4.56	(2.78-7.47)	4.47	(2.47-8.09)	18.2	22.9	16.7	57.8	51.9
	Stroke	9,821,451	3.2	2.4	3.1	3.4	1.14	(0.71-1.80)	1.56	(0.97-2.50)	2.15	(1.22-3.78)	.	.	13.0	13.0	7.6
	Violence	.	.	.	.	.	.	.	.	.	.	.	4.4	17.0	22.0	43.4	.
	Heavy drinking	9,178,328	2.6	6.2	8.3	12.0	2.37	(1.60-3.52)	3.87	(2.63-5.70)	4.45	(2.90-6.85)	15.0	25.2	22.1	62.4	.
	Illicit drug use	.	.	.	.	.	.	.	.	.	.	.	6.2	15.9	30.6	52.7	.
	OVOB	9,000,539	62.6	69.5	67.3	68.2	1.42	(1.20-1.69)	1.26	(1.05-1.51)	1.57	(1.27-1.94)	3.1	1.5	2.4	7.0	.
	Smoking	9,447,440	4.1	7.0	17.8	35.0	2.14	(1.47-3.11)	7.09	(5.05-9.96)	21.11	(14.62-30.50)	7.3	23.7	44.7	75.8	.
Indiana	Anxiety	.	.	.	.	.	.	.	.	.	.	.	6.1	12.6	10.4	29.1	.
	Arthritis	5,152,792	24.7	22.9	27.9	33.0	1.56	(1.36-1.80)	2.08	(1.84-2.37)	3.29	(2.82-3.82)	3.6	8.4	13.9	25.9	19.4
	Asthma	5,172,248	7.7	10.5	16.1	26.3	1.54	(1.25-1.89)	2.42	(2.02-2.90)	4.56	(3.78-5.49)	4.1	13.4	32.4	49.9	41.3
	Cancer	5,171,253	8.6	6.3	6.1	6.7	1.07	(0.88-1.30)	0.99	(0.82-1.20)	1.44	(1.16-1.77)	.	.	5.8	5.8	2.2
	COPD	5,165,245	5.0	6.1	9.2	16.1	1.74	(1.39-2.19)	2.54	(2.07-3.12)	4.79	(3.84-5.99)	4.6	12.7	29.9	47.2	29.0
	Depression	5,164,559	5.5	9.2	27.8	52.9	2.34	(1.87-2.94)	7.15	(5.89-8.68)	23.84	(19.37-29.35)	4.1	21.0	52.0	77.2	74.4
	Diabetes	5,177,084	12.9	10.8	12.9	11.7	1.17	(0.98-1.39)	1.41	(1.21-1.64)	1.40	(1.16-1.68)	.	5.2	4.7	10.0	1.9
	Heart disease	5,148,119	4.8	3.8	4.5	5.2	1.35	(1.07-1.71)	1.61	(1.28-2.02)	2.91	(2.28-3.73)	3.4	7.3	16.8	27.5	16.3
	Kidney disease	5,169,204	3.1	2.9	4.0	3.6	1.50	(1.13-1.99)	1.86	(1.42-2.44)	2.22	(1.62-3.04)	4.9	10.8	14.1	29.8	15.2

© 2023 Peterson C et al. *JAMA Network Open.*

	Stroke	5,173,602	3.5	3.1	3.5	5.0	1.34	(1.02-1.76)	1.38	(1.05-1.80)	2.12	(1.62-2.76)	3.4	5.2	14.9	23.5	15.3
	Violence	.	.	.	.	.	.	.	.	.	.	.	4.4	17.0	22.0	43.4	.
	Heavy drinking	4,746,580	2.8	6.7	7.5	8.9	2.17	(1.65-2.86)	2.55	(1.97-3.30)	3.22	(2.45-4.21)	10.4	16.6	23.3	50.3	.
	Illicit drug use	.	.	.	.	.	.	.	.	.	.	.	6.2	15.9	30.6	52.7	.
	OVOB	4,634,077	67.3	69.3	70.6	70.4	1.18	(1.04-1.35)	1.20	(1.06-1.35)	1.38	(1.20-1.58)	0.8	1.0	2.0	3.8	.
	Smoking	4,890,733	7.7	13.8	21.7	40.7	1.89	(1.56-2.28)	3.07	(2.60-3.63)	6.92	(5.84-8.21)	5.1	14.0	35.4	54.4	.
Iowa	Anxiety	.	.	.	.	.	.	.	.	.	.	.	6.1	12.6	10.4	29.1	.
	Arthritis	2,430,292	23.6	24.5	25.3	29.6	1.45	(1.29-1.62)	1.74	(1.54-1.96)	2.75	(2.39-3.16)	4.3	6.4	9.5	20.3	15.9
	Asthma	2,429,790	7.7	8.7	14.1	22.7	1.18	(0.99-1.41)	1.68	(1.43-1.98)	2.75	(2.33-3.25)	.	9.0	20.0	29.0	20.9
	Cancer	2,434,685	7.8	6.9	5.6	5.8	1.15	(0.97-1.36)	1.03	(0.86-1.23)	1.40	(1.13-1.74)	.	.	4.2	4.2	1.9
	COPD	2,428,207	4.3	5.0	7.7	10.5	1.32	(1.06-1.64)	2.11	(1.72-2.60)	2.71	(2.14-3.43)	3.7	12.4	15.8	31.9	21.1
	Depression	2,429,773	6.7	11.4	20.0	40.1	1.79	(1.52-2.12)	2.99	(2.56-3.50)	6.78	(5.78-7.95)	5.8	15.2	32.7	53.7	49.7
	Diabetes	2,436,916	10.7	9.7	10.7	9.3	1.06	(0.91-1.23)	1.26	(1.08-1.48)	1.28	(1.05-1.54)	.	3.8	2.8	6.5	2.0
	Heart disease	2,423,026	4.3	4.0	4.0	3.8	1.17	(0.94-1.45)	1.22	(0.96-1.54)	1.72	(1.31-2.26)	.	.	6.8	6.8	1.9
	Kidney disease	2,432,260	2.4	2.1	2.4	2.4	1.05	(0.78-1.42)	1.31	(0.98-1.76)	1.45	(1.04-2.02)	.	.	5.8	5.8	1.0
	Stroke	2,436,347	3.3	3.1	2.7	3.6	1.24	(0.94-1.62)	1.26	(0.94-1.68)	1.37	(0.95-1.96)	.	.	.	.	.
	Violence	.	.	.	.	.	.	.	.	.	.	.	4.4	17.0	22.0	43.4	.
	Heavy drinking	2,288,632	5.1	7.4	9.2	8.8	1.38	(1.15-1.66)	1.72	(1.44-2.07)	1.80	(1.48-2.19)	5.3	10.4	9.5	25.1	.
	Illicit drug use	.	.	.	.	.	.	.	.	.	.	.	6.2	15.9	30.6	52.7	.
	OVOB	2,203,441	68.0	70.8	70.3	70.4	1.23	(1.10-1.36)	1.22	(1.09-1.36)	1.39	(1.22-1.57)	1.1	1.1	1.5	3.8	.
	Smoking	2,336,294	8.2	12.9	19.8	32.8	1.44	(1.24-1.68)	2.29	(1.99-2.63)	4.03	(3.50-4.63)	4.0	12.0	22.3	38.3	.
Kansas	Anxiety	.	.	.	.	.	.	.	.	.	.	.	6.1	12.6	10.4	29.1	.
	Arthritis	2,204,241	23.0	23.3	26.2	28.7	1.71	(1.51-1.94)	2.06	(1.82-2.33)	2.98	(2.59-3.43)	4.5	8.5	14.3	27.2	20.2
	Asthma	2,207,283	7.1	10.5	15.8	25.5	1.69	(1.41-2.02)	2.11	(1.79-2.48)	3.49	(2.96-4.10)	5.2	11.5	29.1	45.8	37.2
	Cancer	2,209,194	8.5	6.6	6.1	6.4	1.10	(0.92-1.31)	1.06	(0.88-1.27)	1.50	(1.25-1.81)	.	.	7.9	7.9	6.0
	COPD	2,203,393	3.5	4.9	6.4	11.5	1.88	(1.48-2.39)	2.62	(2.07-3.31)	4.57	(3.50-5.97)	5.1	13.1	31.7	49.9	35.1
	Depression	2,203,602	3.8	7.3	17.9	50.5	2.77	(2.22-3.47)	5.50	(4.54-6.67)	21.34	(17.46-26.08)	4.9	15.9	58.4	79.2	77.2
	Diabetes	2,211,710	12.2	9.1	11.0	10.8	0.92	(0.78-1.08)	1.32	(1.14-1.54)	1.51	(1.26-1.80)	.	4.4	6.5	10.9	2.0
	Heart disease	2,197,488	4.5	4.0	4.0	3.8	1.37	(1.09-1.73)	1.69	(1.34-2.12)	2.31	(1.77-3.02)	3.8	8.6	12.6	25.0	11.4
	Kidney disease	2,207,392	2.9	2.5	3.2	3.5	1.11	(0.83-1.50)	1.71	(1.30-2.24)	1.79	(1.30-2.46)	.	10.3	12.1	22.4	12.2
	Stroke	2,208,717	3.0	2.1	3.1	3.0	1.03	(0.76-1.38)	1.51	(1.16-1.98)	1.36	(0.98-1.88)	.	8.1	.	8.1	5.2
	Violence	.	.	.	.	.	.	.	.	.	.	.	4.4	17.0	22.0	43.4	.
	Heavy drinking	2,064,386	2.7	6.6	8.2	8.9	2.28	(1.80-2.89)	2.98	(2.41-3.70)	3.10	(2.46-3.89)	9.4	19.6	23.7	52.7	.
	Illicit drug use	.	.	.	.	.	.	.	.	.	.	.	6.2	15.9	30.6	52.7	.
	OVOB	2,004,584	67.1	71.0	71.4	71.5	1.26	(1.12-1.41)	1.27	(1.14-1.41)	1.45	(1.29-1.63)	1.0	1.4	2.4	4.9	.
	Smoking	2,094,114	5.4	6.9	16.9	37.0	1.51	(1.24-1.84)	3.26	(2.76-3.85)	7.34	(6.20-8.69)	2.4	14.6	41.3	58.4	.
Kentucky	Anxiety	.	.	.	.	.	.	.	.	.	.	.	6.1	12.6	10.4	29.1	.
	Arthritis	3,460,665	32.0	27.8	33.0	39.8	1.64	(1.31-2.04)	2.24	(1.86-2.69)	3.50	(2.83-4.33)	2.4	7.4	15.7	25.5	19.1
	Asthma	3,461,052	9.0	10.1	17.2	24.8	1.28	(0.95-1.74)	2.40	(1.91-3.02)	3.96	(3.16-4.98)	.	12.5	33.4	45.9	38.0
	Cancer	3,469,525	9.4	7.0	6.9	7.5	1.13	(0.75-1.68)	1.09	(0.84-1.42)	1.34	(1.01-1.77)	.	.	6.6	6.6	0.6
	COPD	3,462,405	8.0	8.3	11.0	19.4	1.57	(1.14-2.16)	2.56	(1.95-3.35)	4.85	(3.65-6.43)	2.5	10.4	31.6	44.5	26.3
	Depression	3,459,605	6.7	9.7	26.4	56.9	2.54	(1.87-3.45)	9.43	(7.32-12.14)	41.05	(31.27-53.88)	2.5	18.9	61.5	82.9	81.5
	Diabetes	3,473,874	15.3	10.0	13.7	12.4	0.96	(0.71-1.28)	1.42	(1.14-1.77)	1.47	(1.16-1.87)	.	4.7	6.4	11.0	2.1
	Heart disease	3,433,058	5.3	6.4	5.4	7.2	1.68	(1.19-2.39)	1.62	(1.22-2.17)	2.55	(1.85-3.51)	4.2	6.6	20.1	30.9	13.6
	Kidney disease	3,467,131	3.6	3.4	3.7	4.8	1.31	(0.84-2.06)	1.80	(1.23-2.63)	2.83	(1.94-4.13)	.	9.1	21.7	30.8	12.8
	Stroke	3,466,917	4.6	3.8	4.8	5.8	1.34	(0.86-2.10)	1.65	(1.18-2.32)	2.44	(1.67-3.55)	.	7.0	19.1	26.1	14.9
	Violence	.	.	.	.	.	.	.	.	.	.	.	4.4	17.0	22.0	43.4	.
	Heavy drinking	3,287,184	2.6	8.1	7.2	9.4	3.35	(2.19-5.12)	2.59	(1.84-3.63)	4.61	(3.28-6.50)	11.9	14.1	33.7	59.7	.

© 2023 Peterson C et al. *JAMA Network Open.*

	Illicit drug use	.	.	.	.	.	.	.	.	.	.	.	6.2	15.9	30.6	52.7	.
	OVOB	3,239,034	69.9	71.2	72.7	70.7	1.04	(0.84-1.28)	1.18	(0.99-1.41)	1.13	(0.94-1.35)	.	.	.	.	.
	Smoking	3,343,035	8.6	17.7	23.8	42.1	2.86	(2.20-3.71)	3.98	(3.19-4.96)	8.78	(7.11-10.84)	6.1	14.2	42.9	63.2	.
Louisiana	Anxiety	.	.	.	.	.	.	.	.	.	.	.	6.1	12.6	10.4	29.1	.
	Arthritis	3,542,997	27.8	23.2	28.5	32.4	1.91	(1.55-2.36)	2.67	(2.20-3.25)	4.58	(3.59-5.85)	5.6	9.0	15.7	30.3	26.9
	Asthma	3,553,230	8.4	11.1	14.4	25.6	1.74	(1.34-2.27)	2.15	(1.68-2.75)	4.55	(3.48-5.95)	7.0	10.1	29.6	46.7	43.4
	Cancer	3,551,662	9.3	5.7	7.0	6.8	0.81	(0.60-1.09)	1.14	(0.85-1.52)	1.82	(1.28-2.58)	.	.	9.2	9.2	3.1
	COPD	3,545,290	5.0	5.4	8.9	16.7	2.12	(1.46-3.08)	3.51	(2.49-4.96)	8.63	(5.86-12.73)	6.5	13.4	38.5	58.5	53.8
	Depression	3,535,508	4.6	3.5	36.1	61.1	5.13	(3.26-8.06)	82.95	(50.66-135.82)	505.33	(287.53-888.10)	3.2	28.4	61.7	93.3	93.4
	Diabetes	3,555,098	17.7	10.3	12.8	12.5	0.84	(0.64-1.10)	1.27	(1.01-1.58)	1.51	(1.14-2.01)	.	2.6	5.0	7.6	0.6
	Heart disease	3,527,659	5.4	4.6	4.9	5.2	1.49	(1.06-2.10)	1.75	(1.26-2.44)	2.70	(1.82-3.98)	4.6	6.9	13.1	24.6	13.2
	Kidney disease	3,551,680	3.8	3.2	4.3	4.5	1.14	(0.74-1.74)	1.93	(1.30-2.85)	1.80	(1.14-2.84)	.	10.1	11.3	21.4	10.9
	Stroke	3,550,813	4.3	3.5	3.7	6.3	1.24	(0.83-1.83)	1.22	(0.81-1.83)	1.84	(1.16-2.94)	.	.	13.8	13.8	4.1
	Violence	.	.	.	.	.	.	.	.	.	.	.	4.4	17.0	22.0	43.4	.
	Heavy drinking	3,199,256	2.8	7.6	10.6	10.5	5.07	(3.57-7.21)	6.16	(4.41-8.59)	7.26	(5.07-10.39)	20.7	25.7	27.9	74.3	.
	Illicit drug use	.	.	.	.	.	.	.	.	.	.	.	6.2	15.9	30.6	52.7	.
	OVOB	3,223,493	73.7	68.7	69.3	70.2	0.94	(0.78-1.13)	1.06	(0.88-1.28)	1.08	(0.88-1.32)	.	.	.	.	.
	Smoking	3,327,269	7.3	13.7	23.3	40.6	4.33	(3.30-5.69)	6.49	(4.97-8.47)	13.45	(10.22-17.70)	13.4	18.9	40.5	72.8	.
Maine	Anxiety	.	.	.	.	.	.	.	.	.	.	.	6.1	12.6	10.4	29.1	.
	Arthritis	1,088,530	27.4	27.6	30.4	32.8	2.13	(1.83-2.47)	2.75	(2.39-3.16)	5.75	(4.78-6.92)	6.1	12.4	22.3	40.9	35.4
	Asthma	1,091,156	7.9	7.3	12.0	27.0	1.44	(1.12-1.84)	2.51	(2.04-3.08)	6.37	(5.03-8.07)	2.4	12.3	43.9	58.7	54.3
	Cancer	1,093,351	12.0	9.1	7.4	7.2	1.16	(0.95-1.42)	1.09	(0.91-1.31)	1.59	(1.23-2.05)	.	.	8.3	8.3	3.3
	COPD	1,090,951	5.0	7.6	7.5	13.4	2.89	(2.21-3.79)	3.97	(3.14-5.02)	8.88	(6.57-12.01)	7.9	17.5	38.9	64.3	54.8
	Depression	1,090,742	3.0	3.1	14.5	54.8	6.41	(3.89-10.57)	58.92	(36.62-94.80)	640.92	(366.08-1122.12)	2.0	21.1	73.8	96.9	96.9
	Diabetes	1,094,071	11.2	11.3	11.2	9.1	1.25	(1.02-1.53)	1.64	(1.37-1.96)	1.64	(1.29-2.08)	2.6	9.0	7.5	19.1	3.9
	Heart disease	1,088,842	5.5	6.1	5.5	4.2	1.83	(1.43-2.33)	1.87	(1.50-2.34)	2.29	(1.69-3.10)	8.2	10.5	11.6	30.3	9.9
	Kidney disease	1,091,332	3.3	2.8	3.7	3.1	1.53	(1.11-2.11)	2.36	(1.76-3.17)	3.04	(2.04-4.51)	5.2	17.3	17.9	40.3	15.9
	Stroke	1,092,454	3.7	3.1	3.2	3.6	1.43	(1.05-1.96)	1.64	(1.24-2.16)	2.24	(1.59-3.15)	4.3	8.5	16.3	29.1	18.1
	Violence	.	.	.	.	.	.	.	.	.	.	.	4.4	17.0	22.0	43.4	.
	Heavy drinking	1,022,352	3.0	8.9	8.2	12.0	4.86	(3.74-6.31)	4.04	(3.13-5.20)	6.92	(5.28-9.06)	17.1	19.3	37.0	73.5	.
	Illicit drug use	.	.	.	.	.	.	.	.	.	.	.	6.2	15.9	30.6	52.7	.
	OVOB	983,138	64.0	67.3	67.4	64.5	1.38	(1.19-1.59)	1.43	(1.25-1.63)	1.52	(1.30-1.77)	1.6	2.5	2.9	7.1	.
	Smoking	1,058,607	4.6	6.7	11.8	36.6	3.07	(2.29-4.12)	5.62	(4.36-7.25)	22.23	(17.05-28.99)	5.1	15.5	59.6	80.3	.
Maryland	Anxiety	.	.	.	.	.	.	.	.	.	.	.	6.1	12.6	10.4	29.1	.
	Arthritis	4,711,806	23.2	19.7	24.3	29.4	1.82	(1.59-2.09)	2.47	(2.19-2.79)	4.17	(3.65-4.76)	3.7	9.8	16.6	30.1	25.3
	Asthma	4,726,233	9.3	13.9	12.2	24.9	2.14	(1.77-2.57)	1.54	(1.28-1.86)	3.42	(2.91-4.03)	7.6	6.2	28.3	42.1	35.6
	Cancer	4,732,007	8.8	6.6	5.9	5.5	1.11	(0.89-1.37)	1.03	(0.86-1.23)	1.08	(0.89-1.31)	.	.	.	.	.
	COPD	4,718,866	3.8	4.3	5.6	9.2	2.18	(1.66-2.87)	2.72	(2.10-3.52)	5.08	(3.96-6.51)	5.8	13.0	30.8	49.6	42.3
	Depression	4,710,014	4.0	5.2	10.4	52.2	2.75	(1.88-4.01)	6.59	(4.80-9.03)	67.45	(48.20-94.37)	2.6	10.6	73.6	86.9	86.7
	Diabetes	4,730,530	13.1	8.5	10.5	9.0	1.01	(0.83-1.22)	1.21	(1.04-1.41)	1.26	(1.06-1.49)	.	2.6	2.4	5.0	.
	Heart disease	4,712,140	4.1	3.3	3.4	2.9	1.23	(0.96-1.56)	2.01	(1.63-2.48)	1.87	(1.44-2.41)	.	11.3	8.2	19.5	7.7
	Kidney disease	4,727,622	2.5	2.5	3.2	2.9	1.28	(0.93-1.77)	2.26	(1.72-2.97)	1.82	(1.29-2.58)	.	15.2	9.4	24.6	13.3
	Stroke	4,728,194	3.1	2.7	2.7	3.9	1.33	(0.98-1.81)	1.28	(0.98-1.67)	2.05	(1.48-2.85)	.	.	12.8	12.8	6.1
	Violence	.	.	.	.	.	.	.	.	.	.	.	4.4	17.0	22.0	43.4	.
	Heavy drinking	4,237,351	2.3	5.3	4.9	9.8	2.56	(1.93-3.39)	1.93	(1.48-2.52)	4.82	(3.76-6.18)	9.9	8.7	37.7	56.4	.
	Illicit drug use	.	.	.	.	.	.	.	.	.	.	.	6.2	15.9	30.6	52.7	.
	OVOB	4,202,165	65.4	67.0	68.5	67.2	1.21	(1.07-1.37)	1.25	(1.11-1.40)	1.28	(1.14-1.44)	0.7	1.1	1.4	3.3	.
	Smoking	4,428,447	5.1	7.8	15.2	25.5	1.78	(1.43-2.22)	3.80	(3.17-4.56)	6.98	(5.87-8.29)	3.5	16.4	38.0	58.0	.

© 2023 Peterson C et al. *JAMA Network Open.*

Massachusetts	Anxiety	.	.	.	.	.	.	.	.	.	.	.	6.1	12.6	10.4	29.1	.
	Arthritis	5,521,705	22.9	23.3	23.6	31.2	1.61	(1.34-1.92)	2.11	(1.78-2.49)	3.92	(3.24-4.75)	3.7	8.2	16.5	28.4	21.8
	Asthma	5,548,096	8.5	11.9	15.8	30.8	1.85	(1.45-2.35)	2.38	(1.93-2.93)	5.60	(4.54-6.91)	5.2	11.8	36.5	53.4	47.9
	Cancer	5,556,881	9.4	7.0	6.2	6.7	1.09	(0.85-1.39)	1.15	(0.89-1.48)	1.58	(1.24-2.02)	.	.	7.5	7.5	0.2
	COPD	5,543,651	3.1	4.3	5.3	9.0	2.65	(1.86-3.77)	4.22	(3.06-5.82)	7.31	(5.22-10.24)	7.8	18.1	33.3	59.2	46.1
	Depression	5,525,296	3.5	6.7	18.7	57.3	3.82	(2.61-5.61)	12.56	(8.94-17.65)	63.18	(43.56-91.63)	4.2	20.1	64.5	88.7	88.2
	Diabetes	5,563,499	9.1	7.5	8.3	9.1	0.93	(0.72-1.21)	1.60	(1.27-2.02)	1.39	(1.05-1.82)	.	7.9	5.0	12.9	5.9
	Heart disease	5,535,233	4.3	4.0	3.5	3.8	1.39	(0.99-1.96)	2.23	(1.57-3.15)	2.78	(1.91-4.03)	.	11.1	13.6	24.8	17.6
	Kidney disease	5,551,119	2.4	3.0	2.0	3.0	2.10	(1.33-3.33)	2.09	(1.29-3.40)	2.01	(1.27-3.18)	9.4	12.6	13.2	35.1	18.1
	Stroke	5,555,293	2.6	2.0	2.0	3.1	0.97	(0.63-1.49)	1.53	(1.03-2.27)	1.92	(1.22-3.02)	.	6.8	13.5	20.3	16.6
	Violence	.	.	.	.	.	.	.	.	.	.	.	4.4	17.0	22.0	43.4	.
	Heavy drinking	5,088,642	3.1	9.3	9.0	11.4	2.94	(2.27-3.81)	2.52	(1.96-3.24)	3.92	(3.05-5.04)	13.2	13.9	27.3	54.4	.
	Illicit drug use	.	.	.	.	.	.	.	.	.	.	.	6.2	15.9	30.6	52.7	.
	OVOB	4,746,870	59.3	62.6	59.3	62.1	1.16	(1.00-1.34)	1.20	(1.05-1.38)	1.39	(1.20-1.60)	.	1.2	2.3	3.5	.
	Smoking	5,262,743	3.7	7.3	12.7	31.2	2.18	(1.60-2.96)	3.94	(2.98-5.21)	10.96	(8.30-14.46)	4.9	15.4	46.9	67.2	.
Michigan	Anxiety	.	.	.	.	.	.	.	.	.	.	.	6.1	12.6	10.4	29.1	.
	Arthritis	7,813,566	27.7	27.4	32.5	33.2	1.57	(1.37-1.79)	1.93	(1.69-2.21)	2.80	(2.38-3.28)	4.6	7.3	11.4	23.3	15.9
	Asthma	7,832,654	7.9	12.9	16.9	25.4	1.52	(1.26-1.84)	2.01	(1.68-2.41)	2.84	(2.35-3.44)	5.7	11.4	22.8	39.9	30.0
	Cancer	7,845,507	9.6	7.9	7.0	6.9	1.06	(0.89-1.27)	0.95	(0.79-1.15)	1.28	(1.03-1.60)	.	.	4.1	4.1	1.8
	COPD	7,820,546	4.8	8.2	8.9	12.8	1.82	(1.44-2.30)	2.15	(1.71-2.72)	3.47	(2.71-4.45)	7.6	11.3	23.3	42.2	27.0
	Depression	7,821,387	4.7	7.3	19.9	47.5	1.82	(1.48-2.24)	4.53	(3.74-5.47)	11.98	(9.82-14.62)	4.3	17.4	46.8	68.6	65.2
	Diabetes	7,850,663	13.2	9.5	13.2	10.5	0.97	(0.81-1.15)	1.28	(1.08-1.51)	1.45	(1.19-1.77)	.	4.0	5.4	9.4	3.3
	Heart disease	7,811,884	5.7	4.5	5.2	3.9	1.10	(0.86-1.40)	1.46	(1.15-1.85)	1.59	(1.21-2.09)	.	7.2	7.5	14.8	1.7
	Kidney disease	7,839,289	3.0	2.8	3.5	3.6	1.22	(0.91-1.64)	1.36	(1.02-1.82)	1.62	(1.18-2.22)	.	5.8	8.9	14.7	1.7
	Stroke	7,845,788	3.1	3.0	3.8	3.9	1.27	(0.95-1.70)	1.53	(1.15-2.05)	2.03	(1.44-2.85)	.	7.8	14.0	21.8	7.1
	Violence	.	.	.	.	.	.	.	.	.	.	.	4.4	17.0	22.0	43.4	.
	Heavy drinking	7,480,262	3.3	6.6	8.0	9.1	1.49	(1.15-1.92)	2.03	(1.59-2.58)	2.46	(1.90-3.18)	6.3	13.5	20.0	39.8	.
	Illicit drug use	.	.	.	.	.	.	.	.	.	.	.	6.2	15.9	30.6	52.7	.
	OVOB	7,293,990	69.1	68.7	71.8	70.3	1.13	(1.00-1.29)	1.31	(1.16-1.49)	1.32	(1.15-1.52)	0.7	1.5	1.7	3.9	.
	Smoking	7,576,311	7.0	11.4	18.8	37.9	1.54	(1.26-1.88)	2.78	(2.31-3.34)	5.09	(4.24-6.11)	4.1	13.4	30.9	48.5	.
Minnesota	Anxiety	.	.	.	.	.	.	.	.	.	.	.	6.1	12.6	10.4	29.1	.
	Arthritis	4,353,455	20.1	20.6	21.7	27.1	2.35	(2.13-2.60)	3.12	(2.77-3.52)	6.95	(6.01-8.03)	9.9	10.0	16.4	36.2	31.4
	Asthma	4,359,636	5.0	10.8	15.6	25.4	3.35	(2.90-3.86)	5.17	(4.45-6.00)	9.75	(8.33-11.43)	15.7	18.4	33.6	67.7	67.0
	Cancer	4,366,963	8.8	5.8	5.2	5.6	1.12	(0.98-1.29)	1.14	(0.96-1.36)	1.52	(1.21-1.90)	.	.	4.5	4.5	1.4
	COPD	4,357,563	2.0	4.3	4.8	9.3	4.77	(3.84-5.91)	7.08	(5.56-9.01)	19.57	(14.97-25.57)	17.3	17.8	36.2	71.4	65.9
	Depression	4,351,595	1.8	8.6	27.4	62.7	32.90	(23.30-46.44)	278.78	(191.40-406.05)	1874.78	(1240.51-2833.35)	10.1	28.6	58.0	96.7	97.0
	Diabetes	4,367,056	10.0	7.5	8.7	8.0	1.14	(0.99-1.31)	1.55	(1.32-1.83)	1.73	(1.41-2.11)	.	5.4	5.6	11.0	4.4
	Heart disease	4,349,433	4.3	3.2	3.4	3.0	1.56	(1.29-1.89)	2.49	(1.98-3.15)	3.93	(2.96-5.20)	7.4	10.0	11.1	28.5	18.9
	Kidney disease	4,361,970	2.4	2.2	2.7	2.8	1.50	(1.20-1.89)	2.66	(2.05-3.46)	3.27	(2.38-4.51)	6.7	12.8	14.6	34.1	15.9
	Stroke	4,363,865	2.8	2.4	2.6	3.4	1.76	(1.39-2.23)	1.92	(1.43-2.58)	3.27	(2.32-4.61)	9.1	7.6	15.1	31.9	23.8
	Violence	.	.	.	.	.	.	.	.	.	.	.	4.4	17.0	22.0	43.4	.
	Heavy drinking	4,064,704	2.5	7.5	11.9	9.8	3.52	(3.00-4.14)	5.93	(5.03-7.00)	4.59	(3.81-5.52)	19.5	26.3	18.9	64.7	.
	Illicit drug use	.	.	.	.	.	.	.	.	.	.	.	6.2	15.9	30.6	52.7	.
	OVOB	3,921,361	65.3	68.4	65.4	67.0	1.35	(1.24-1.47)	1.38	(1.25-1.51)	1.79	(1.60-2.00)	2.1	1.7	3.0	6.8	.
	Smoking	4,155,946	3.4	8.4	19.3	36.8	4.03	(3.34-4.87)	14.07	(11.71-16.91)	30.53	(25.11-37.13)	10.6	24.9	43.8	79.3	.
Mississippi	Anxiety	.	.	.	.	.	.	.	.	.	.	.	6.1	12.6	10.4	29.1	.
	Arthritis	2,274,845	29.8	27.9	30.0	32.3	1.20	(1.04-1.39)	1.43	(1.20-1.69)	2.06	(1.70-2.51)	2.0	3.5	5.8	11.3	6.3

© 2023 Peterson C et al. *JAMA Network Open.*

	Asthma	2,274,321	9.9	12.6	16.4	23.5	1.30	(1.05-1.61)	1.66	(1.33-2.06)	2.43	(1.96-3.03)	4.2	8.1	14.4	26.7	18.2
	Cancer	2,277,139	7.8	5.8	6.2	6.5	0.86	(0.68-1.10)	1.05	(0.82-1.36)	1.51	(1.10-2.07)	.	.	5.3	5.3	2.0
	COPD	2,273,287	6.7	7.7	10.7	14.6	1.29	(1.02-1.65)	1.80	(1.42-2.30)	2.54	(1.94-3.32)	3.8	9.0	13.8	26.5	14.9
	Depression	2,270,108	11.5	15.1	25.7	44.4	1.33	(1.09-1.62)	2.46	(2.01-3.00)	4.73	(3.85-5.82)	3.4	12.1	22.0	37.4	33.0
	Diabetes	2,279,542	16.0	14.0	13.6	14.2	1.00	(0.84-1.20)	1.05	(0.87-1.26)	1.56	(1.23-1.98)	.	.	4.5	4.5	2.4
	Heart disease	2,263,303	5.8	5.0	5.1	5.3	1.07	(0.83-1.38)	1.29	(0.97-1.72)	1.60	(1.13-2.27)	.	.	5.3	5.3	1.3
	Kidney disease	2,276,880	2.9	3.1	2.4	2.9	1.44	(1.00-2.06)	1.08	(0.75-1.55)	1.62	(1.05-2.50)	7.5	.	6.4	13.9	7.2
	Stroke	2,278,008	4.6	5.2	5.5	5.8	1.24	(0.94-1.64)	1.35	(0.99-1.84)	1.77	(1.23-2.55)	.	.	7.8	7.8	5.3
	Violence	.	.	.	.	.	.	.	.	.	.	.	4.4	17.0	22.0	43.4	.
	Heavy drinking	2,124,960	3.4	6.4	6.2	9.5	1.95	(1.40-2.70)	1.68	(1.18-2.39)	2.71	(1.94-3.79)	12.0	8.0	17.7	37.7	.
	Illicit drug use	.	.	.	.	.	.	.	.	.	.	.	6.2	15.9	30.6	52.7	.
	OVOB	2,121,456	72.3	74.0	71.7	73.4	1.10	(0.94-1.28)	1.02	(0.87-1.20)	1.27	(1.05-1.53)	.	.	1.0	1.0	.
	Smoking	2,210,288	12.0	19.2	24.4	35.9	1.61	(1.34-1.94)	2.07	(1.72-2.50)	3.46	(2.83-4.22)	6.5	10.0	17.5	34.1	.
Missouri	Anxiety	.	.	.	.	.	.	.	.	.	.	.	6.1	12.6	10.4	29.1	.
	Arthritis	4,753,953	27.0	25.8	27.1	30.5	1.25	(1.09-1.44)	1.50	(1.29-1.73)	2.35	(2.00-2.77)	2.5	4.5	9.4	16.4	11.1
	Asthma	4,767,045	8.8	11.2	15.9	23.0	1.21	(0.99-1.49)	1.72	(1.42-2.09)	2.20	(1.81-2.68)	.	9.5	16.9	26.4	19.6
	Cancer	4,772,519	8.6	6.8	7.0	8.0	0.95	(0.78-1.16)	1.06	(0.86-1.31)	1.64	(1.31-2.04)	.	.	8.4	8.4	5.9
	COPD	4,764,698	6.0	7.7	8.9	13.8	1.50	(1.18-1.90)	1.63	(1.30-2.04)	2.92	(2.27-3.76)	5.4	7.1	18.7	31.3	20.6
	Depression	4,755,358	9.1	16.0	24.4	48.5	1.90	(1.59-2.28)	2.76	(2.32-3.27)	7.18	(6.04-8.54)	6.3	12.7	33.8	52.9	50.2
	Diabetes	4,772,161	12.0	9.4	10.4	9.9	0.86	(0.72-1.03)	1.09	(0.90-1.30)	1.23	(0.99-1.53)	.	.	.	.	.
	Heart disease	4,755,720	5.0	3.9	5.4	4.0	0.95	(0.73-1.23)	1.61	(1.25-2.07)	1.56	(1.15-2.11)	.	8.8	6.3	15.1	5.4
	Kidney disease	4,765,052	3.2	2.6	3.3	3.3	0.97	(0.70-1.33)	1.28	(0.93-1.76)	1.68	(1.19-2.38)	.	.	9.6	9.6	5.0
	Stroke	4,768,098	3.3	4.0	4.4	4.5	1.47	(1.13-1.93)	1.74	(1.32-2.30)	1.97	(1.44-2.70)	6.5	9.6	11.7	27.7	17.7
	Violence	.	.	.	.	.	.	.	.	.	.	.	4.4	17.0	22.0	43.4	.
	Heavy drinking	4,491,333	4.3	6.5	8.4	9.0	1.30	(1.00-1.69)	1.64	(1.28-2.10)	1.83	(1.43-2.34)	4.4	9.6	12.6	26.6	.
	Illicit drug use	.	.	.	.	.	.	.	.	.	.	.	6.2	15.9	30.6	52.7	.
	OVOB	4,400,517	65.3	71.4	68.7	71.5	1.35	(1.18-1.55)	1.27	(1.11-1.45)	1.55	(1.34-1.80)	1.8	1.5	2.7	6.1	.
	Smoking	4,617,059	9.5	14.3	21.4	34.4	1.43	(1.19-1.72)	2.26	(1.91-2.67)	3.69	(3.13-4.35)	3.7	11.6	23.1	38.5	.
Montana	Anxiety	.	.	.	.	.	.	.	.	.	.	.	6.1	12.6	10.4	29.1	.
	Arthritis	842,936	25.2	25.8	27.2	31.6	1.68	(1.43-1.97)	2.01	(1.75-2.32)	3.29	(2.78-3.90)	4.3	8.8	15.9	29.0	21.3
	Asthma	844,844	9.0	12.6	13.6	24.0	1.46	(1.18-1.82)	1.52	(1.26-1.85)	2.49	(2.05-3.04)	3.9	7.3	23.1	34.3	21.9
	Cancer	844,867	9.6	7.6	6.7	7.3	1.10	(0.87-1.40)	1.12	(0.90-1.38)	1.56	(1.22-1.99)	.	.	8.3	8.3	3.8
	COPD	842,491	4.3	5.3	6.2	10.6	1.80	(1.32-2.46)	2.42	(1.85-3.15)	4.40	(3.31-5.86)	5.1	13.3	31.4	49.8	39.7
	Depression	841,925	6.3	10.4	21.6	51.9	2.88	(2.23-3.71)	6.00	(4.84-7.44)	21.39	(16.97-26.97)	4.8	18.7	54.6	78.1	75.2
	Diabetes	845,731	9.6	7.3	8.7	7.5	0.96	(0.76-1.21)	1.20	(0.97-1.48)	1.28	(1.00-1.63)	.	.	.	.	.
	Heart disease	840,152	3.9	3.7	3.7	3.4	1.22	(0.89-1.69)	1.57	(1.17-2.10)	2.07	(1.45-2.96)	.	8.7	12.2	20.9	8.5
	Kidney disease	844,237	2.5	2.3	2.8	2.8	1.15	(0.77-1.72)	1.70	(1.20-2.41)	1.80	(1.22-2.65)	.	11.4	12.8	24.2	12.8
	Stroke	844,701	3.3	2.4	2.5	3.4	1.22	(0.82-1.82)	1.14	(0.80-1.61)	1.72	(1.15-2.60)	.	.	12.4	12.4	.
	Violence	.	.	.	.	.	.	.	.	.	.	.	4.4	17.0	22.0	43.4	.
	Heavy drinking	804,830	4.3	9.2	10.2	14.0	2.10	(1.62-2.72)	2.14	(1.70-2.70)	3.04	(2.38-3.88)	8.5	13.6	26.3	48.4	.
	Illicit drug use	.	.	.	.	.	.	.	.	.	.	.	6.2	15.9	30.6	52.7	.
	OVOB	785,917	64.1	65.8	65.8	63.2	1.12	(0.97-1.29)	1.21	(1.06-1.37)	1.24	(1.07-1.42)	.	1.3	1.5	2.8	.
	Smoking	818,017	7.0	9.1	15.8	31.9	1.51	(1.17-1.94)	2.38	(1.95-2.92)	5.43	(4.43-6.65)	2.8	11.6	36.9	51.3	.
Nebraska	Anxiety	.	.	.	.	.	.	.	.	.	.	.	6.1	12.6	10.4	29.1	.
	Arthritis	1,463,325	22.5	21.3	26.1	27.1	2.53	(2.24-2.85)	3.73	(3.27-4.25)	7.29	(6.13-8.66)	9.0	15.0	15.4	39.4	36.0
	Asthma	1,465,682	5.6	9.2	17.8	26.4	2.80	(2.30-3.39)	6.20	(5.14-7.47)	11.93	(9.67-14.71)	9.3	27.6	35.0	71.9	69.8
	Cancer	1,467,062	8.6	5.2	5.8	6.5	1.12	(0.96-1.32)	1.20	(1.00-1.44)	1.80	(1.41-2.28)	.	3.0	6.7	9.8	3.8
	COPD	1,465,000	2.6	4.3	8.6	12.9	3.67	(2.95-4.56)	9.42	(7.53-11.78)	24.92	(19.15-32.44)	9.8	26.4	38.1	74.3	66.2

© 2023 Peterson C et al. *JAMA Network Open.*

	Depression	1,463,490	1.0	8.3	29.7	61.1	14.22	(9.16-22.07)	160.74	(100.74-256.47)	1898.51	(1142.93-3153.59)	4.1	27.9	65.9	97.9	98.1
	Diabetes	1,467,003	10.4	8.8	11.3	9.7	1.27	(1.08-1.49)	1.89	(1.60-2.23)	2.10	(1.69-2.63)	3.1	10.1	7.5	20.7	4.9
	Heart disease	1,461,018	4.1	3.1	4.4	3.6	1.42	(1.15-1.75)	2.65	(2.11-3.32)	3.80	(2.75-5.24)	5.1	14.3	12.4	31.8	19.7
	Kidney disease	1,465,627	2.2	2.3	3.6	2.6	1.61	(1.19-2.17)	2.71	(2.00-3.66)	2.57	(1.69-3.90)	6.8	19.4	11.7	38.0	25.3
	Stroke	1,467,174	2.6	1.8	3.3	3.3	1.29	(0.97-1.72)	2.21	(1.64-2.97)	4.10	(2.93-5.75)	.	12.0	18.1	30.1	23.6
	Violence	.	.	.	.	.	.	.	.	.	.	.	4.4	17.0	22.0	43.4	.
	Heavy drinking	1,383,389	2.8	8.5	10.8	9.9	3.56	(2.87-4.43)	4.78	(3.88-5.89)	5.33	(4.18-6.80)	17.2	27.4	21.6	66.2	.
	Illicit drug use	.	.	.	.	.	.	.	.	.	.	.	6.2	15.9	30.6	52.7	.
	OVOB	1,349,127	68.7	70.7	68.2	71.0	1.32	(1.18-1.47)	1.24	(1.10-1.38)	1.67	(1.45-1.92)	1.4	1.3	2.4	5.1	.
	Smoking	1,413,426	3.0	9.9	27.7	40.5	2.51	(2.00-3.15)	12.92	(10.51-15.87)	36.73	(29.23-46.15)	4.4	30.4	47.0	81.8	.
Nevada	Anxiety	.	.	.	.	.	.	.	.	.	.	.	6.1	12.6	10.4	29.1	.
	Arthritis	2,405,337	22.5	23.3	26.2	25.3	1.52	(1.06-2.18)	1.95	(1.42-2.68)	2.52	(1.82-3.49)	3.2	8.2	15.1	26.4	18.1
	Asthma	2,422,486	7.2	13.4	11.8	22.3	2.60	(1.68-4.01)	1.96	(1.34-2.87)	3.97	(2.78-5.67)	8.6	8.8	35.2	52.6	47.6
	Cancer	2,422,927	6.7	6.3	5.9	5.8	1.22	(0.77-1.93)	0.92	(0.57-1.48)	1.48	(0.96-2.27)	.	.	.	.	.
	COPD	2,421,228	5.4	5.7	8.1	10.1	1.19	(0.66-2.15)	2.03	(1.21-3.38)	4.33	(2.60-7.21)	.	9.8	32.7	42.5	28.3
	Depression	2,417,163	6.0	6.2	13.1	37.4	1.55	(0.78-3.06)	3.73	(2.14-6.51)	16.81	(9.61-29.41)	.	11.6	64.3	75.9	73.8
	Diabetes	2,422,967	14.3	9.8	10.6	9.3	0.64	(0.41-0.98)	0.71	(0.50-1.00)	0.87	(0.59-1.29)	.	.	.	.	.
	Heart disease	2,411,854	4.3	6.6	5.2	3.9	1.45	(0.70-3.03)	1.37	(0.79-2.36)	1.63	(0.87-3.03)	.	.	.	.	.
	Kidney disease	2,420,052	2.4	4.0	4.1	3.6	1.89	(0.92-3.88)	1.96	(1.11-3.47)	3.00	(1.67-5.37)	.	10.9	22.8	33.7	13.9
	Stroke	2,418,793	2.8	5.1	3.7	3.1	2.05	(0.93-4.52)	1.94	(1.03-3.65)	1.47	(0.77-2.78)	.	12.4	.	12.4	12.8
	Violence	.	.	.	.	.	.	.	.	.	.	.	4.4	17.0	22.0	43.4	.
	Heavy drinking	2,266,757	2.5	4.4	9.8	9.9	1.87	(1.02-3.45)	4.50	(2.79-7.27)	4.47	(2.78-7.21)	3.9	25.4	36.1	65.4	.
	Illicit drug use	.	.	.	.	.	.	.	.	.	.	.	6.2	15.9	30.6	52.7	.
	OVOB	2,203,003	63.9	67.6	69.3	65.0	1.25	(0.91-1.73)	1.30	(1.03-1.65)	1.38	(1.09-1.75)	.	1.7	3.1	4.8	.
	Smoking	2,337,587	7.7	12.8	13.1	24.3	2.08	(1.38-3.12)	2.21	(1.55-3.15)	5.40	(3.88-7.52)	4.8	8.9	41.3	55.1	.
New Hampshire	Anxiety	.	.	.	.	.	.	.	.	.	.	.	6.1	12.6	10.4	29.1	.
	Arthritis	1,106,694	24.5	24.2	28.5	28.2	2.22	(1.85-2.68)	2.65	(2.19-3.20)	4.40	(3.40-5.68)	6.8	11.6	15.6	34.0	28.4
	Asthma	1,108,217	8.6	14.9	14.2	27.9	1.60	(1.24-2.08)	2.54	(2.02-3.21)	5.51	(4.23-7.18)	4.1	14.2	34.5	52.7	51.0
	Cancer	1,109,029	9.7	7.3	5.6	7.2	1.43	(1.13-1.81)	1.19	(0.94-1.51)	2.16	(1.54-3.02)	4.4	.	10.9	15.3	13.4
	COPD	1,107,845	3.8	6.1	5.8	11.1	4.22	(3.06-5.81)	4.39	(3.21-6.00)	11.60	(7.89-17.07)	11.4	16.2	37.6	65.2	57.7
	Depression	1,108,395	2.7	9.0	32.8	56.8	11.35	(6.10-21.13)	72.75	(39.15-135.22)	479.66	(241.70-951.90)	4.8	27.7	63.7	96.1	96.2
	Diabetes	1,110,232	9.0	8.5	10.7	9.4	1.22	(0.96-1.55)	1.57	(1.25-1.99)	2.41	(1.76-3.29)	.	6.9	12.6	19.5	13.2
	Heart disease	1,106,336	4.5	4.0	4.7	3.2	1.33	(0.95-1.88)	1.68	(1.19-2.38)	2.50	(1.64-3.82)	.	8.7	12.1	20.8	15.1
	Kidney disease	1,109,833	1.9	2.6	3.2	3.0	2.52	(1.61-3.93)	2.54	(1.72-3.74)	2.92	(1.86-4.57)	10.3	15.3	17.0	42.7	25.5
	Stroke	1,109,094	3.0	2.4	3.3	2.4	1.20	(0.75-1.90)	1.59	(1.09-2.31)	2.05	(1.17-3.58)	.	8.1	11.1	19.2	16.5
	Violence	.	.	.	.	.	.	.	.	.	.	.	4.4	17.0	22.0	43.4	.
	Heavy drinking	998,704	3.6	11.1	7.9	11.1	5.32	(3.96-7.14)	3.87	(2.89-5.19)	6.16	(4.43-8.56)	19.7	20.1	28.6	68.5	.
	Illicit drug use	.	.	.	.	.	.	.	.	.	.	.	6.2	15.9	30.6	52.7	.
	OVOB	968,902	65.2	67.6	70.0	67.2	1.35	(1.13-1.61)	1.45	(1.23-1.72)	1.51	(1.23-1.85)	1.3	2.2	2.0	5.4	.
	Smoking	1,025,514	4.0	11.8	13.8	36.9	3.47	(2.46-4.88)	6.06	(4.40-8.35)	27.96	(20.14-38.80)	6.3	16.0	55.9	78.3	.
New Jersey	Anxiety	.	.	.	.	.	.	.	.	.	.	.	6.1	12.6	10.4	29.1	.
	Arthritis	6,917,844	20.1	19.0	23.8	25.8	1.60	(1.29-1.99)	2.57	(2.11-3.14)	2.85	(2.21-3.68)	4.5	12.6	8.8	25.8	19.5
	Asthma	6,943,622	7.5	15.3	12.2	26.3	2.55	(1.96-3.33)	1.95	(1.50-2.54)	4.32	(3.27-5.71)	13.1	10.7	22.9	46.7	40.5
	Cancer	6,951,060	6.8	5.3	5.3	4.2	1.13	(0.83-1.54)	1.18	(0.89-1.56)	1.06	(0.71-1.58)	.	.	.	.	.
	COPD	6,930,633	3.1	4.2	5.8	11.3	2.13	(1.42-3.20)	2.39	(1.65-3.47)	5.01	(3.29-7.63)	9.0	13.1	24.9	47.0	36.9
	Depression	6,924,244	4.4	6.2	17.1	55.4	2.29	(1.61-3.26)	8.26	(6.13-11.14)	53.00	(37.75-74.39)	4.2	23.6	53.2	81.1	80.0
	Diabetes	6,947,175	12.2	8.2	9.6	8.3	0.90	(0.68-1.20)	1.44	(1.13-1.83)	1.28	(0.92-1.80)	.	5.9	.	5.9	3.0

© 2023 Peterson C et al. *JAMA Network Open.*

	Heart disease	6,923,980	4.5	3.4	4.2	3.1	1.04	(0.70-1.55)	1.52	(1.05-2.19)	1.48	(0.89-2.46)	.	6.8	.	6.8	.
	Kidney disease	6,945,301	1.5	3.1	3.1	3.6	2.49	(1.41-4.40)	3.64	(2.22-5.95)	4.66	(2.74-7.95)	12.9	20.9	17.9	51.6	42.0
	Stroke	6,952,670	2.9	1.8	3.3	2.8	0.88	(0.55-1.41)	1.48	(0.94-2.34)	1.87	(1.15-3.05)	.	.	8.3	8.3	7.3
	Violence	.	.	.	.	.	.	.	.	.	.	.	4.4	17.0	22.0	43.4	.
	Heavy drinking	6,021,970	3.0	6.3	8.3	13.9	2.32	(1.70-3.18)	2.62	(1.96-3.50)	4.60	(3.38-6.26)	11.1	18.2	24.6	53.9	.
	Illicit drug use	.	.	.	.	.	.	.	.	.	.	.	6.2	15.9	30.6	52.7	.
	OVOB	5,702,313	62.1	68.1	66.5	68.8	1.21	(1.02-1.42)	1.08	(0.93-1.26)	1.53	(1.26-1.85)	1.0	.	1.8	2.8	.
	Smoking	6,341,972	4.4	7.9	14.6	25.5	2.09	(1.56-2.79)	4.20	(3.25-5.42)	8.67	(6.62-11.37)	6.6	21.7	30.4	58.7	.
New Mexico	Anxiety	.	.	.	.	.	.	.	.	.	.	.	6.1	12.6	10.4	29.1	.
	Arthritis	1,612,151	24.2	23.3	24.6	30.7	1.40	(1.16-1.70)	1.74	(1.46-2.06)	2.98	(2.46-3.61)	2.6	7.4	14.9	24.9	17.8
	Asthma	1,615,026	7.5	11.6	12.1	23.8	1.82	(1.35-2.45)	2.12	(1.65-2.73)	4.18	(3.26-5.37)	5.4	12.2	32.5	50.1	42.0
	Cancer	1,615,025	7.4	6.3	5.8	5.0	0.96	(0.72-1.28)	1.09	(0.84-1.42)	1.27	(0.96-1.67)	.	.	.	.	.
	COPD	1,611,316	3.4	6.5	5.8	6.9	2.85	(2.03-4.00)	2.93	(2.14-4.02)	4.35	(3.12-6.05)	10.6	17.3	25.6	53.6	36.3
	Depression	1,613,692	4.8	7.9	16.0	40.8	2.55	(1.83-3.56)	5.98	(4.51-7.93)	27.27	(20.36-36.54)	3.6	17.7	59.4	80.7	78.8
	Diabetes	1,615,600	14.4	10.5	12.5	10.4	0.88	(0.69-1.12)	1.37	(1.09-1.72)	1.13	(0.87-1.45)	.	5.9	.	5.9	2.7
	Heart disease	1,608,724	3.8	4.0	3.5	2.7	1.32	(0.89-1.94)	1.54	(1.06-2.25)	1.61	(1.08-2.40)	.	8.4	7.1	15.5	7.4
	Kidney disease	1,614,380	3.3	3.1	3.7	3.1	1.41	(0.93-2.13)	1.87	(1.30-2.69)	1.54	(1.02-2.33)	.	13.3	8.2	21.5	15.5
	Stroke	1,613,797	2.6	2.4	2.6	3.1	1.09	(0.72-1.65)	1.66	(1.10-2.51)	2.71	(1.75-4.20)	.	9.4	20.0	29.5	22.4
	Violence	.	.	.	.	.	.	.	.	.	.	.	4.4	17.0	22.0	43.4	.
	Heavy drinking	1,526,187	3.2	4.8	10.2	7.6	1.41	(0.93-2.14)	4.15	(2.96-5.81)	2.94	(2.02-4.29)	.	31.7	20.4	52.2	.
	Illicit drug use	.	.	.	.	.	.	.	.	.	.	.	6.2	15.9	30.6	52.7	.
	OVOB	1,500,465	67.6	67.7	67.1	65.5	1.06	(0.88-1.28)	1.09	(0.93-1.28)	0.94	(0.79-1.12)	.	.	.	.	.
	Smoking	1,555,673	8.1	11.5	15.5	29.5	1.80	(1.35-2.42)	2.42	(1.88-3.13)	4.95	(3.86-6.35)	4.4	13.0	34.4	51.8	.
New York	Anxiety	.	.	.	.	.	.	.	.	.	.	.	6.1	12.6	10.4	29.1	.
	Arthritis	15,306,053	21.4	19.6	23.4	26.7	1.96	(1.70-2.27)	2.42	(2.13-2.76)	4.10	(3.51-4.79)	5.8	9.3	13.8	28.9	22.9
	Asthma	15,367,131	8.2	13.6	14.0	29.5	2.01	(1.68-2.39)	2.05	(1.73-2.44)	4.37	(3.68-5.20)	8.1	9.7	28.0	45.8	38.3
	Cancer	15,365,838	8.5	5.3	6.3	5.5	0.99	(0.82-1.21)	1.14	(0.95-1.37)	1.22	(0.98-1.52)	.	.	.	.	.
	COPD	15,330,537	3.9	5.1	6.4	9.6	2.11	(1.65-2.70)	2.84	(2.25-3.58)	4.86	(3.78-6.25)	7.5	13.7	24.6	45.8	31.2
	Depression	15,309,816	4.0	4.3	16.3	56.6	2.04	(1.43-2.92)	9.77	(7.49-12.75)	79.32	(59.62-105.54)	2.4	17.6	65.2	85.3	85.5
	Diabetes	15,365,602	12.5	9.0	9.8	10.1	1.01	(0.84-1.21)	1.34	(1.13-1.59)	1.30	(1.06-1.60)	.	3.8	2.9	6.7	0.1
	Heart disease	15,280,994	4.3	4.0	3.0	3.6	1.39	(1.07-1.80)	1.42	(1.11-1.81)	2.13	(1.55-2.92)	4.4	4.4	8.8	17.5	6.4
	Kidney disease	15,351,987	2.4	2.6	3.2	3.4	2.01	(1.46-2.77)	3.11	(2.36-4.11)	3.36	(2.43-4.64)	8.4	16.6	16.4	41.4	32.4
	Stroke	15,365,288	2.8	2.4	2.7	3.7	1.09	(0.79-1.50)	1.74	(1.27-2.37)	2.53	(1.83-3.48)	.	7.9	13.9	21.8	7.5
	Violence	.	.	.	.	.	.	.	.	.	.	.	4.4	17.0	22.0	43.4	.
	Heavy drinking	13,616,313	2.8	6.8	7.9	9.8	2.91	(2.33-3.63)	3.17	(2.57-3.91)	4.70	(3.80-5.83)	13.9	18.3	26.6	58.8	.
	Illicit drug use	.	.	.	.	.	.	.	.	.	.	.	6.2	15.9	30.6	52.7	.
	OVOB	13,215,679	62.0	65.3	63.8	66.1	1.09	(0.98-1.22)	1.12	(1.00-1.25)	1.30	(1.15-1.47)	.	0.6	1.4	2.0	.
	Smoking	14,182,728	4.5	9.8	18.0	27.3	2.80	(2.27-3.44)	5.67	(4.71-6.83)	9.79	(8.14-11.77)	9.1	22.2	36.0	67.2	.
North Carolina	Anxiety	.	.	.	.	.	.	.	.	.	.	.	6.1	12.6	10.4	29.1	.
	Arthritis	8,242,449	24.6	22.3	29.0	36.3	2.25	(1.82-2.79)	3.29	(2.69-4.02)	6.76	(5.30-8.63)	5.9	13.1	18.9	37.8	32.7
	Asthma	8,268,487	5.8	10.5	15.9	27.3	1.77	(1.30-2.41)	3.54	(2.77-4.53)	7.59	(5.79-9.96)	5.1	19.5	38.0	62.6	58.6
	Cancer	8,270,368	9.8	5.4	4.7	7.6	0.84	(0.62-1.13)	0.84	(0.64-1.09)	1.15	(0.81-1.63)	.	.	.	.	.
	COPD	8,265,284	3.9	5.1	8.3	16.5	2.99	(2.06-4.35)	4.29	(3.10-5.93)	12.51	(8.39-18.66)	8.1	17.6	40.2	65.9	58.4
	Depression	8,257,112	2.7	6.4	39.9	63.4	6.78	(3.90-11.78)	104.54	(62.33-175.35)	764.28	(426.14-1370.72)	2.8	31.8	60.9	95.5	95.4
	Diabetes	8,275,560	13.5	10.7	14.0	11.3	0.97	(0.74-1.25)	1.74	(1.39-2.17)	1.52	(1.14-2.02)	.	9.4	4.9	14.3	4.3
	Heart disease	8,237,739	4.7	3.6	6.3	5.0	1.38	(0.95-2.01)	2.28	(1.63-3.20)	1.95	(1.25-3.05)	.	15.3	8.5	23.8	6.2
	Kidney disease	8,267,608	3.2	3.1	5.0	4.1	1.50	(0.98-2.29)	2.39	(1.63-3.51)	1.68	(1.03-2.74)	.	17.1	8.1	25.3	14.4
	Stroke	8,263,762	5.2	2.4	4.5	4.8	0.94	(0.63-1.41)	1.64	(1.10-2.44)	1.95	(1.21-3.15)	.	8.2	10.6	18.8	.

© 2023 Peterson C et al. *JAMA Network Open.*

	Violence	.	.	.	.	.	.	.	.	.	.	.	4.4	17.0	22.0	43.4	.
	Heavy drinking	7,604,061	2.4	5.5	7.7	9.8	1.71	(1.15-2.56)	3.56	(2.64-4.80)	5.59	(4.01-7.80)	5.8	25.0	31.0	61.8	.
	Illicit drug use	.	.	.	.	.	.	.	.	.	.	.	6.2	15.9	30.6	52.7	.
	OVOB	7,349,961	67.2	71.9	69.1	70.1	1.23	(1.03-1.47)	1.37	(1.17-1.60)	1.51	(1.25-1.81)	0.8	2.0	2.1	4.8	.
	Smoking	7,923,831	5.1	14.6	20.8	42.9	3.93	(2.94-5.27)	5.83	(4.54-7.49)	21.60	(16.36-28.52)	9.2	19.4	46.4	75.0	.
North Dakota	Anxiety	.	.	.	.	.	.	.	.	.	.	.	6.1	12.6	10.4	29.1	.
	Arthritis	583,489	23.6	23.2	22.7	28.1	1.13	(0.95-1.34)	1.42	(1.19-1.71)	2.46	(1.94-3.12)	.	3.9	8.8	12.7	8.6
	Asthma	583,542	7.5	11.0	12.9	23.0	1.40	(1.07-1.84)	1.63	(1.25-2.12)	2.67	(2.03-3.51)	5.1	7.5	18.7	31.4	24.3
	Cancer	585,025	7.9	7.4	6.0	5.5	1.12	(0.90-1.40)	1.06	(0.82-1.39)	1.49	(1.08-2.05)	.	.	4.3	4.3	3.5
	COPD	583,579	3.9	4.3	5.8	10.1	1.14	(0.83-1.56)	1.67	(1.22-2.28)	3.37	(2.44-4.67)	.	7.3	20.6	27.9	18.1
	Depression	582,715	7.3	15.6	23.6	42.8	2.10	(1.64-2.69)	3.31	(2.61-4.21)	7.67	(5.98-9.86)	8.2	15.4	31.7	55.3	52.1
	Diabetes	584,854	9.4	10.7	9.2	8.0	1.22	(0.98-1.52)	1.13	(0.89-1.45)	1.11	(0.82-1.50)	.	.	.	.	.
	Heart disease	581,708	4.2	4.0	3.5	2.9	1.24	(0.94-1.65)	1.27	(0.93-1.73)	1.79	(1.20-2.68)	.	.	5.5	5.5	2.5
	Kidney disease	584,104	2.8	3.0	2.5	3.1	1.29	(0.88-1.87)	1.10	(0.73-1.66)	1.43	(0.91-2.26)	.	.	.	.	.
	Stroke	584,388	2.7	2.8	2.4	2.9	1.16	(0.81-1.66)	1.26	(0.85-1.86)	1.45	(0.86-2.43)	.	.	.	.	.
	Violence	.	.	.	.	.	.	.	.	.	.	.	4.4	17.0	22.0	43.4	.
	Heavy drinking	559,389	5.5	6.6	10.6	11.0	1.10	(0.80-1.51)	1.91	(1.42-2.59)	1.86	(1.31-2.64)	.	12.6	10.9	23.5	.
	Illicit drug use	.	.	.	.	.	.	.	.	.	.	.	6.2	15.9	30.6	52.7	.
	OVOB	540,454	68.6	71.8	71.2	72.4	1.21	(1.01-1.44)	1.25	(1.04-1.51)	1.48	(1.18-1.86)	1.1	1.2	1.8	4.1	.
	Smoking	569,224	10.1	13.9	18.5	36.5	1.33	(1.05-1.69)	1.78	(1.41-2.24)	3.44	(2.71-4.37)	3.4	7.8	19.9	31.1	.
Ohio	Anxiety	.	.	.	.	.	.	.	.	.	.	.	6.1	12.6	10.4	29.1	.
	Arthritis	9,103,166	26.0	24.6	32.4	35.7	1.70	(1.49-1.93)	2.31	(2.04-2.61)	3.64	(3.13-4.23)	4.4	10.0	14.1	28.4	19.5
	Asthma	9,113,191	6.6	12.1	17.5	27.7	2.15	(1.77-2.61)	2.91	(2.46-3.45)	5.53	(4.60-6.64)	7.5	16.6	32.6	56.7	50.0
	Cancer	9,121,825	8.9	6.7	7.6	6.9	1.21	(1.01-1.45)	1.28	(1.07-1.52)	1.31	(1.06-1.61)	2.6	4.5	4.1	11.2	2.8
	COPD	9,111,115	4.5	6.2	9.4	16.2	2.43	(1.97-3.00)	3.21	(2.66-3.86)	6.40	(5.14-7.96)	7.4	16.1	31.8	55.4	42.3
	Depression	9,089,763	3.7	5.9	26.7	54.3	3.10	(2.42-3.97)	13.81	(11.18-17.07)	56.03	(44.23-70.98)	3.9	26.0	57.6	87.5	86.5
	Diabetes	9,119,637	12.9	10.3	12.6	11.2	1.14	(0.97-1.35)	1.25	(1.07-1.45)	1.57	(1.29-1.90)	.	3.5	6.3	9.7	.
	Heart disease	9,086,446	5.2	3.8	5.3	5.0	1.19	(0.93-1.52)	1.67	(1.36-2.05)	2.97	(2.30-3.82)	.	8.4	15.4	23.8	12.1
	Kidney disease	9,113,685	3.1	2.8	3.6	3.8	1.45	(1.10-1.90)	1.77	(1.35-2.30)	2.19	(1.59-3.02)	4.6	10.3	13.2	28.1	12.2
	Stroke	9,112,714	3.7	3.2	4.0	5.4	1.29	(0.99-1.68)	1.42	(1.12-1.79)	2.05	(1.54-2.72)	.	6.7	14.7	21.4	8.4
	Violence	.	.	.	.	.	.	.	.	.	.	.	4.4	17.0	22.0	43.4	.
	Heavy drinking	8,406,205	3.0	7.2	7.9	9.8	2.29	(1.79-2.92)	2.45	(1.96-3.04)	3.18	(2.52-4.00)	11.6	16.4	22.9	51.0	.
	Illicit drug use	.	.	.	.	.	.	.	.	.	.	.	6.2	15.9	30.6	52.7	.
	OVOB	8,288,047	68.1	71.4	70.6	69.2	1.23	(1.09-1.40)	1.25	(1.11-1.40)	1.25	(1.09-1.42)	1.0	1.3	1.3	3.6	.
	Smoking	8,626,768	5.9	11.4	23.6	45.7	2.33	(1.94-2.79)	4.68	(4.01-5.45)	9.90	(8.45-11.59)	6.0	19.6	39.1	64.6	.
Oklahoma	Anxiety	.	.	.	.	.	.	.	.	.	.	.	6.1	12.6	10.4	29.1	.
	Arthritis	2,976,098	25.1	22.6	27.6	30.9	1.38	(1.15-1.65)	1.89	(1.60-2.23)	2.58	(2.16-3.09)	2.5	6.0	12.9	21.5	14.0
	Asthma	2,996,697	9.8	9.8	15.6	26.5	1.07	(0.84-1.38)	1.64	(1.31-2.05)	2.83	(2.28-3.52)	.	6.5	25.9	32.5	23.2
	Cancer	2,991,408	8.4	5.7	6.4	6.5	1.03	(0.79-1.33)	1.06	(0.83-1.34)	1.35	(1.05-1.73)	.	.	5.9	5.9	3.1
	COPD	2,993,255	5.6	7.3	7.9	14.4	1.71	(1.29-2.25)	2.13	(1.65-2.76)	3.73	(2.86-4.87)	5.1	8.9	29.1	43.0	28.2
	Depression	2,993,102	6.0	9.9	23.1	53.9	1.86	(1.44-2.42)	4.58	(3.65-5.75)	16.15	(12.77-20.43)	3.3	13.4	54.1	70.8	70.4
	Diabetes	3,004,558	14.7	11.2	12.2	11.3	1.02	(0.82-1.28)	1.21	(0.98-1.48)	1.18	(0.95-1.48)	.	.	.	.	.
	Heart disease	2,974,383	6.0	4.7	6.2	5.4	1.09	(0.80-1.48)	1.37	(1.02-1.83)	1.51	(1.05-2.17)	.	4.5	7.5	12.0	1.1
	Kidney disease	3,000,791	3.7	3.9	4.7	3.9	1.41	(0.97-2.06)	1.57	(1.14-2.17)	1.67	(1.14-2.45)	.	7.0	10.7	17.7	7.2
	Stroke	3,000,075	4.6	3.9	3.8	5.1	1.10	(0.76-1.58)	1.11	(0.80-1.55)	1.54	(1.07-2.23)	.	.	10.1	10.1	4.1
	Violence	.	.	.	.	.	.	.	.	.	.	.	4.4	17.0	22.0	43.4	.
	Heavy drinking	2,640,864	1.6	4.3	5.7	6.5	2.29	(1.51-3.47)	3.18	(2.16-4.68)	4.65	(3.20-6.77)	9.2	16.2	35.1	60.4	.
	Illicit drug use	.	.	.	.	.	.	.	.	.	.	.	6.2	15.9	30.6	52.7	.

© 2023 Peterson C et al. *JAMA Network Open.*

	OVOB	2,704,205	70.3	68.4	71.7	71.1	1.08	(0.91-1.27)	1.17	(1.00-1.38)	1.21	(1.03-1.42)	.	.	1.4	1.4	.
	Smoking	2,832,552	7.6	17.4	17.6	36.7	2.11	(1.68-2.64)	2.47	(2.00-3.05)	5.56	(4.57-6.76)	6.9	8.8	36.3	52.0	.
Oregon	Anxiety	.	.	.	.	.	.	.	.	.	.	.	6.1	12.6	10.4	29.1	.
	Arthritis	3,355,011	22.9	23.5	23.6	29.4	2.03	(1.67-2.47)	2.84	(2.35-3.42)	6.26	(4.96-7.90)	6.2	13.0	21.8	40.9	32.7
	Asthma	3,371,574	9.2	14.7	11.9	27.5	2.36	(1.82-3.05)	1.87	(1.46-2.40)	6.03	(4.60-7.91)	8.7	9.7	37.7	56.1	48.5
	Cancer	3,379,161	10.4	6.7	5.7	5.7	1.06	(0.82-1.37)	1.00	(0.79-1.28)	1.68	(1.25-2.25)	.	.	8.6	8.6	6.7
	COPD	3,365,833	3.1	6.3	5.1	9.4	3.94	(2.56-6.07)	5.58	(3.79-8.23)	13.02	(8.39-20.20)	11.9	21.1	39.3	72.3	70.3
	Depression	3,357,272	3.9	5.4	25.4	54.0	5.92	(2.59-13.54)	92.88	(43.20-199.69)	641.00	(279.21-1471.56)	2.0	30.3	65.1	97.4	97.3
	Diabetes	3,379,599	9.5	8.5	9.3	8.5	1.29	(0.97-1.72)	1.70	(1.34-2.16)	1.95	(1.45-2.63)	.	9.4	10.0	19.4	4.7
	Heart disease	3,362,909	3.6	3.1	3.5	3.6	0.88	(0.58-1.34)	1.69	(1.13-2.54)	1.78	(1.06-2.97)	.	10.7	11.0	21.8	7.5
	Kidney disease	3,375,145	2.1	2.8	2.8	3.0	2.10	(1.25-3.54)	3.17	(2.04-4.94)	3.99	(2.40-6.64)	8.9	22.2	22.0	53.2	23.6
	Stroke	3,370,149	3.0	2.4	3.2	3.4	1.06	(0.70-1.60)	1.94	(1.33-2.83)	2.96	(1.88-4.68)	.	11.8	20.2	31.9	30.2
	Violence	.	.	.	.	.	.	.	.	.	.	.	4.4	17.0	22.0	43.4	.
	Heavy drinking	3,110,817	3.1	6.3	9.0	13.7	3.12	(2.13-4.55)	3.83	(2.69-5.44)	8.05	(5.58-11.62)	10.8	21.7	39.8	72.3	.
	Illicit drug use	.	.	.	.	.	.	.	.	.	.	.	6.2	15.9	30.6	52.7	.
	OVOB	3,022,112	61.3	66.0	66.6	63.2	1.26	(1.08-1.47)	1.50	(1.30-1.74)	1.56	(1.32-1.85)	1.3	3.5	3.6	8.4	.
	Smoking	3,229,711	4.5	7.9	11.7	29.6	2.40	(1.69-3.42)	4.44	(3.27-6.03)	16.78	(12.12-23.23)	5.1	17.3	52.4	74.8	.
Pennsylvania	Anxiety	.	.	.	.	.	.	.	.	.	.	.	6.1	12.6	10.4	29.1	.
	Arthritis	10,139,479	26.5	29.3	31.4	29.3	1.66	(1.39-1.98)	2.15	(1.82-2.55)	2.82	(2.32-3.43)	4.4	8.3	10.4	23.1	19.2
	Asthma	10,165,572	9.7	12.4	16.6	26.3	1.38	(1.08-1.75)	1.73	(1.40-2.15)	2.73	(2.19-3.41)	3.9	8.4	21.9	34.1	22.9
	Cancer	10,169,511	8.7	8.9	7.9	6.1	1.40	(1.07-1.84)	1.46	(1.15-1.87)	1.44	(1.09-1.92)	4.9	6.3	5.0	16.2	6.1
	COPD	10,138,720	4.5	7.0	6.5	13.5	2.24	(1.65-3.04)	2.14	(1.58-2.89)	4.42	(3.25-5.99)	8.7	10.0	27.4	46.2	36.1
	Depression	10,150,962	6.2	11.4	20.5	49.8	1.90	(1.44-2.50)	4.39	(3.44-5.61)	13.23	(10.28-17.02)	4.2	16.0	47.3	67.5	64.3
	Diabetes	10,179,363	12.4	10.8	11.4	9.3	1.10	(0.87-1.38)	1.16	(0.94-1.44)	1.23	(0.95-1.58)	.	.	.	.	.
	Heart disease	10,111,008	5.6	5.0	4.5	4.2	1.20	(0.86-1.68)	1.17	(0.85-1.60)	1.58	(1.12-2.23)	.	.	6.4	6.4	0.5
	Kidney disease	10,153,945	2.7	2.7	4.3	3.4	1.63	(1.05-2.53)	2.45	(1.64-3.65)	2.04	(1.26-3.30)	6.4	16.4	10.6	33.3	22.6
	Stroke	10,166,875	3.8	2.6	3.5	4.3	0.91	(0.60-1.36)	1.41	(0.98-2.05)	1.78	(1.16-2.72)	.	.	10.3	10.3	3.4
	Violence	.	.	.	.	.	.	.	.	.	.	.	4.4	17.0	22.0	43.4	.
	Heavy drinking	9,391,851	3.4	6.9	8.1	9.4	1.97	(1.45-2.70)	1.78	(1.34-2.36)	2.32	(1.74-3.09)	10.3	10.2	19.4	39.9	.
	Illicit drug use	.	.	.	.	.	.	.	.	.	.	.	6.2	15.9	30.6	52.7	.
	OVOB	9,313,335	66.5	67.5	70.5	67.4	1.12	(0.95-1.30)	1.29	(1.10-1.50)	1.30	(1.11-1.53)	.	1.5	1.5	3.0	.
	Smoking	9,775,020	7.2	11.3	17.9	34.7	1.65	(1.31-2.08)	2.63	(2.13-3.26)	5.38	(4.37-6.62)	4.4	12.9	32.1	49.4	.
Rhode Island	Anxiety	.	.	.	.	.	.	.	.	.	.	.	6.1	12.6	10.4	29.1	.
	Arthritis	850,146	24.0	24.5	26.7	30.4	1.41	(1.19-1.66)	1.75	(1.46-2.09)	2.63	(2.13-3.25)	4.3	6.2	9.1	19.6	14.1
	Asthma	851,937	12.1	13.3	16.7	27.2	1.13	(0.89-1.44)	1.38	(1.09-1.76)	2.38	(1.86-3.05)	.	5.3	15.3	20.6	11.9
	Cancer	852,419	8.8	6.7	6.8	7.1	1.01	(0.80-1.27)	1.10	(0.86-1.41)	1.54	(1.17-2.01)	.	.	5.3	5.3	3.3
	COPD	852,418	5.3	5.6	8.1	11.7	1.36	(1.02-1.82)	1.88	(1.43-2.47)	3.28	(2.44-4.42)	4.8	9.8	18.7	33.3	23.2
	Depression	850,365	7.6	15.4	26.6	46.5	2.23	(1.75-2.83)	4.08	(3.24-5.14)	8.50	(6.72-10.76)	9.1	18.4	31.1	58.6	56.9
	Diabetes	853,625	11.4	10.9	9.4	9.8	1.15	(0.92-1.43)	1.08	(0.85-1.39)	1.36	(1.03-1.79)	.	.	3.6	3.6	1.2
	Heart disease	850,798	4.3	4.7	3.5	3.7	1.38	(1.02-1.88)	1.14	(0.82-1.59)	2.01	(1.38-2.92)	6.4	.	7.9	14.3	10.9
	Kidney disease	852,756	2.9	1.7	3.0	2.8	0.93	(0.59-1.48)	1.58	(0.98-2.55)	1.81	(1.06-3.09)	.	.	8.4	8.4	0.2
	Stroke	853,341	2.2	2.6	2.4	3.3	1.50	(1.03-2.19)	1.35	(0.88-2.08)	2.08	(1.31-3.29)	8.0	.	11.8	19.8	17.2
	Violence	.	.	.	.	.	.	.	.	.	.	.	4.4	17.0	22.0	43.4	.
	Heavy drinking	767,993	3.4	6.8	7.3	8.4	1.93	(1.44-2.60)	2.06	(1.52-2.78)	2.40	(1.73-3.34)	12.4	12.5	14.0	38.9	.
	Illicit drug use	.	.	.	.	.	.	.	.	.	.	.	6.2	15.9	30.6	52.7	.
	OVOB	750,448	63.2	64.4	64.6	67.1	1.09	(0.92-1.28)	1.16	(0.97-1.39)	1.37	(1.11-1.68)	.	.	1.6	1.6	.
	Smoking	791,622	6.4	11.3	14.2	28.4	1.70	(1.32-2.20)	2.14	(1.67-2.75)	4.02	(3.12-5.16)	7.6	10.9	23.2	41.7	.
South Carolina	Anxiety	.	.	.	.	.	.	.	.	.	.	.	6.1	12.6	10.4	29.1	.

© 2023 Peterson C et al. *JAMA Network Open.*

	Arthritis	4,051,660	28.9	27.0	31.7	32.1	1.21	(1.02-1.43)	1.59	(1.33-1.91)	1.95	(1.61-2.36)	2.0	5.1	6.7	13.8	7.2
	Asthma	4,060,620	9.9	12.3	16.5	23.1	1.20	(0.96-1.51)	1.60	(1.28-2.00)	2.05	(1.65-2.55)	.	7.8	14.2	22.0	14.1
	Cancer	4,066,198	8.3	7.6	8.4	7.3	1.12	(0.90-1.41)	1.38	(1.09-1.74)	1.42	(1.10-1.84)	.	5.3	5.2	10.5	9.5
	COPD	4,056,438	6.4	6.3	8.3	14.0	1.13	(0.87-1.47)	1.49	(1.13-1.96)	2.86	(2.16-3.78)	.	5.7	19.1	24.8	14.3
	Depression	4,049,267	9.2	12.7	24.7	44.2	1.47	(1.17-1.85)	3.51	(2.83-4.36)	7.01	(5.65-8.69)	3.6	15.6	32.7	52.0	47.7
	Diabetes	4,067,396	13.9	12.4	16.4	11.8	1.16	(0.94-1.42)	1.44	(1.18-1.76)	1.21	(0.95-1.54)	.	5.8	.	5.8	4.0
	Heart disease	4,041,460	5.3	4.5	4.6	5.8	1.06	(0.79-1.41)	1.09	(0.81-1.47)	2.14	(1.58-2.89)	.	.	11.7	11.7	5.3
	Kidney disease	4,057,872	3.3	2.1	4.0	4.4	0.82	(0.57-1.20)	1.40	(0.98-2.00)	1.91	(1.28-2.83)	.	.	12.2	12.2	7.3
	Stroke	4,061,284	4.0	3.9	6.2	5.4	0.99	(0.70-1.38)	1.84	(1.35-2.50)	1.93	(1.37-2.74)	.	11.6	10.4	22.0	16.1
	Violence	.	.	.	.	.	.	.	.	.	.	.	4.4	17.0	22.0	43.4	.
	Heavy drinking	3,780,021	5.4	7.6	9.1	12.8	1.33	(1.01-1.75)	1.60	(1.22-2.09)	2.27	(1.73-2.98)	4.8	8.1	17.0	29.9	.
	Illicit drug use	.	.	.	.	.	.	.	.	.	.	.	6.2	15.9	30.6	52.7	.
	OVOB	3,729,463	69.1	68.9	70.8	69.0	1.05	(0.90-1.23)	1.12	(0.95-1.31)	1.17	(0.98-1.39)	.	.	.	.	.
	Smoking	3,888,938	10.2	14.6	19.3	32.3	1.37	(1.11-1.71)	1.81	(1.46-2.24)	3.38	(2.76-4.13)	3.6	8.2	21.0	32.9	.
South Dakota	Anxiety	.	.	.	.	.	.	.	.	.	.	.	6.1	12.6	10.4	29.1	.
	Arthritis	671,024	23.1	25.5	27.4	26.7	1.68	(1.31-2.16)	2.60	(2.02-3.34)	2.87	(2.09-3.94)	4.5	14.3	8.0	26.8	18.8
	Asthma	672,219	5.1	8.9	15.7	21.5	2.07	(1.42-3.02)	3.64	(2.61-5.06)	4.99	(3.44-7.25)	6.8	27.2	24.0	57.9	52.1
	Cancer	672,507	9.5	8.2	7.0	6.5	1.05	(0.74-1.48)	1.18	(0.81-1.71)	1.87	(1.25-2.82)	.	.	6.5	6.5	2.0
	COPD	670,905	3.6	5.4	7.2	11.4	2.23	(1.44-3.46)	3.19	(2.03-5.02)	6.82	(4.12-11.28)	8.1	21.5	23.4	53.1	36.8
	Depression	670,642	3.5	7.4	18.2	52.7	2.57	(1.65-4.00)	7.27	(4.94-10.69)	41.81	(27.18-64.31)	4.3	25.0	51.6	80.9	78.8
	Diabetes	672,856	9.9	10.4	7.8	8.7	1.42	(1.04-1.92)	1.05	(0.75-1.47)	1.68	(1.11-2.54)	5.1	.	5.4	10.5	5.9
	Heart disease	668,714	4.7	4.4	4.7	4.0	1.22	(0.81-1.82)	1.75	(1.15-2.67)	1.52	(0.87-2.65)	.	12.9	.	12.9	8.4
	Kidney disease	671,961	2.9	2.5	1.9	4.0	0.97	(0.54-1.76)	0.85	(0.47-1.52)	3.47	(1.90-6.36)	.	.	18.0	18.0	12.2
	Stroke	672,649	2.4	2.3	2.6	4.0	1.49	(0.87-2.57)	2.40	(1.50-3.84)	3.33	(1.71-6.46)	.	17.9	12.6	30.5	20.7
	Violence	.	.	.	.	.	.	.	.	.	.	.	4.4	17.0	22.0	43.4	.
	Heavy drinking	639,758	3.6	7.7	8.2	10.8	1.88	(1.14-3.12)	2.58	(1.62-4.10)	4.04	(2.48-6.61)	7.4	21.0	18.8	47.3	.
	Illicit drug use	.	.	.	.	.	.	.	.	.	.	.	6.2	15.9	30.6	52.7	.
	OVOB	606,654	70.2	70.5	71.0	68.9	1.13	(0.89-1.44)	1.18	(0.94-1.46)	1.33	(1.00-1.78)	.	.	1.3	1.3	.
	Smoking	654,619	7.3	10.7	23.8	42.2	1.69	(1.18-2.42)	3.88	(2.90-5.19)	9.04	(6.53-12.53)	3.9	24.2	28.6	56.7	.
Tennessee	Anxiety	.	.	.	.	.	.	.	.	.	.	.	6.1	12.6	10.4	29.1	.
	Arthritis	5,323,134	29.7	26.2	30.3	34.2	1.25	(1.04-1.51)	1.83	(1.51-2.21)	2.51	(2.05-3.07)	1.8	5.7	11.8	19.4	11.1
	Asthma	5,336,384	9.7	11.4	15.2	25.6	1.36	(1.06-1.76)	1.82	(1.44-2.30)	3.13	(2.48-3.95)	3.4	8.6	28.6	40.6	31.8
	Cancer	5,347,116	9.2	7.9	6.3	7.2	1.12	(0.85-1.47)	1.09	(0.83-1.43)	1.50	(1.13-1.99)	.	.	8.5	8.5	2.9
	COPD	5,342,003	6.8	6.8	9.6	15.1	1.33	(0.97-1.84)	2.03	(1.51-2.73)	3.11	(2.29-4.23)	.	8.9	24.4	33.3	21.5
	Depression	5,335,223	7.4	9.4	25.0	55.0	1.82	(1.39-2.37)	5.06	(3.95-6.48)	16.92	(13.09-21.86)	3.1	15.4	52.0	70.4	68.2
	Diabetes	5,350,278	16.0	14.7	13.7	11.0	1.04	(0.83-1.31)	1.33	(1.05-1.67)	1.12	(0.89-1.42)	.	3.7	.	3.7	1.2
	Heart disease	5,326,212	5.5	4.6	5.2	5.0	1.08	(0.76-1.54)	1.59	(1.14-2.22)	2.14	(1.50-3.04)	.	6.7	13.9	20.6	8.6
	Kidney disease	5,347,577	2.9	4.0	3.3	4.1	1.85	(1.23-2.79)	1.65	(1.08-2.51)	2.03	(1.25-3.30)	8.7	7.2	14.8	30.7	19.2
	Stroke	5,349,853	4.6	3.7	4.9	5.1	1.14	(0.79-1.64)	1.82	(1.28-2.60)	1.89	(1.32-2.70)	.	10.2	14.0	24.2	13.9
	Violence	.	.	.	.	.	.	.	.	.	.	.	4.4	17.0	22.0	43.4	.
	Heavy drinking	4,839,619	2.4	4.6	7.5	8.5	1.78	(1.17-2.71)	2.65	(1.79-3.94)	3.03	(2.03-4.53)	7.2	16.5	29.1	52.8	.
	Illicit drug use	.	.	.	.	.	.	.	.	.	.	.	6.2	15.9	30.6	52.7	.
	OVOB	4,810,681	68.0	71.0	70.8	70.3	1.22	(1.02-1.45)	1.30	(1.09-1.55)	1.41	(1.17-1.69)	0.8	1.4	2.4	4.7	.
	Smoking	5,002,565	8.1	14.0	19.9	35.9	2.02	(1.57-2.60)	2.72	(2.14-3.44)	5.94	(4.73-7.46)	6.1	11.0	36.6	53.7	.
Texas	Anxiety	.	.	.	.	.	.	.	.	.	.	.	6.1	12.6	10.4	29.1	.
	Arthritis	21,747,324	19.7	19.1	21.6	25.1	1.43	(1.15-1.78)	2.09	(1.71-2.55)	2.96	(2.35-3.73)	2.9	9.8	13.1	25.8	18.3
	Asthma	21,802,346	8.4	7.0	11.8	25.5	1.04	(0.76-1.42)	1.59	(1.23-2.06)	3.99	(3.02-5.28)	.	7.7	30.5	38.2	31.4
	Cancer	21,831,578	6.4	5.9	4.8	5.8	1.21	(0.88-1.65)	0.97	(0.73-1.27)	1.67	(1.23-2.27)	.	.	8.9	8.9	6.4

© 2023 Peterson C et al. *JAMA Network Open.*

	COPD	21,801,986	3.1	4.1	5.5	9.4	1.85	(1.24-2.75)	2.98	(2.11-4.20)	5.33	(3.69-7.70)	5.1	16.7	31.1	52.9	42.8
	Depression	21,812,818	5.5	7.5	15.7	47.9	2.35	(1.71-3.23)	5.02	(3.82-6.59)	29.34	(21.93-39.26)	4.0	16.3	58.2	78.5	75.8
	Diabetes	21,840,826	15.5	11.7	11.1	11.3	0.94	(0.71-1.24)	1.15	(0.90-1.48)	1.32	(0.98-1.77)	.	.	.	.	.
	Heart disease	21,693,894	4.1	2.4	2.9	2.8	0.90	(0.61-1.31)	1.44	(1.02-2.01)	2.07	(1.35-3.15)	.	6.6	10.1	16.8	9.0
	Kidney disease	21,781,080	3.1	3.0	3.1	2.8	1.33	(0.84-2.10)	2.06	(1.35-3.14)	2.19	(1.33-3.61)	.	13.9	12.6	26.5	21.5
	Stroke	21,823,998	3.3	2.6	2.8	4.3	1.22	(0.80-1.88)	1.52	(1.05-2.19)	2.16	(1.46-3.21)	.	7.0	14.9	21.9	14.8
	Violence	.	.	.	.	.	.	.	.	.	.	.	4.4	17.0	22.0	43.4	.
	Heavy drinking	19,597,514	2.3	5.2	8.0	11.1	2.34	(1.58-3.47)	3.24	(2.23-4.71)	5.47	(3.72-8.05)	8.1	21.2	33.8	63.1	.
	Illicit drug use	.	.	.	.	.	.	.	.	.	.	.	6.2	15.9	30.6	52.7	.
	OVOB	19,259,548	68.0	70.8	72.0	70.1	1.08	(0.90-1.30)	1.26	(1.07-1.50)	1.15	(0.96-1.39)	.	1.5	.	1.5	.
	Smoking	20,497,872	5.7	12.8	13.6	28.8	2.44	(1.86-3.22)	3.09	(2.42-3.95)	6.83	(5.39-8.66)	8.3	15.5	35.0	58.8	.
Utah	Anxiety	.	.	.	.	.	.	.	.	.	.	.	6.1	12.6	10.4	29.1	.
	Arthritis	2,299,268	20.7	19.0	24.0	25.1	1.46	(1.27-1.67)	2.21	(1.96-2.48)	3.72	(3.22-4.28)	3.1	12.4	15.7	31.2	23.2
	Asthma	2,301,482	6.6	14.4	17.2	28.0	2.15	(1.79-2.58)	2.57	(2.20-3.01)	4.93	(4.17-5.83)	7.0	17.9	31.4	56.3	49.3
	Cancer	2,306,509	6.5	4.9	5.0	5.0	1.04	(0.85-1.28)	1.15	(0.96-1.37)	1.56	(1.25-1.95)	.	.	7.4	7.4	5.9
	COPD	2,301,745	1.9	3.4	4.0	7.4	2.23	(1.66-2.98)	2.55	(1.95-3.33)	5.78	(4.31-7.74)	7.5	15.8	34.6	57.9	39.3
	Depression	2,296,655	5.7	10.6	23.4	53.9	2.23	(1.83-2.72)	4.63	(3.93-5.45)	19.44	(16.34-23.14)	4.1	20.9	49.4	74.4	71.7
	Diabetes	2,308,523	8.6	8.7	7.9	6.9	1.13	(0.94-1.36)	1.20	(1.01-1.41)	1.49	(1.22-1.83)	.	3.3	5.4	8.7	1.8
	Heart disease	2,297,707	2.9	2.9	2.4	2.2	1.26	(0.96-1.66)	1.37	(1.04-1.81)	1.81	(1.28-2.56)	.	7.0	8.4	15.4	.
	Kidney disease	2,303,937	2.4	2.4	2.8	2.6	1.52	(1.12-2.06)	2.04	(1.55-2.69)	2.49	(1.77-3.50)	5.2	16.2	14.6	35.9	23.2
	Stroke	2,306,755	2.0	2.0	2.2	2.5	1.30	(0.94-1.81)	1.64	(1.25-2.14)	2.38	(1.70-3.35)	.	10.6	15.0	25.6	19.6
	Violence	.	.	.	.	.	.	.	.	.	.	.	4.4	17.0	22.0	43.4	.
	Heavy drinking	2,193,572	1.4	2.7	6.7	6.9	1.74	(1.16-2.61)	4.14	(3.03-5.67)	4.96	(3.56-6.92)	4.0	30.8	30.9	65.7	.
	Illicit drug use	.	.	.	.	.	.	.	.	.	.	.	6.2	15.9	30.6	52.7	.
	OVOB	2,094,582	60.8	65.0	66.1	63.3	1.34	(1.20-1.51)	1.43	(1.30-1.58)	1.64	(1.46-1.84)	1.5	3.5	3.8	8.8	.
	Smoking	2,231,956	1.9	4.4	7.9	18.4	1.72	(1.23-2.41)	3.17	(2.40-4.19)	8.05	(6.05-10.70)	3.2	17.1	43.9	64.1	.
Vermont	Anxiety	.	.	.	.	.	.	.	.	.	.	.	6.1	12.6	10.4	29.1	.
	Arthritis	508,254	24.8	25.9	29.5	27.7	2.21	(1.84-2.64)	2.72	(2.29-3.24)	4.29	(3.41-5.39)	7.5	12.0	14.7	34.2	26.3
	Asthma	508,858	10.6	11.8	16.3	28.7	1.33	(1.00-1.77)	2.29	(1.78-2.96)	4.41	(3.28-5.92)	2.9	13.3	30.0	46.2	46.8
	Cancer	509,638	8.0	7.7	5.7	6.2	1.60	(1.25-2.05)	1.14	(0.88-1.47)	1.84	(1.30-2.61)	7.0	.	8.9	15.9	7.2
	COPD	508,275	3.5	5.5	7.7	10.7	3.66	(2.59-5.17)	4.98	(3.56-6.96)	9.98	(6.66-14.97)	10.4	20.4	35.5	66.3	56.6
	Depression	508,045	2.9	8.6	26.8	60.3	9.44	(5.27-16.90)	64.36	(36.75-112.72)	582.36	(310.39-1092.61)	4.4	26.2	64.8	95.3	95.2
	Diabetes	509,856	8.1	8.8	9.3	7.8	1.53	(1.18-1.99)	1.75	(1.36-2.25)	1.94	(1.39-2.71)	6.0	9.8	9.7	25.5	18.3
	Heart disease	507,945	4.2	4.5	3.9	4.1	2.07	(1.46-2.94)	2.08	(1.47-2.94)	3.89	(2.43-6.22)	10.1	10.7	16.7	37.5	14.5
	Kidney disease	509,497	2.1	2.4	2.6	2.2	1.40	(0.83-2.37)	2.06	(1.21-3.52)	2.39	(1.07-5.31)	.	13.0	13.5	26.5	13.4
	Stroke	509,378	3.0	2.7	2.8	2.9	1.19	(0.77-1.83)	1.81	(1.21-2.72)	2.05	(1.26-3.33)	.	9.8	10.9	20.7	4.6
	Violence	.	.	.	.	.	.	.	.	.	.	.	4.4	17.0	22.0	43.4	.
	Heavy drinking	472,021	4.0	14.8	13.9	12.5	4.62	(3.38-6.32)	4.61	(3.38-6.30)	4.64	(3.25-6.61)	19.3	24.9	23.2	67.5	.
	Illicit drug use	.	.	.	.	.	.	.	.	.	.	.	6.2	15.9	30.6	52.7	.
	OVOB	458,657	58.5	64.3	61.4	62.1	1.40	(1.19-1.66)	1.46	(1.24-1.72)	1.69	(1.38-2.08)	2.1	2.8	3.4	8.4	.
	Smoking	482,109	3.0	9.9	14.7	35.6	3.48	(2.47-4.91)	7.42	(5.37-10.23)	27.55	(19.43-39.06)	6.9	19.1	55.0	80.9	.
Virginia	Anxiety	.	.	.	.	.	.	.	.	.	.	.	6.1	12.6	10.4	29.1	.
	Arthritis	6,686,598	24.7	25.0	26.1	29.9	1.21	(1.07-1.38)	1.51	(1.31-1.73)	2.37	(2.00-2.80)	2.2	4.8	7.6	14.6	10.0
	Asthma	6,701,348	8.8	13.1	16.1	24.2	1.47	(1.23-1.76)	1.83	(1.54-2.17)	2.69	(2.24-3.24)	6.1	10.6	16.5	33.2	25.2
	Cancer	6,705,466	7.9	6.9	6.8	6.8	1.06	(0.89-1.28)	1.17	(0.96-1.42)	1.61	(1.27-2.03)	.	.	5.6	5.6	3.7
	COPD	6,689,611	4.2	5.4	7.2	10.4	1.48	(1.19-1.84)	2.15	(1.73-2.67)	3.24	(2.56-4.10)	5.7	12.4	17.2	35.3	24.6
	Depression	6,696,495	6.5	12.6	21.0	44.5	1.98	(1.65-2.38)	3.54	(2.99-4.19)	9.54	(8.00-11.39)	7.1	17.2	34.5	58.8	56.5
	Diabetes	6,711,522	12.3	11.0	10.2	10.0	0.95	(0.80-1.11)	0.99	(0.83-1.17)	1.20	(0.98-1.48)	.	.	.	.	.

© 2023 Peterson C et al. *JAMA Network Open.*

	Heart disease	6,678,541	3.7	4.0	3.5	3.0	1.17	(0.92-1.49)	1.28	(1.00-1.65)	1.41	(1.01-1.97)	.	.	3.5	3.5	.
	Kidney disease	6,697,488	2.6	2.9	2.5	2.9	1.23	(0.91-1.66)	1.27	(0.94-1.72)	1.77	(1.23-2.56)	.	.	7.7	7.7	6.3
	Stroke	6,710,621	3.2	3.0	2.6	4.1	1.05	(0.81-1.37)	1.01	(0.75-1.34)	1.74	(1.27-2.38)	.	.	8.0	8.0	3.6
	Violence	.	.	.	.	.	.	.	.	.	.	.	4.4	17.0	22.0	43.4	.
	Heavy drinking	6,208,497	3.7	5.7	6.4	8.8	1.43	(1.12-1.81)	1.57	(1.25-1.99)	2.24	(1.73-2.89)	6.7	9.1	14.4	30.2	.
	Illicit drug use	.	.	.	.	.	.	.	.	.	.	.	6.2	15.9	30.6	52.7	.
	OVOB	6,002,491	64.1	68.1	66.6	71.7	1.18	(1.05-1.33)	1.09	(0.97-1.24)	1.51	(1.31-1.75)	1.0	.	1.9	3.0	.
	Smoking	6,345,032	8.9	11.7	15.4	26.7	1.29	(1.08-1.55)	1.66	(1.40-1.97)	3.09	(2.58-3.69)	3.0	7.6	16.9	27.5	.
Washington	Anxiety	.	.	.	.	.	.	.	.	.	.	.	6.1	12.6	10.4	29.1	.
	Arthritis	6,005,577	24.2	24.1	24.7	25.6	2.35	(2.06-2.68)	3.01	(2.64-3.42)	5.97	(4.96-7.18)	8.1	15.3	18.7	42.1	35.0
	Asthma	6,009,355	8.3	14.6	15.9	25.5	2.49	(2.07-2.99)	2.62	(2.19-3.13)	6.90	(5.54-8.59)	9.9	17.2	34.6	61.7	53.3
	Cancer	6,017,436	10.1	7.2	6.2	6.6	1.07	(0.89-1.27)	1.08	(0.92-1.28)	1.72	(1.36-2.17)	.	.	9.1	9.1	4.0
	COPD	6,007,008	2.7	5.2	4.9	8.9	5.10	(3.85-6.76)	6.23	(4.75-8.16)	18.92	(13.47-26.58)	14.2	22.3	41.7	78.2	75.4
	Depression	6,000,464	4.1	7.3	32.7	51.8	5.60	(3.83-8.19)	65.42	(45.21-94.67)	468.23	(304.88-719.10)	3.1	37.8	55.4	96.3	96.6
	Diabetes	6,021,761	10.0	9.0	9.2	8.4	1.09	(0.92-1.30)	1.54	(1.31-1.82)	1.65	(1.30-2.09)	.	8.3	8.1	16.4	7.3
	Heart disease	5,999,879	4.8	3.6	3.6	2.9	1.53	(1.19-1.96)	2.05	(1.63-2.57)	2.72	(1.92-3.87)	6.4	14.0	12.4	32.8	15.4
	Kidney disease	6,011,518	2.5	3.0	2.4	3.3	1.61	(1.18-2.20)	2.03	(1.55-2.65)	3.74	(2.56-5.46)	6.7	13.7	21.5	41.9	22.0
	Stroke	6,017,950	2.9	2.6	3.1	3.2	1.35	(0.99-1.84)	2.21	(1.68-2.91)	3.21	(2.13-4.84)	.	15.1	19.7	34.8	23.0
	Violence	.	.	.	.	.	.	.	.	.	.	.	4.4	17.0	22.0	43.4	.
	Heavy drinking	5,457,062	2.9	5.6	7.1	9.8	3.10	(2.40-4.00)	3.99	(3.16-5.04)	9.20	(7.05-12.00)	11.6	26.0	35.8	73.4	.
	Illicit drug use	.	.	.	.	.	.	.	.	.	.	.	6.2	15.9	30.6	52.7	.
	OVOB	5,266,864	62.2	67.9	66.8	60.1	1.25	(1.12-1.40)	1.43	(1.29-1.59)	1.55	(1.35-1.77)	1.1	2.8	2.9	6.8	.
	Smoking	5,580,969	4.2	7.4	12.2	24.6	1.89	(1.45-2.46)	3.95	(3.10-5.03)	12.01	(9.02-15.99)	4.0	19.5	44.2	67.6	.
West Virginia	Anxiety	.	.	.	.	.	.	.	.	.	.	.	6.1	12.6	10.4	29.1	.
	Arthritis	1,419,909	38.5	34.3	41.2	44.4	1.34	(1.13-1.58)	1.86	(1.59-2.18)	2.82	(2.37-3.37)	1.7	4.8	10.1	16.6	10.4
	Asthma	1,422,410	9.9	13.8	21.0	27.8	1.47	(1.15-1.88)	2.38	(1.94-2.91)	3.01	(2.45-3.69)	3.4	12.0	24.0	39.4	27.7
	Cancer	1,423,867	9.7	8.1	6.6	9.4	1.12	(0.88-1.42)	0.83	(0.66-1.05)	1.44	(1.12-1.85)	.	.	7.1	7.1	1.9
	COPD	1,416,399	8.7	11.3	15.3	20.3	1.88	(1.49-2.38)	2.45	(1.99-3.02)	3.97	(3.18-4.97)	4.8	10.8	24.5	40.2	29.2
	Depression	1,418,066	8.2	15.5	33.8	64.5	2.52	(2.00-3.18)	7.69	(6.25-9.44)	26.70	(21.07-33.84)	4.2	18.0	50.7	73.0	71.6
	Diabetes	1,425,824	17.3	14.6	15.8	13.6	1.05	(0.86-1.28)	1.20	(1.00-1.44)	1.21	(0.98-1.49)	.	.	.	.	.
	Heart disease	1,412,454	8.2	6.3	7.1	6.9	1.12	(0.86-1.46)	1.15	(0.90-1.47)	1.66	(1.25-2.20)	.	.	8.4	8.4	1.9
	Kidney disease	1,422,733	4.0	4.1	5.1	4.7	1.33	(0.96-1.84)	1.72	(1.30-2.27)	1.77	(1.27-2.46)	.	8.3	10.4	18.7	10.7
	Stroke	1,424,300	4.9	3.4	4.9	5.9	0.84	(0.59-1.19)	1.28	(0.96-1.71)	1.76	(1.27-2.43)	.	.	11.8	11.8	3.0
	Violence	.	.	.	.	.	.	.	.	.	.	.	4.4	17.0	22.0	43.4	.
	Heavy drinking	1,366,561	2.6	4.9	5.9	8.1	1.86	(1.22-2.84)	2.23	(1.55-3.20)	3.84	(2.70-5.46)	7.0	12.0	30.8	49.8	.
	Illicit drug use	.	.	.	.	.	.	.	.	.	.	.	6.2	15.9	30.6	52.7	.
	OVOB	1,333,723	71.8	71.5	71.3	71.4	1.04	(0.87-1.24)	1.09	(0.93-1.28)	1.24	(1.05-1.48)	.	.	1.3	1.3	.
	Smoking	1,392,157	10.9	20.5	24.6	45.5	2.21	(1.79-2.73)	2.69	(2.23-3.25)	6.23	(5.19-7.47)	6.0	10.6	34.0	50.5	.
Wisconsin	Anxiety	.	.	.	.	.	.	.	.	.	.	.	6.1	12.6	10.4	29.1	.
	Arthritis	4,557,037	26.0	22.8	29.0	27.5	1.02	(0.86-1.21)	1.59	(1.33-1.90)	1.74	(1.40-2.17)	.	5.6	5.5	11.1	5.1
	Asthma	4,557,097	8.7	10.7	16.4	24.8	1.31	(1.02-1.69)	1.93	(1.52-2.44)	2.73	(2.12-3.52)	3.7	11.0	19.0	33.7	25.4
	Cancer	4,565,816	8.8	7.3	6.7	5.6	1.09	(0.85-1.39)	1.05	(0.80-1.37)	1.27	(0.93-1.75)	.	.	.	.	.
	COPD	4,560,474	4.0	4.8	5.9	8.8	1.56	(1.10-2.21)	1.97	(1.40-2.78)	3.38	(2.30-4.97)	6.5	10.4	21.1	37.9	23.4
	Depression	4,557,712	8.8	14.0	26.6	43.3	1.72	(1.34-2.21)	3.36	(2.67-4.24)	6.37	(5.00-8.11)	5.6	16.8	29.3	51.7	48.9
	Diabetes	4,569,717	9.5	8.7	9.4	7.1	1.08	(0.84-1.39)	1.20	(0.94-1.55)	1.22	(0.90-1.65)	.	.	.	.	.
	Heart disease	4,548,350	3.7	3.5	4.4	3.0	1.31	(0.95-1.81)	1.92	(1.38-2.68)	1.92	(1.27-2.90)	.	11.7	7.3	19.0	13.8
	Kidney disease	4,562,969	2.2	3.0	2.8	3.5	1.64	(0.98-2.76)	1.53	(0.96-2.44)	2.27	(1.33-3.89)	.	.	13.2	13.2	11.2
	Stroke	4,563,371	2.4	2.2	2.7	3.1	1.23	(0.80-1.88)	1.76	(1.10-2.80)	2.46	(1.53-3.96)	.	9.1	15.1	24.3	17.2

© 2023 Peterson C et al. *JAMA Network Open.*

	Violence	.	.	.	.	.	.	.	.	.	.	.	4.4	17.0	22.0	43.4	.
	Heavy drinking	4,274,977	6.2	9.8	8.6	13.8	1.71	(1.29-2.25)	1.36	(1.01-1.81)	2.53	(1.89-3.38)	9.1	4.6	17.0	30.8	.
	Illicit drug use	.	.	.	.	.	.	.	.	.	.	.	6.2	15.9	30.6	52.7	.
	OVOB	4,171,694	68.6	66.3	71.3	70.3	0.95	(0.80-1.12)	1.24	(1.04-1.48)	1.20	(0.98-1.47)	.	1.2	.	1.2	.
	Smoking	4,359,115	7.9	13.3	16.4	30.5	1.95	(1.51-2.50)	2.51	(1.97-3.20)	4.56	(3.59-5.80)	7.8	12.7	25.2	45.7	.
Wyoming	Anxiety	.	.	.	.	.	.	.	.	.	.	.	6.1	12.6	10.4	29.1	.
	Arthritis	441,652	21.6	24.5	23.5	28.7	1.64	(1.34-2.00)	2.01	(1.64-2.45)	3.44	(2.75-4.32)	5.2	7.8	17.0	30.0	23.0
	Asthma	442,722	8.8	11.8	14.2	23.9	1.34	(1.00-1.78)	1.43	(1.09-1.88)	2.75	(2.09-3.62)	3.4	5.5	25.1	34.0	21.7
	Cancer	442,284	8.9	7.1	5.3	7.4	0.95	(0.73-1.24)	0.79	(0.58-1.07)	1.37	(1.00-1.89)	.	.	.	.	.
	COPD	441,925	4.3	6.0	6.5	10.4	1.74	(1.25-2.44)	2.42	(1.75-3.35)	3.68	(2.59-5.23)	6.2	11.3	26.3	43.8	27.5
	Depression	442,028	4.5	8.5	16.8	46.7	2.57	(1.76-3.75)	5.28	(3.75-7.43)	21.89	(15.43-31.07)	5.0	15.0	59.0	79.1	76.0
	Diabetes	443,244	8.6	7.6	8.3	7.6	1.13	(0.86-1.48)	1.49	(1.14-1.96)	1.73	(1.28-2.33)	.	5.9	9.0	14.9	4.6
	Heart disease	441,104	3.2	3.4	4.4	3.4	1.38	(0.96-2.00)	2.93	(2.02-4.25)	2.89	(1.84-4.53)	.	17.8	15.6	33.3	23.8
	Kidney disease	442,436	1.7	2.1	2.3	3.6	1.72	(1.04-2.84)	2.47	(1.52-3.99)	4.78	(2.91-7.85)	6.7	12.4	31.3	50.4	38.8
	Stroke	442,720	3.3	2.4	2.6	4.1	1.12	(0.70-1.78)	1.61	(1.01-2.56)	2.18	(1.35-3.52)	.	7.4	17.5	24.9	10.3
	Violence	.	.	.	.	.	.	.	.	.	.	.	4.4	17.0	22.0	43.4	.
	Heavy drinking	419,229	3.2	6.3	6.1	11.1	2.18	(1.41-3.38)	2.32	(1.51-3.55)	4.00	(2.65-6.02)	9.3	12.7	32.9	54.8	.
	Illicit drug use	.	.	.	.	.	.	.	.	.	.	.	6.2	15.9	30.6	52.7	.
	OVOB	408,489	63.4	66.2	71.9	64.8	1.14	(0.95-1.37)	1.63	(1.34-1.97)	1.38	(1.13-1.69)	.	3.0	2.5	5.5	.
	Smoking	426,846	7.7	11.1	16.8	39.7	1.56	(1.18-2.08)	2.34	(1.78-3.09)	6.13	(4.71-7.98)	3.6	9.8	38.9	52.3	.

Abbreviations: ACE = Adverse childhood experience, BRFSS = Behavioral Risk Factor Surveillance System, COPD = Chronic obstructive pulmonary disorder, OVOB = Overweight or obese, PAF = Population attributable fraction.

© 2023 Peterson C et al. *JAMA Network Open.*

eTable 4. State-level annual ACE DALY economic burden among adults by health condition, $ millions 2019 USD

State	Anxiety	Arthritis	Asthma	Cancer	COPD	Depression	Diabetes	Heart disease	Kidney disease	Stroke	Violence	Heavy drinking	Illicit drug use	OVOB	Smoking	Total
United States	$261,403	$282,037	$229,150	$773,668	$1,337,062	$960,144	$158,606	$1,182,499	$241,959	$423,388	$227,219	$1,470,625	$1,997,078	$225,282	$4,156,191	$13,926,313
Alabama	$3,966	$2,308	$1,342	.	$11,269	$8,196	.	$14,787	.	$3,431	$5,535	$16,627	$37,757	$3,153	$46,290	$154,661
Alaska	$613	$966	$594	$3,256	$2,900	$2,958	$1,364	$2,343	$600	$1,050	$478	$4,593	$5,697	$140	$8,030	$35,582
Arizona	$5,669	$5,177	$6,479	$19,985	$29,376	$23,069	$5,802	$20,557	$1,619	$3,783	$5,384	$35,973	$50,138	.	$76,409	$289,421
Arkansas	$2,396	$2,967	$2,559	$8,443	$22,286	$13,422	$2,516	$23,767	$2,939	$8,919	$2,795	$20,650	$17,490	$3,664	$77,365	$212,180
California	$32,563	$44,294	$27,169	$82,375	$158,042	$109,141	$8,743	$177,475	$32,560	$28,787	$28,357	$224,046	$178,306	$31,645	$324,935	$1,488,438
Colorado	$4,354	$7,505	$4,777	$13,418	$30,569	$22,402	$4,715	$23,084	$4,750	$11,474	$2,617	$38,603	$31,287	$5,789	$71,281	$276,626
Connecticut	$3,024	$4,614	$3,501	$7,020	$16,539	$10,754	$2,805	$13,949	$2,945	$6,908	$1,584	$17,869	$22,985	$4,062	$52,255	$170,813
Delaware	$781	$891	$614	$4,203	$3,932	$2,931	.	$3,830	$1,069	$1,463	$588	$3,820	$7,455	$1,228	$14,644	$47,450
DC	$596	$321	$183	.	$507	$1,593	$405	$2,065	.	.	$1,851	$1,993	$4,403	.	$4,045	$17,961
Florida	$17,139	$15,024	$12,089	$31,017	$78,337	$54,274	.	$114,829	$4,539	$18,101	$16,003	$84,798	$136,898	.	$203,450	$786,499
Georgia	$8,525	$7,544	$6,302	$31,155	$37,917	$30,558	.	.	$5,576	.	$9,415	$46,987	$56,797	$7,390	$123,646	$371,811
Hawaii	$1,203	$1,063	$941	.	$3,331	$3,805	$1,298	$2,156	$926	.	$527	$5,814	$5,486	$1,753	$12,466	$40,771
Idaho	$1,306	$1,630	$951	$2,607	$6,677	$5,380	$1,700	$4,571	$1,328	$2,792	$494	$6,808	$8,881	$1,508	$17,673	$64,304
Illinois	$10,521	$14,557	$12,033	.	$77,448	$39,998	$8,319	$66,347	$19,711	$10,104	$11,293	$63,841	$68,962	$18,383	$219,386	$640,902
Indiana	$5,245	$5,902	$5,473	$11,405	$32,692	$21,832	$5,174	$33,860	$5,737	$10,959	$4,718	$27,082	$45,206	$5,848	$107,434	$328,567
Iowa	$2,428	$1,965	$1,211	$3,645	$9,279	$6,034	$1,300	$3,706	$387	.	$886	$5,751	$11,634	$2,382	$29,104	$79,712
Kansas	$2,296	$2,317	$1,978	$6,112	$13,526	$8,962	$2,105	$11,219	$1,624	$1,540	$1,608	$12,100	$13,851	$2,821	$39,870	$121,927
Kentucky	$3,514	$3,965	$3,355	$10,422	$24,553	$15,443	$4,082	$30,658	$4,293	$9,077	$2,787	$23,147	$49,176	.	$107,296	$291,767
Louisiana	$3,587	$4,518	$2,917	$13,312	$23,977	$15,432	$2,819	$24,188	$3,479	$4,917	$6,163	$30,435	$35,799	.	$98,939	$270,483
Maine	$1,095	$2,146	$1,395	$3,934	$10,281	$5,268	$2,145	$7,086	$1,463	$2,714	$354	$9,075	$9,267	$2,207	$33,369	$91,798
Maryland	$5,029	$6,335	$4,401	.	$20,803	$18,727	$2,093	$20,828	$3,733	$4,865	$7,121	$30,898	$28,208	$4,371	$73,843	$231,254
Massachusetts	$5,530	$6,867	$5,970	$13,398	$29,362	$22,205	$5,024	$22,338	$5,571	$7,145	$2,513	$31,595	$51,497	$4,214	$92,919	$306,150
Michigan	$7,699	$8,262	$6,975	$12,031	$39,631	$25,613	$7,279	$31,142	$3,796	$14,538	$7,474	$33,397	$69,980	$9,355	$133,802	$410,973
Minnesota	$4,430	$6,189	$5,164	$6,264	$27,606	$19,854	$3,567	$16,631	$4,004	$9,534	$1,852	$27,854	$20,172	$6,179	$83,033	$242,331
Mississippi	$2,366	$891	$1,087	$5,272	$8,411	$4,451	$1,124	$3,839	$1,559	$2,134	$3,659	$10,181	$19,976	$852	$33,333	$99,134
Missouri	$4,944	$3,444	$2,636	$15,451	$19,946	$11,650	.	$18,947	$1,633	$12,172	$5,626	$13,511	$41,350	$8,760	$72,728	$232,799
Montana	$811	$1,159	$557	$2,504	$5,694	$3,331	.	$3,431	$575	$844	$392	$4,994	$6,045	$575	$13,853	$44,767
Nebraska	$1,476	$2,290	$1,934	$4,715	$12,687	$6,451	$2,483	$7,794	$1,670	$3,321	$804	$8,833	$6,001	$1,758	$33,393	$95,609
Nevada	$2,592	$2,500	$2,829	.	$12,290	$10,390	.	.	$2,383	$2,247	$2,404	$20,399	$23,247	$2,890	$44,525	$128,696
New Hampshire	$1,106	$1,867	$1,244	$6,354	$8,313	$5,548	$1,930	$4,431	$1,250	$1,461	$352	$8,836	$11,355	$1,550	$27,034	$82,631
New Jersey	$7,331	$7,772	$7,226	.	$29,168	$23,597	$3,646	$9,588	$11,210	$4,116	$5,211	$37,427	$52,008	$4,999	$103,862	$307,160
New Mexico	$1,704	$2,042	$1,924	.	$10,577	$8,044	$1,031	$5,217	$1,162	$3,684	$1,746	$14,166	$19,036	.	$25,413	$95,744
New York	$16,257	$20,154	$18,639	.	$61,734	$65,576	$8,579	$62,127	$16,763	$20,296	$11,021	$87,182	$99,430	$7,909	$268,470	$764,139
North Carolina	$8,257	$11,782	$9,820	.	$63,097	$32,704	$11,302	$41,896	$7,462	$14,935	$7,848	$53,848	$64,658	$11,152	$207,074	$545,834
North Dakota	$550	$420	$328	$818	$1,543	$1,312	.	$602	.	.	$229	$1,405	$2,523	$592	$4,891	$15,213
Ohio	$9,108	$13,019	$11,289	$40,911	$67,506	$39,054	$9,528	$56,433	$9,439	$18,395	$7,547	$49,749	$104,402	$10,492	$225,424	$672,295
Oklahoma	$3,040	$2,730	$2,042	$6,762	$18,524	$12,091	.	$11,046	$1,967	$2,921	$3,052	$21,562	$32,875	$1,382	$65,233	$185,228
Oregon	$2,467	$5,907	$3,816	$9,694	$25,739	$16,601	$5,389	$10,944	$4,687	$8,515	$1,476	$28,687	$24,894	$6,327	$67,873	$223,016
Pennsylvania	$10,409	$11,952	$7,666	$64,496	$54,843	$31,822	.	$15,350	$12,269	$9,412	$8,534	$43,555	$110,820	$8,910	$171,481	$561,519
Rhode Island	$864	$747	$389	$1,595	$2,871	$2,776	$246	$2,567	$190	$1,088	$413	$3,590	$8,296	$335	$10,410	$36,379
South Carolina	$4,022	$2,195	$1,800	$16,335	$12,781	$10,850	$2,362	$11,249	$1,898	$9,413	$4,635	$14,299	$34,902	.	$48,452	$175,194
South Dakota	$655	$764	$687	$1,536	$3,943	$2,185	$599	$1,891	$342	$1,620	$331	$3,457	$2,917	$225	$11,452	$32,604
Tennessee	$5,349	$4,240	$4,027	$18,001	$23,570	$20,136	$1,958	$31,332	$5,596	$13,227	$5,675	$31,788	$61,070	$8,004	$117,136	$351,108
Texas	$22,244	$19,773	$15,311	$54,915	$101,304	$79,114	.	$68,379	$17,828	$35,983	$20,634	$134,419	$132,841	$8,025	$308,823	$1,019,593
Utah	$2,315	$2,482	$2,323	$3,702	$9,205	$10,992	$1,453	$4,167	$1,919	$3,492	$968	$12,478	$20,114	$3,633	$19,320	$98,563
Vermont	$514	$857	$479	$3,081	$4,146	$2,506	$1,097	$4,024	$333	$749	$179	$3,846	$3,464	$1,088	$12,910	$39,273

© 2023 Peterson C et al. *JAMA Network Open.*

Virginia	$7,063	$4,091	$4,750	$12,527	$22,841	$20,224	.	$4,327	$1,731	$4,295	$5,477	$21,419	$42,951	$5,079	$52,039	$208,812
Washington	$5,845	$10,750	$6,831	$16,142	$41,510	$29,252	$7,327	$28,845	$5,584	$13,782	$2,728	$44,224	$43,966	$8,604	$92,499	$357,889
West Virginia	$1,476	$1,252	$1,314	$5,024	$10,666	$6,243	.	$3,859	$1,261	$1,945	$1,064	$8,571	$24,940	$727	$38,080	$106,420
Wisconsin	$4,660	$2,217	$3,071	.	$17,808	$11,803	.	$17,188	$1,900	$8,179	$2,594	$14,804	$32,024	$1,418	$60,901	$178,566
Wyoming	$471	$645	$285	.	$2,686	$2,004	$510	$2,869	$601	$816	$225	$3,111	$3,643	$595	$7,415	$25,877

Abbreviations: ACE = Adverse childhood experience, COPD = Chronic obstructive pulmonary disorder, DALY = Disability adjusted life year, OVOB = Overweight or obese.

© 2023 Peterson C et al. *JAMA Network Open.*

eTable 5. State-level annual ACE medical spending among adults by health condition, $ millions 2019 USD

State	Anxiety	Arthritis	Asthma	Cancer	COPD	Depression	Diabetes	Heart disease	Kidney disease	Stroke	Violence	Heavy drinking	Illicit drug use	OVOB	Smoking	Total
United States	$11,693	$22,076	$12,594	$8,713	$17,696	$49,663	$8,106	$20,739	$5,967	$9,004	$4,478	$4,501	$6,685	$333	$1,076	$183,324
Alabama	$161	$159	$67	.	$95	$387	.	$181	.	$43	$62	$42	$92	$3	$7	$1,299
Alaska	$37	$104	$51	$57	$67	$192	$114	$80	$31	$39	$14	$15	$21	<$1	$3	$826
Arizona	$198	$306	$235	$189	$284	$824	$223	$295	$34	$72	$76	$73	$113	.	$16	$2,939
Arkansas	$100	$237	$146	$73	$202	$530	$104	$266	$53	$127	$38	$58	$57	$4	$13	$2,008
California	$1,332	$3,729	$1,460	$1,124	$2,771	$6,128	$517	$3,818	$900	$716	$510	$672	$762	$55	$126	$24,621
Colorado	$166	$499	$230	$159	$357	$884	$295	$516	$134	$266	$64	$88	$95	$10	$21	$3,784
Connecticut	$162	$372	$210	$99	$300	$833	$192	$365	$101	$220	$62	$68	$92	$8	$19	$3,104
Delaware	$44	$71	$37	$52	$59	$181	.	$77	$31	$37	$17	$14	$25	$2	$4	$650
DC	$36	$52	$14	.	$21	$132	$39	$51	.	.	$14	$6	$21	.	$2	$388
Florida	$733	$1,009	$636	$307	$860	$2,488	.	$1,732	$106	$338	$281	$224	$419	.	$45	$9,178
Georgia	$303	$547	$301	$284	$394	$1,218	.	.	$97	.	$116	$120	$173	$8	$25	$3,587
Hawaii	$47	$73	$40	.	$61	$177	$67	$44	$21	.	$18	$17	$27	$3	$4	$599
Idaho	$52	$114	$50	$28	$72	$212	$82	$86	$35	$56	$20	$20	$30	$2	$5	$862
Illinois	$485	$1,248	$711	.	$1,165	$2,446	$478	$1,228	$481	$234	$186	$217	$277	$29	$61	$9,246
Indiana	$250	$464	$309	$120	$361	$1,051	$242	$557	$128	$219	$96	$90	$143	$8	$23	$4,058
Iowa	$116	$169	$84	$40	$114	$341	$74	$64	$12	.	$45	$21	$66	$4	$7	$1,157
Kansas	$101	$198	$115	$67	$154	$437	$108	$206	$39	$31	$39	$38	$58	$4	$10	$1,604
Kentucky	$161	$295	$184	$88	$219	$728	$173	$404	$85	$156	$62	$69	$92	.	$17	$2,735
Louisiana	$166	$360	$192	$126	$296	$843	$122	$330	$61	$85	$63	$88	$95	.	$20	$2,849
Maine	$58	$170	$84	$40	$114	$306	$108	$142	$40	$63	$22	$30	$33	$3	$8	$1,221
Maryland	$234	$506	$245	.	$355	$1,109	$114	$370	$99	$112	$90	$95	$134	$7	$23	$3,492
Massachusetts	$325	$663	$431	$202	$589	$1,573	$408	$653	$196	$246	$125	$127	$186	$10	$36	$5,770
Michigan	$364	$611	$361	$124	$470	$1,363	$334	$437	$92	$295	$140	$104	$208	$12	$29	$4,945
Minnesota	$221	$574	$371	$83	$482	$1,164	$236	$510	$129	$262	$85	$102	$126	$13	$29	$4,388
Mississippi	$104	$85	$69	$46	$85	$213	$46	$45	$25	$30	$40	$28	$60	$1	$6	$883
Missouri	$224	$264	$147	$157	$214	$647	.	$274	$37	$231	$86	$43	$128	$12	$14	$2,478
Montana	$38	$80	$33	$26	$58	$163	.	$65	$16	$18	$15	$13	$22	$1	$3	$552
Nebraska	$72	$204	$129	$59	$164	$385	$145	$186	$47	$81	$28	$34	$41	$3	$10	$1,590
Nevada	$87	$165	$113	.	$113	$359	.	.	$50	$40	$33	$41	$50	$4	$8	$1,062
New Hampshire	$58	$142	$76	$74	$116	$305	$110	$98	$43	$41	$22	$29	$33	$3	$8	$1,158
New Jersey	$361	$669	$418	.	$519	$1,595	$208	$199	$319	$111	$138	$139	$206	$9	$35	$4,926
New Mexico	$69	$122	$85	.	$112	$302	$39	$86	$25	$75	$26	$26	$39	.	$6	$1,013
New York	$880	$1,826	$1,001	.	$1,233	$4,091	$571	$1,251	$625	$713	$337	$371	$503	$15	$99	$13,515
North Carolina	$329	$895	$511	.	$664	$1,715	$457	$636	$143	$230	$126	$146	$188	$13	$41	$6,094
North Dakota	$33	$30	$26	$12	$28	$100	.	$15	.	.	$13	$6	$19	$1	$2	$285
Ohio	$461	$941	$649	$428	$781	$2,199	$436	$890	$223	$367	$177	$168	$264	$14	$50	$8,046
Oklahoma	$135	$208	$109	$66	$178	$521	.	$131	$41	$50	$52	$58	$77	$2	$12	$1,640
Oregon	$146	$428	$203	$105	$322	$773	$275	$257	$133	$173	$56	$75	$83	$10	$18	$3,057
Pennsylvania	$540	$895	$458	$726	$764	$1,988	.	$280	$309	$206	$207	$154	$309	$14	$45	$6,894
Rhode Island	$46	$65	$24	$20	$47	$147	$16	$53	$7	$34	$18	$13	$26	$1	$3	$519
South Carolina	$161	$159	$88	$140	$122	$456	$91	$153	$34	$132	$62	$35	$92	.	$9	$1,734
South Dakota	$35	$67	$50	$19	$56	$153	$36	$36	$11	$39	$13	$12	$20	<$1	$3	$551
Tennessee	$220	$306	$222	$155	$224	$845	$79	$367	$116	$198	$84	$83	$126	$9	$20	$3,055
Texas	$860	$1,597	$816	$638	$1,393	$3,680	.	$1,169	$391	$702	$329	$389	$492	$11	$84	$12,552
Utah	$80	$180	$112	$49	$142	$326	$68	$100	$49	$76	$31	$38	$46	$6	$9	$1,312
Vermont	$29	$72	$33	$38	$59	$152	$72	$89	$13	$22	$11	$14	$17	$2	$4	$628

© 2023 Peterson C et al. *JAMA Network Open.*

Virginia	$287	$301	$236	$133	$310	$920	.	$82	$38	$86	$110	$62	$164	$7	$13	$2,749
Washington	$255	$771	$390	$194	$609	$1,338	$405	$678	$183	$330	$98	$134	$146	$15	$29	$5,573
West Virginia	$80	$95	$78	$47	$98	$318	.	$55	$26	$35	$31	$28	$46	$1	$7	$943
Wisconsin	$229	$183	$191	.	$265	$645	.	$353	$52	$206	$88	$50	$131	$2	$17	$2,412
Wyoming	$22	$48	$19	.	$30	$96	$32	$60	$19	$21	$8	$9	$13	$1	$2	$378

Abbreviations: ACE = Adverse childhood experience, COPD = Chronic obstructive pulmonary disorder, OVOB = Overweight or obese.

© 2023 Peterson C et al. *JAMA Network Open.*

eTable 6. State-level annual and per person (affected adult) lifetime ACE economic burden by ACEs n, 2019 USD

State	Total annual, $m									Per person, $							
	DALY				Medical spending				Grand total	Annual				Lifetime			
	1	2-3	4	Any	1	2-3	4	Any		1	2-3	4	Any	1	2-3	4	Any
United States	$1,774,000	$4,051,000	$8,100,000	$13,926,000	$21,000	$57,000	$105,000	$183,000	$14,110,000	$38,000	$73,000	$144,000	$88,000	$1,056,000	$2,029,000	$4,002,000	$2,446,000
Alabama	$19,000	$47,000	$89,000	$155,000	$100	$400	$800	$1,000	$156,000	$25,000	$61,000	$112,000	$66,000	$695,000	$1,695,000	$3,113,000	$1,834,000
Alaska	$4,000	$11,000	$21,000	$36,000	$100	$300	$400	$1,000	$36,000	$56,000	$96,000	$160,000	$112,000	$1,556,000	$2,668,000	$4,447,000	$3,113,000
Arizona	$29,000	$83,000	$177,000	$289,000	$300	$900	$2,000	$3,000	$292,000	$32,000	$65,000	$124,000	$80,000	$889,000	$1,806,000	$3,446,000	$2,223,000
Arkansas	$35,000	$54,000	$123,000	$212,000	$300	$600	$1,000	$2,000	$214,000	$77,000	$131,000	$246,000	$155,000	$2,140,000	$3,641,000	$6,837,000	$4,308,000
California	$136,000	$380,000	$972,000	$1,488,000	$2,000	$7,000	$15,000	$25,000	$1,513,000	$36,000	$49,000	$126,000	$77,000	$1,000,000	$1,362,000	$3,502,000	$2,140,000
Colorado	$27,000	$74,000	$175,000	$277,000	$400	$1,000	$2,000	$4,000	$280,000	$38,000	$63,000	$146,000	$90,000	$1,056,000	$1,751,000	$4,058,000	$2,501,000
Connecticut	$34,000	$62,000	$75,000	$171,000	$700	$1,000	$1,000	$3,000	$174,000	$55,000	$106,000	$226,000	$112,000	$1,529,000	$2,946,000	$6,281,000	$3,113,000
Delaware	$7,000	$16,000	$25,000	$47,000	$100	$200	$300	$1,000	$48,000	$48,000	$96,000	$161,000	$104,000	$1,334,000	$2,668,000	$4,474,000	$2,890,000
DC	$2,000	$6,000	$10,000	$18,000	<$100	$100	$200	<$1,000	$18,000	$12,000	$47,000	$96,000	$49,000	$333,000	$1,306,000	$2,668,000	$1,362,000
Florida	$138,000	$219,000	$430,000	$786,000	$1,000	$3,000	$5,000	$9,000	$796,000	$39,000	$69,000	$121,000	$76,000	$1,084,000	$1,918,000	$3,363,000	$2,112,000
Georgia	$48,000	$97,000	$227,000	$372,000	$500	$1,000	$2,000	$4,000	$375,000	$30,000	$59,000	$134,000	$75,000	$834,000	$1,640,000	$3,724,000	$2,084,000
Hawaii	$5,000	$11,000	$25,000	$41,000	$100	$200	$400	$1,000	$41,000	$21,000	$49,000	$118,000	$61,000	$584,000	$1,362,000	$3,279,000	$1,695,000
Idaho	$5,000	$17,000	$43,000	$64,000	$100	$200	$500	$1,000	$65,000	$18,000	$60,000	$131,000	$74,000	$500,000	$1,667,000	$3,641,000	$2,057,000
Illinois	$106,000	$220,000	$314,000	$641,000	$1,000	$3,000	$4,000	$9,000	$650,000	$39,000	$97,000	$165,000	$93,000	$1,084,000	$2,696,000	$4,586,000	$2,585,000
Indiana	$36,000	$90,000	$202,000	$329,000	$400	$1,000	$2,000	$4,000	$333,000	$40,000	$84,000	$173,000	$105,000	$1,112,000	$2,334,000	$4,808,000	$2,918,000
Iowa	$9,000	$24,000	$46,000	$80,000	$100	$400	$700	$1,000	$81,000	$18,000	$49,000	$109,000	$56,000	$500,000	$1,362,000	$3,029,000	$1,556,000
Kansas	$11,000	$35,000	$76,000	$122,000	$200	$500	$1,000	$2,000	$124,000	$31,000	$72,000	$135,000	$87,000	$862,000	$2,001,000	$3,752,000	$2,418,000
Kentucky	$28,000	$70,000	$193,000	$292,000	$200	$700	$2,000	$3,000	$295,000	$66,000	$100,000	$202,000	$140,000	$1,834,000	$2,779,000	$5,614,000	$3,891,000
Louisiana	$41,000	$73,000	$157,000	$270,000	$300	$800	$2,000	$3,000	$273,000	$60,000	$109,000	$207,000	$128,000	$1,667,000	$3,029,000	$5,753,000	$3,557,000
Maine	$11,000	$23,000	$58,000	$92,000	$100	$400	$700	$1,000	$93,000	$54,000	$85,000	$184,000	$117,000	$1,501,000	$2,362,000	$5,114,000	$3,252,000
Maryland	$21,000	$67,000	$144,000	$231,000	$300	$1,000	$2,000	$3,000	$235,000	$29,000	$73,000	$134,000	$86,000	$806,000	$2,029,000	$3,724,000	$2,390,000
Massachusetts	$30,000	$85,000	$192,000	$306,000	$500	$2,000	$3,000	$6,000	$312,000	$35,000	$76,000	$154,000	$96,000	$973,000	$2,112,000	$4,280,000	$2,668,000
Michigan	$40,000	$126,000	$245,000	$411,000	$500	$2,000	$3,000	$5,000	$416,000	$24,000	$74,000	$127,000	$78,000	$667,000	$2,057,000	$3,530,000	$2,168,000
Minnesota	$44,000	$75,000	$123,000	$242,000	$800	$1,000	$2,000	$4,000	$247,000	$42,000	$103,000	$166,000	$96,000	$1,167,000	$2,863,000	$4,613,000	$2,668,000
Mississippi	$16,000	$25,000	$58,000	$99,000	$100	$200	$500	$1,000	$100,000	$31,000	$57,000	$151,000	$74,000	$862,000	$1,584,000	$4,197,000	$2,057,000
Missouri	$27,000	$70,000	$136,000	$233,000	$300	$800	$1,000	$2,000	$235,000	$26,000	$69,000	$129,000	$76,000	$723,000	$1,918,000	$3,585,000	$2,112,000
Montana	$4,000	$12,000	$30,000	$45,000	<$100	$200	$400	$1,000	$45,000	$26,000	$57,000	$132,000	$79,000	$723,000	$1,584,000	$3,668,000	$2,196,000
Nebraska	$10,000	$36,000	$50,000	$96,000	$200	$600	$800	$2,000	$97,000	$35,000	$105,000	$190,000	$106,000	$973,000	$2,918,000	$5,280,000	$2,946,000
Nevada	$9,000	$34,000	$85,000	$129,000	$100	$300	$700	$1,000	$130,000	$29,000	$66,000	$110,000	$80,000	$806,000	$1,834,000	$3,057,000	$2,223,000
New Hampshire	$11,000	$21,000	$51,000	$83,000	$100	$400	$700	$1,000	$84,000	$57,000	$89,000	$213,000	$124,000	$1,584,000	$2,473,000	$5,920,000	$3,446,000
New Jersey	$42,000	$110,000	$155,000	$307,000	$600	$2,000	$2,000	$5,000	$312,000	$32,000	$73,000	$148,000	$79,000	$889,000	$2,029,000	$4,113,000	$2,196,000
New Mexico	$8,000	$34,000	$54,000	$96,000	$100	$400	$600	$1,000	$97,000	$32,000	$86,000	$127,000	$90,000	$889,000	$2,390,000	$3,530,000	$2,501,000
New York	$111,000	$236,000	$416,000	$764,000	$2,000	$4,000	$8,000	$14,000	$778,000	$40,000	$82,000	$143,000	$89,000	$1,112,000	$2,279,000	$3,974,000	$2,473,000
North Carolina	$54,000	$187,000	$305,000	$546,000	$400	$2,000	$3,000	$6,000	$552,000	$36,000	$98,000	$176,000	$107,000	$1,000,000	$2,724,000	$4,891,000	$2,974,000
North Dakota	$1,000	$4,000	$10,000	$15,000	<$100	$100	$200	<$1,000	$15,000	$11,000	$37,000	$95,000	$45,000	$306,000	$1,028,000	$2,640,000	$1,251,000
Ohio	$75,000	$215,000	$382,000	$672,000	$800	$3,000	$5,000	$8,000	$680,000	$47,000	$106,000	$189,000	$119,000	$1,306,000	$2,946,000	$5,253,000	$3,307,000
Oklahoma	$20,000	$41,000	$124,000	$185,000	$100	$400	$1,000	$2,000	$187,000	$39,000	$78,000	$153,000	$100,000	$1,084,000	$2,168,000	$4,252,000	$2,779,000
Oregon	$20,000	$64,000	$138,000	$223,000	$200	$1,000	$2,000	$3,000	$226,000	$32,000	$74,000	$161,000	$94,000	$889,000	$2,057,000	$4,474,000	$2,612,000
Pennsylvania	$80,000	$159,000	$323,000	$562,000	$1,000	$2,000	$4,000	$7,000	$568,000	$42,000	$78,000	$141,000	$90,000	$1,167,000	$2,168,000	$3,919,000	$2,501,000
Rhode Island	$7,000	$9,000	$21,000	$36,000	$100	$100	$300	$1,000	$37,000	$34,000	$54,000	$144,000	$71,000	$945,000	$1,501,000	$4,002,000	$1,973,000
South Carolina	$14,000	$53,000	$108,000	$175,000	$100	$600	$1,000	$2,000	$177,000	$16,000	$65,000	$125,000	$68,000	$445,000	$1,806,000	$3,474,000	$1,890,000
South Dakota	$3,000	$14,000	$16,000	$33,000	$100	$200	$300	$1,000	$33,000	$27,000	$73,000	$149,000	$80,000	$750,000	$2,029,000	$4,141,000	$2,223,000
Tennessee	$31,000	$91,000	$229,000	$351,000	$200	$900	$2,000	$3,000	$354,000	$33,000	$87,000	$158,000	$102,000	$917,000	$2,418,000	$4,391,000	$2,835,000
Texas	$99,000	$299,000	$621,000	$1,020,000	$800	$4,000	$8,000	$13,000	$1,032,000	$28,000	$59,000	$131,000	$76,000	$778,000	$1,640,000	$3,641,000	$2,112,000
Utah	$8,000	$32,000	$59,000	$99,000	$100	$400	$800	$1,000	$100,000	$22,000	$48,000	$106,000	$63,000	$611,000	$1,334,000	$2,946,000	$1,751,000

© 2023 Peterson C et al. *JAMA Network Open.*

Vermont	$7,000	$11,000	$22,000	$39,000	$100	$200	$300	$1,000	$40,000	$67,000	$93,000	$190,000	$119,000	$1,862,000	$2,585,000	$5,280,000	$3,307,000
Virginia	$27,000	$56,000	$126,000	$209,000	$400	$800	$2,000	$3,000	$212,000	$18,000	$40,000	$114,000	$52,000	$500,000	$1,112,000	$3,168,000	$1,445,000
Washington	$39,000	$116,000	$204,000	$358,000	$600	$2,000	$3,000	$6,000	$363,000	$34,000	$72,000	$137,000	$84,000	$945,000	$2,001,000	$3,807,000	$2,334,000
West Virginia	$11,000	$24,000	$71,000	$106,000	$100	$200	$600	$1,000	$107,000	$52,000	$94,000	$201,000	$129,000	$1,445,000	$2,612,000	$5,586,000	$3,585,000
Wisconsin	$24,000	$58,000	$96,000	$179,000	$200	$900	$1,000	$2,000	$181,000	$25,000	$61,000	$110,000	$64,000	$695,000	$1,695,000	$3,057,000	$1,779,000
Wyoming	$2,000	$7,000	$16,000	$26,000	<$100	$100	$200	<$1,000	$26,000	$28,000	$79,000	$144,000	$90,000	$778,000	$2,196,000	$4,002,000	$2,501,000

Abbreviations: ACE = Adverse childhood experience, DALY = Disability adjusted life year.

© 2023 Peterson C et al. *JAMA Network Open.*
