# Supplementary material for: Economic Burden of Health Conditions Associated With Adverse Childhood Experiences Among US Adults
Source: JAMA Netw Open. 2023 Dec 6;6(12):e2346323. doi: 10.1001/jamanetworkopen.2023.46323 (PMC10701608; doi:10.1001/jamanetworkopen.2023.46323)
Supplement: Supplement 2. — Data Sharing Statement [file jamanetwopen-e2346323-s002.pdf]

Data Sharing Statement

Peterson. Economic Burden of Health Conditions Associated With Adverse Childhood Experiences Among US Adults. *JAMA Netw Open*. Published December 06, 2023. doi:10.1001/jamanetworkopen.2023.46323

Data
Data available: Yes
Data types: Data (not involving human participants)
How to access data: BRFSS annual survey data: https://www.cdc.gov/brfss/annual_data/annual_data.htm Global Burden of Disease study Results Tool: https://vizhub.healthdata.org/gbd-results/
When available: With publication

Supporting Documents
Document types: None

Additional Information
Who can access the data: Anyone requesting the data
Types of analyses: Any purpose
Mechanisms of data availability: Without investigator support
Any additional restrictions: None
